# Surface model of the human red blood cell simulating changes in membrane curvature under strain

**DOI:** 10.1038/s41598-021-92699-7

**Published:** 2021-07-01

**Authors:** Philip W. Kuchel, Charles D. Cox, Daniel Daners, Dmitry Shishmarev, Petrik Galvosas

**Affiliations:** 1 School of Life and Environmental Sciences, University of Sydney, Building G08, Sydney, NSW 2006 Australia; 2 Victor Chang Cardiac Research Institute, Darlinghurst, Sydney, NSW Australia; 3 St Vincent’s Clinical School, Faculty of Medicine, University of New South Wales, Sydney, NSW Australia; 4 School of Mathematics and Statistics, University of Sydney, Sydney, NSW Australia; 5 John Curtin School of Medical Research, Australian National University, Canberra, ACT Australia; 6 MacDiarmid Institute for Advanced Materials and Nanotechnology, School of Chemical and Physical Sciences, Victoria University Wellington, Wellington, New Zealand

**Keywords:** Biophysics, Cell biology

## Abstract

We present mathematical simulations of shapes of red blood cells (RBCs) and their cytoskeleton when they are subjected to linear strain. The cell surface is described by a previously reported quartic equation in three dimensional (3D) Cartesian space. Using recently available functions in *Mathematica* to triangularize the surfaces we computed four types of curvature of the membrane. We also mapped *changes* in mesh-triangle area and curvatures as the RBCs were distorted. The highly deformable red blood cell (erythrocyte; RBC) responds to mechanically imposed shape changes with enhanced glycolytic flux and cation transport. Such morphological changes are produced experimentally by suspending the cells in a gelatin gel, which is then elongated or compressed in a custom apparatus inside an NMR spectrometer. A key observation is the extent to which the maximum and minimum Principal Curvatures are localized symmetrically in patches at the poles or equators and distributed in rings around the main axis of the strained RBC. Changes on the nanometre to micro-meter scale of curvature, suggest activation of only a subset of the intrinsic mechanosensitive cation channels, Piezo1, during experiments carried out with controlled distortions, which persist for many hours. This finding is relevant to a proposal for non-uniform distribution of Piezo1 molecules around the RBC membrane. However, if the curvature that gates Piezo1 is at a very fine length scale, then membrane tension will determine local curvature; so, curvatures as computed here (in contrast to much finer surface irregularities) may not influence Piezo1 activity. Nevertheless, our analytical methods can be extended address these new mechanistic proposals. The geometrical reorganization of the simulated cytoskeleton informs ideas about the mechanism of concerted metabolic and cation-flux responses of the RBC to mechanically imposed shape changes.

## Introduction

Our aim is to convey a sense of scale in the distribution of proteins in the membrane and subjacent cytoskeleton, relative to the whole human red blood cell (RBC); and to graphically represent changes in membrane curvature on the ~ 1 nm to ~ 10 μm scale, brought about by the systematic straining of these cells. This study was motivated by the quest for the geometrical and mechanistic basis of recent findings on mechanically distorted RBCs, made by using nuclear magnetic resonance (NMR) spectroscopy with stretched and compressed gels^1,2^.

The rates of glycolysis and transmembrane exchange of cations in RBCs are enhanced when these cells, suspended in gelatin gel, are stretched or compressed. The metabolic effect occurs only when the medium contains Ca^2+^ ions; and the cells' responses are attributed to the activation of the mechanosensitive cation channel, Piezo1, when the plasma membrane is distorted^3–7^.

Cation-flux estimates via Piezo1 are typically made by electrophysiological means. This has been by classical patch clamping^8^, or in native RBCs via high throughput planar patch clamp measurements^9^. Because the electrophysiological measurements usually occur over seconds (of a transient response to shape change), while the NMR experiments can last for hours, a different property of channel opening and closure is in operation^1,2,10^. Under constant stretch, even for a channel that inactivates, a steady state current persists. With inactivating channels like Piezo1, this current is much smaller than the peak current generated by mechanical deformation. Thus, for long periods of stretch in NMR experiments, the results more closely correspond to the steady state Piezo1 current that occurs under constant mechanical load.

The Piezo1 three-arm (triskelion) structure spans a relatively large ~ 23 nm diameter membrane patch; this has been measured from images obtained with cryo-electron microscopy^11–13^. Such imaging provides an indication of the likely values of curvature that are required to activate the channel. The current model suggests that the channel is curved in the resting state and flattens as membrane tension increases, which opens the channel^14^. Therefore, when inspecting the alteration of the average extent of membrane curvature, when an RBC is distorted, we might expect an increase in the fraction of the total membrane area that has lower curvature; since such altered topology (induced flatness) appears to cause activation of Piezo1.

To quantify the alteration of membrane curvature that is brought about by stretching an idealized RBC, we employed a geometrical *strain field*, in which an RBC is elongated by a specified extent, denoted by ξ. In addition, we considered the variation of the angle of rotation of RBCs relative to the direction of the imposed strain field, prior to "switching the strain field on" (imposing stretch). This is relevant to describing the state-of-affairs of RBCs suspended in liquid gelatin prior to gelation, because the cells are known to have random orientations, which persist while in the gel state^15^. The mathematical model used in these analyses captures the key micro-anatomical dimensions of the discocyte that typify a human RBC (and in most other mammals, other than the camelidae)^16^.

Mathematical definitions of curvature of three dimensional (3D) surfaces are a major concern of differential geometry^17,18^; it is obvious that complicated formulae have become more accessible since the advent of symbolic computation, most notably in software packages like *Mathematica*^19,20^. Euler rotation matrices and strain tensors were applied to bring about the simulated RBC distortions (morphing), taking care to invoke the relevant inverse functions in the definition of the transformed shape function, and the curvature functions; again, *Mathematica* generated algebraic expressions symbolically. This remarkable outcome, despite the highly complicated forms, meant that the expressions were accurately evaluated to give estimates of curvatures. The ability to triangularize* the mathematically specified surface of the RBC (in *Mathematica*) meant that the relative size of the cytoskeletal triangular (also referred to as hexagonal) mesh was able to be visualized in practicable computation times (minutes)^19^. (*Aside: we use the term triangular*ization* as opposed to triangulation to distinguish the operation from the trigonometric procedure used in surveying, and cartography etc).

## Theory of methods

### RBC shape

**Figure 1:**
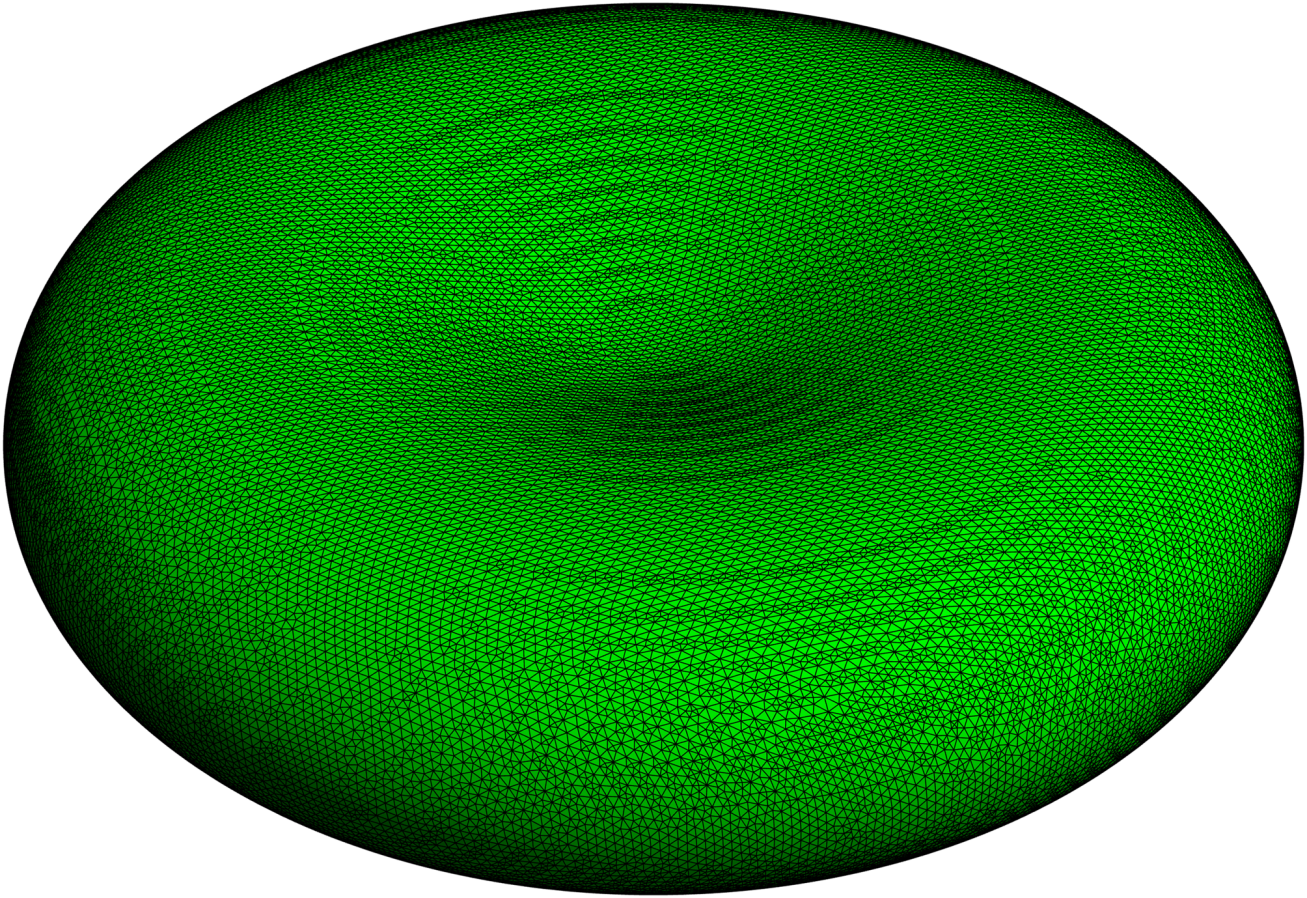
Triangular mesh of the RBC drawn to scale in its biconcave disc, using Eq. (1) in *Mathematica*. The main diameter *d* = 8 μm, thickness at the dimple *b* = 1 μm, and maximum height at the rim *h* = 2.12 μm. The triangularization was with BoundaryDiscretizationRegion, applied to ImplicitRegion (Supplementary Information). Number of edges = 120,042 and MeshCoordinates gave 40,136 points and 80,268 triangles. Green colouring was chosen over red (the natural choice for an RBC) for ease of visualizing the mesh. Notebook 2 has the *Mathematica* script used to generate this graphic.

There have been several expressions presented for the shape of the RBC including one based on the minimization of the bending energy of a dual layer membrane^21^. The mathematical expression for the RBC discocyte used here is close to those depicted in^21^, and it is a continuous degree-4 surface that can be written either in Cartesian or disc-cyclide coordinates, making it versatile for numerical exploration^16^. The shape is constrained by three principal distances, the main diameter, *d*, the thickness at the centre of the dimples, *b*, and the maximum thickness (height) near the periphery of the cell, *h* (see Fig. 1 for the first of many examples here):

1 $${\left({x}^{2}+{y}^{2}+{z}^{2}\right)}^{2}+P({x}^{2}+{y}^{2})+Q{z}^{2}+R=0$$

where,

2 $$P=-\frac{{d}^{2}}{2}+\frac{{h}^{2}}{2}\left(\frac{{d}^{2}}{{b}^{2}}-1\right)-\frac{{h}^{2}}{2}\left(\frac{{d}^{2}}{{b}^{2}}-1\right)\left(1-\frac{{b}^{2}}{{h}^{2}}\right)^{\frac{1}{2}}$$

3 $$Q=P\frac{{d}^{2}}{{b}^{2}}+\frac{{b}^{2}}{4}\left(\frac{{d}^{4}}{{b}^{4}} - 1\right)$$

4 $$R=-P\frac{{d}^{2}}{4} - \frac{{d}^{4}}{16}$$

The mean volume of a normal human RBC is 86 fL, while the surface area is variously stated to be 137 ± 17 or 143 µm^2^^22,23^. Thus, when *d* = 8 µm, *b* = 1 µm, and *h* = 2.12 µm, the model gives a volume of 86 fL and a surface area of 128 µm^2^. We gave precedence to the correct volume over the predicted smaller surface area in the above range of 120–154 µm^2^.

### Triangularization of the surface

While Eq. (1) is readily graphed in *Mathematica*, a representation of the cell's cytoskeleton requires partitioning the surface with a known number of struts (edges) in the geodetic-dome-like closed polyhedron. In a human RBC, the struts of the mesh consist of head-to-head-associated two hetero-dimers of α- and β-spectrin, with junctional complexes of 12–14 actin monomers bound as short, twisted filaments. There are ~ 121,000 edges^24^ so in *Mathematica* the Option, MaxCellMeasure in the BoundaryDiscretizeRegion function could be manually adjusted to make the number of edges in the polyhedron very close to this value (see the Supplementary Information for a Notebook implementation).

### Shape transformation

We emulated the distortion of RBCs suspended in stretched gelatin gel by specifying that a geometrical strain field was applied in one direction, chosen to be along the *z*-axis. This equates to what applies in real NMR experiments^1,2^. It is not possible, experimentally, to align all the RBCs in gelatin media prior to or after gelation, so the members of the population of RBCs assume all possible orientations of their axes of symmetry in the strain field. Therefore, we considered three orientations as *representative* of all those that are possible (see “Discussion” for additional comments).

#### Euler rotation matrix

To alter RBC orientation, the independent variables in Eq. (1) were transformed, by rotation about the *x*-axis by an angle θ, using an Euler rotation matrix^25^:

5 $$R_{\theta } = \left( {\begin{array}{*{20}c} 1 & 0 & 0 \\ 0 & {\text{cos}\uptheta } & { - \text{sin}\uptheta } \\ 0 & {\text{sin}\uptheta } & {\text{cos}\uptheta } \\ \end{array} } \right)$$

where θ = 0 specifies the original orientation.

#### Strain tensor

This mathematical object is a matrix that invokes volume preserving elongation of a Cartesian body in the direction of the *z*-axis:

6 $$S_{{\xi}} = ~\left( {\begin{array}{*{20}c} {\frac{1}{{\sqrt {\xi} }}} & 0 & 0 \\ 0 & {\frac{1}{{\sqrt {\xi} }}} & 0 \\ 0 & 0 & {\xi} \\ \end{array} } \right)$$

where ξ = 1 specifies the original shape.

#### Inverse affine transformation of Eq. (1)

The requisite overall coordinate-transformation matrix was derived symbolically in *Mathematica* with the following function (note the standard matrix product denoted by **.**).

$${\text{trf}} = {\text{InverseFunction[AffineTransform}}[S_{\xi } \cdot R_{\theta } ]].$$

followed by 'threading' the transformation through the discocyte expression (see the Supplementary Information for further explanation of the symbols and the complete *Mathematica* Notebook):7and this yielded the shape-transformed Eq. (1):8

Then, the triangularization of the surface was performed as follows:9

The function RegionMeasure applied to the boundary-discretized region gave the RBC volume; and the functions RegionMeasure[RegionBoundary[bmr0]] yielded the area of the RBC. The coordinates of the nodes of the mesh, and the list of all triangles, were obtained with the functions MeshCoordinates and MeshPrimitives. Finally, MeshTriangles was plotted by using Graphics3D (Fig. 1).

### Curvature

Representing shape and curvature are primary objectives of differential geometry^17^, and modern computation with *Mathematica* provides a way of generating curvature expressions for surfaces that are defined implicitly by equations like Eq. (1). And, even more remarkably, those that are transformed to complicated expressions like Eq. (8).

A non-planar surface in three dimensions has a tangent plane and a normal vector at a specified point. In general, the curvature of the surface differs in one direction versus one at right angles to it. The shapes of these surfaces can be illustrated with the particular example of the hyperbolic paraboloid (saddle)^17^. Such a graphical rendering is shown in Supplementary Information, Fig. S1. The observation of, in general, two Principal Curvatures motivates the implementation of expressions that describe the curvature of the surface at a given point on the RBC. The fact that there are several ways of describing curvature of a surface may not be immediately obvious; but in general there are four expressions that have been explored in the theory of 3D differential geometry^17^.

### Curvature expressions

We begin the presentation of the operations that are required to generate the types of curvatures of the RBC surface by defining *F*[*x,y,z*] from Eq. (1):

10 $${F\left[x,y,z\right] \equiv \left({x}^{2}+{y}^{2}+{z}^{2}\right)}^{2}+P\left({x}^{2}+{y}^{2}\right)+Q{z}^{2}+R$$

Four operations are to be carried out on $F\left[x,y,z\right]$ to make up the requisite expressions: (1) The gradient of *F*, $\nabla F\left[x,y,z\right],$ is a vector of partial derivatives of *F* with respect to each of the independent Cartesian variables:

11 $$\nabla F\left[x,y,z\right]={(F}_{x},{F}_{y},{F}_{z})$$

(2) The Hessian of *F, H*[*F*], is a 3 × 3 matrix of second order partial derivatives of *F*:

12 $$H\left[F\right]=\left(\begin{array}{ccc}{F}_{xx}& {F}_{xy}& {F}_{xz}\\ {F}_{yx}& {F}_{yy}& {F}_{yz}\\ {F}_{zx}& {F}_{zy}& {F}_{zz}\end{array}\right)$$

(3) The cofactor or adjugate matrix, denoted by H*[*F*] is defined as:

13 $$H^{*} \left[ F \right] = \left( {\begin{array}{*{20}l} {{\text{Cofactor}}(F_{{xx}} )} \hfill & {{\text{Cofactor}}(F_{{xy}} )} \hfill & {{\text{Cofactor}}\left( {F_{{xz}} } \right)} \hfill \\ {{\text{Cofactor}}(F_{{yx}} )} \hfill & {{\text{Cofactor}}\left( {F_{{yy}} } \right)} \hfill & {{\text{Cofactor}}(F_{{yz}} )} \hfill \\ {{\text{Cofactor}}(F_{{zx}} )} \hfill & {{\text{Cofactor}}(F_{{zy}} )} \hfill & {{\text{Cofactor}}(F_{{zz}} )} \hfill \\ \end{array} } \right)$$

where ${{\text{Cofactor}}(F_{{ab}} )}, \, a, \,b = x, \,y, \,or \, z,$ for each of the second derivatives in Eq. (13) is a matrix of determinants^26^.

(4) Finally, the trace of the Hessian matrix is required; this is simply the sum of the three terms in the leading diagonal of *H*[*F*] [Eq. (13)]; it is the Laplace operator of *F*.

#### Gaussian curvature K_G_

The Gaussian Curvature is expressed in terms of the vector of partial derivatives, its transpose, and the cofactor matrix of the Hessian^17,18^;

14 $${\mathrm{K}}_{\mathrm{G}}=\frac{\nabla F \cdot {H}^{*}[F] \cdot {\nabla F}^{\mathrm{T}}}{{\left| \nabla F\right|}^{4}}$$

#### Mean curvature K_M_

This is given by,

15 $${\text{K}}_{{\text{M}}} = ~\frac{{\nabla F \cdot H\left[ F \right]~\cdot~\nabla F{\text{~}}^{{\text{T}}} - \left| F \right|^{2} Trace\left[ H \right]}}{{2~\left| \nabla F \right|^{3} }}$$

#### Principal curvatures k_1_ and k_2_

They are the largest and smallest curvatures at a given point. It appears obtuse to define the Principal Curvatures after the other two, but it is computationally more efficient to do so^17,18^:

16 $${\mathrm{k}}_{1}={\mathrm{K}}_{\mathrm{M}}+\sqrt{{{\mathrm{K}}_{\mathrm{M}}}^{2} - {\mathrm{K}}_{\mathrm{G}}}$$

17 $${\mathrm{k}}_{2}={\mathrm{K}}_{\mathrm{M}}-\sqrt{{{\mathrm{K}}_{\mathrm{M}}}^{2} - {\mathrm{K}}_{\mathrm{G}}}$$

The relationships are ${\mathrm{K}}_{\mathrm{G}}={\mathrm{k}}_{1}{\mathrm{k}}_{2}$, and ${\mathrm{K}}_{\mathrm{M}}=\frac{{\mathrm{k}}_{1}+{\mathrm{k}}_{2}}{2}$, the latter explaining the term Mean Curvature.

### Implementation of Eqs. (15)–(18) for the RBC

#### Average at the three vertices

The next step after triangularization (e.g., Fig. 1) was to assign the values of curvature to each triangular face. This was done by applying Eqs. (14)–(17) to the vertices of each of the triangles and then averaging the three values.

#### Average at the centroid

An alternative treatment was to determine the positions of the centroid (centre of gravity) of each triangle and apply Eqs. (14)–(17) to those:

18 $${\text{Centroid}}\left[ {x,y,z} \right]{\text{ }} = {\text{ }}\left( {{\text{vertex1}}\left[ {x,y,z} \right]{\text{ }} + ~{\text{vertex2}}\left[ {x,y,z} \right]{\text{ }} + {\text{ vertex3}}\left[ {x,y,z} \right]{\text{ }}} \right)/3$$

#### Weighted average curvatures

**Figure 2:**
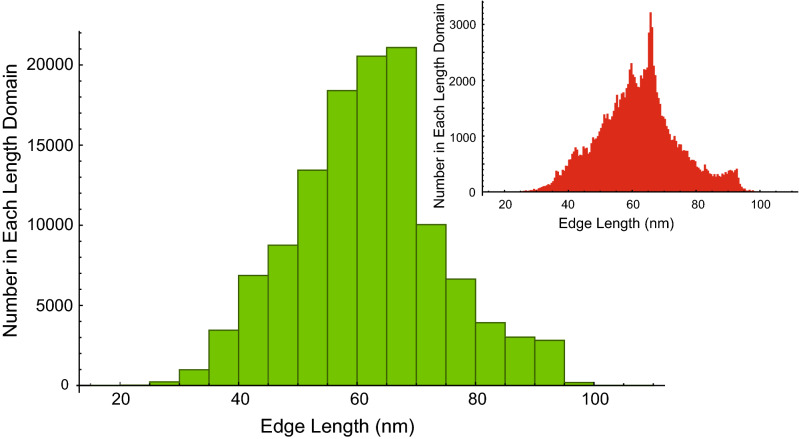
Data from the mesh in Fig. 1 showing the length-distribution histogram of edges in 20 bins (green); and, 200 bins (red inset). The mean edge length overall was 62 nm. The median bin (tallest green pillar) contained 38% of the total bin contents and spanned 60–65 nm; while the *two* most abundant bins spanning 60–70 nm contained 75% of the edge lengths. Notebook 2 has the *Mathematica* script used to generate this graphic.

Because the area of the triangles in any triangularization vary, as seen in the histogram of Fig. 2, the average of the curvatures of a set of triangles must be the weighted average. The weighting factor is the area of the triangle relative to the total area of all the triangles in the set:

19 $${\text{wt}}\,{\text{AverageCurvature}} = \frac{{\mathop \sum \nolimits_{{i = 1}}^{N} {\text{area}}_{i} ~{\text{curvature}}_{i} }}{{\mathop \sum \nolimits_{{i = 1}}^{N} {\text{area}}_{i} ~}}$$

where the area of each triangle is given by the ‘cross product formula’ from vector analysis^27^. Specifically, the differences between the position vectors of each vertex, v1, v2, v3 are the side vectors of the triangle, × denotes the vector cross product, and | | denotes the norm:

20 $${\text{Area }} = \left( {{\text{1}}/{\text{2}}} \right){\text{ }}|\left( {{\text{v2 }}{-}{\text{ v1}}} \right) \times \left( {{\text{v3 }}{-}{\text{ v1}}} \right)|$$

## Results

### Triangularization

#### Euler test

The triangularization shown in Fig. 1 was closed (no gaps), with the number of edges E, faces (F; triangles) and vertices (V; mesh points) conforming to Euler's formula F − E + V = 2^28^; the 2 is a topological invariant called the ‘Euler characteristic’ and is typically denoted by χ.

#### Gauss–Bonnet theorem test

The Total Curvature, which is the integral of the values of the Gaussian Curvature, K_G_, over a closed surface (like that used to describe the RBC), evaluates to 4π; specifically it is 2π χ^17^. This was indeed closely approximated by summing the product of K_G_ (the mean of the three values of each triangle) and its area, across all triangles in the mesh. An example of the analysis is given in Notebook 5, Supplementary Information.

#### Triangles per mesh point

Detailed inspection of the mesh showed that in the vast majority of cases a single mesh point was met by six triangles; but there were a few instances of five and seven triangles sharing one vertex. These points appeared to be randomly dispersed on the surface. If the instances of such sharp triangles were high, this could cause problems with the finite element approximation of the surface area; but there was not a problem as noted above in relation to the Gauss-Bonnet Theorem test. On the other hand, such aberrant triangles are not a "good" representation of the spectrin mesh according to recent microscopic image analysis^24,29^.

#### Orientation

It was important to test the fidelity to the triangularization algorithm in generating the same RBC volume and area, and curvature estimates, when the RBC was rotated about the *x*-axis (and, by symmetry, any other rotations about lines through the origin in the *x*,*y*-plane) in the Cartesian coordinate system using Eq. (5). Specifically, the distribution of edge-lengths should not change when θ is varied, and this was reliably achieved.

#### Edge length

The next step was to compute the distribution of edge lengths, in order to study how these edges, which could be thought of as modelling the spectrin network, might imply that the spectrin is either stretched or compressed. Figure 2 shows the distribution of sides in Fig. 1, first at a resolution of 20 linearly spaced bins. The distribution is slightly skewed to the left, but it is unimodal; however, the inset which was based on 200 bins now appears to be at least trimodal (see “Discussion”).

#### Triangle area distribution

Another feature of the surface triangularization, that is of biophysical importance, is the area of each triangle and whether this is consistent with the known span of membrane proteins that are corralled in the network. Figure S2a shows the separate triangularization of the RBC, which (as for Fig. 1) clearly indicates a range of shapes and sizes of the triangles. Sorting the triangles according to area showed a span from 0.0094 to 3431 nm^2^. When subdivided linearly into 10 bins it was seen that the 6th bin contained the most triangles (27,541) with a mean area of 1873 nm^2^; in other words 40.3% of the total area of 128 μm^2^ had this mean area, while the mean area of a triangle across the whole cell was 1575 nm^2^. The size distribution is shown graphically in Fig. S3.

### Curvature mapping

**Figure 3:**
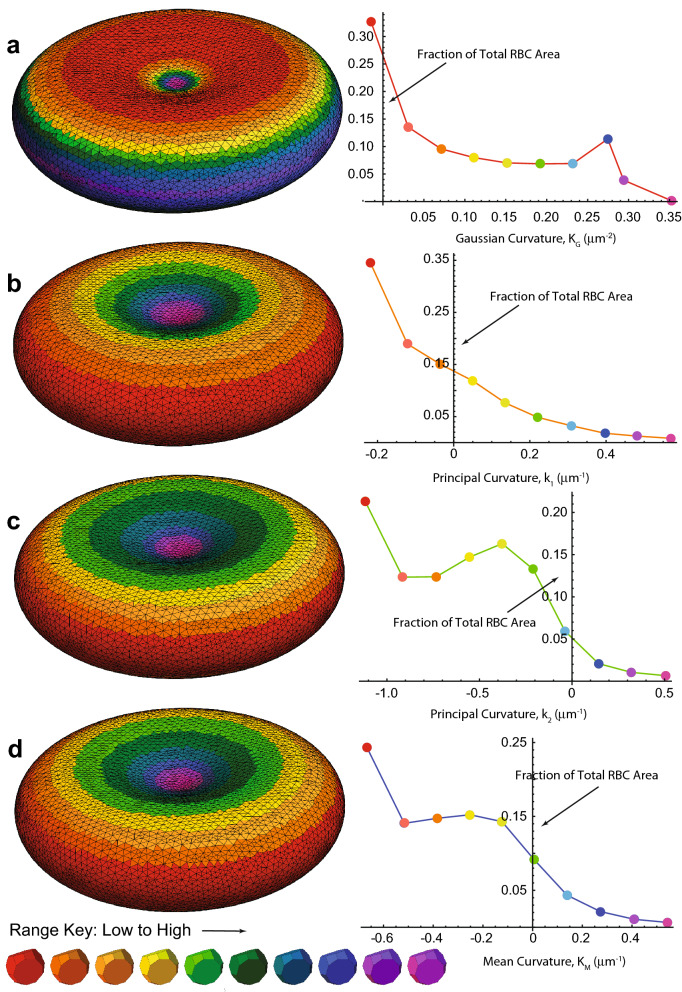
Positional dependence of the four types of curvature on the surface of the model RBC. (**a**) Gaussian Curvature, K_G_; (**b**) Principal Curvature (maximum), k_1_; (**c**) Principal Curvature (minimum), k_2_; and (**d**) Mean Curvature, K_M_. On the right of each cell is the graph of mean value (of the respective curvature) versus the fraction of the RBC area that has the curvature in a specified sub-domain of values. Specifically, the minimum and maximum values of each curvature were idenified, then the whole domain of values was divided linearly into 10 sub-domains (bins) with each assigned a colour-code, as shown in the given Range Key. The area of each triangle was computed (“Theory of Methods”; and Notebook 2), so the total area occupied by triangles in a given bin could be expressed as a fraction of the total RBC area, 128 μm^2^. For speed of computation the triangularization was made with fewer mesh points than for Fig. 1, specifically 13,640 triangles and 6822 mesh points.

A primary aim was to devise a means of displaying (mapping) the distribution of curvature(s) on the surface of the RBC. For this, a colour-coding program was written. Each triangle from the triangularization was stored in one of 10 value domains, according to whichever of the attributes was to be mapped. Figure 3 shows an undistorted RBC with its axis of symmetry normal to the the *x,y*-plane, and for which the average of the curvatures at each of the three vertices of each triangle was assigned.

### Rotation and strain

**Figure 4:**
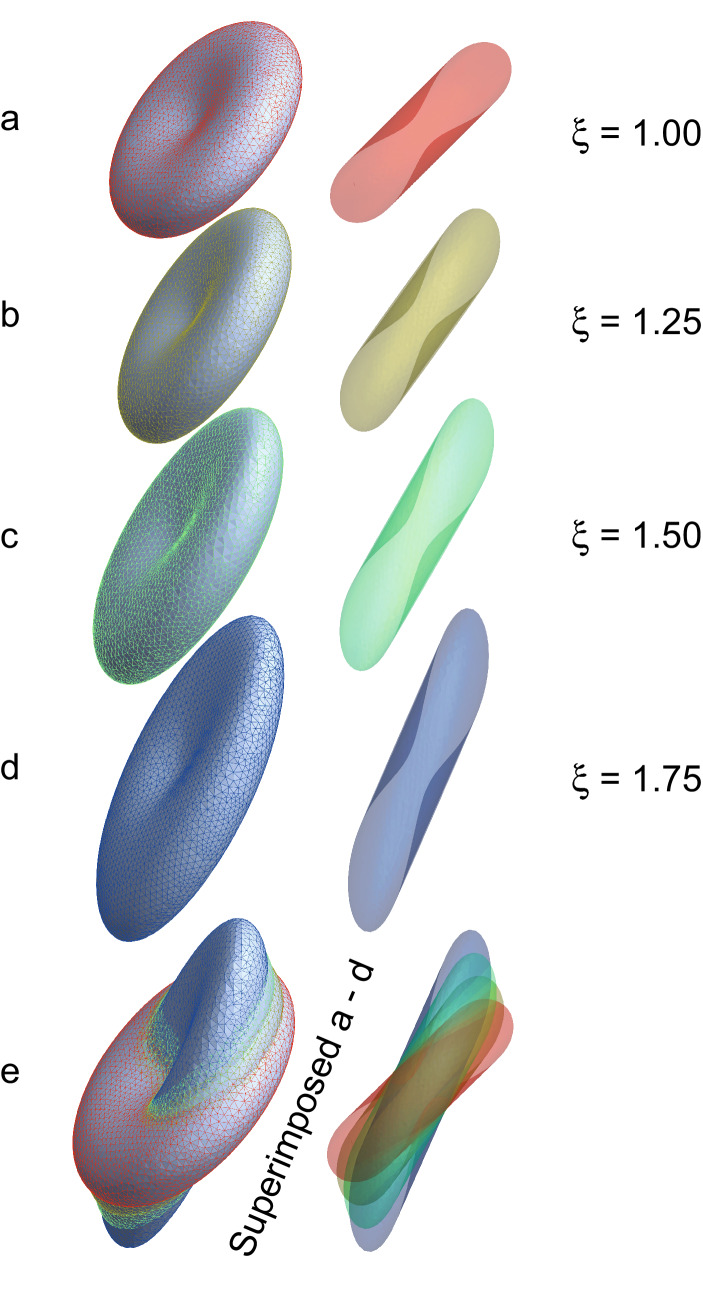
RBC rotated through 45° about the x-axis, relative to a linear strain field imposed in the *z*-direction. The relaxed RBC (**a**) had the same dimensions as in Fig. 1 (in fact, as in all the figures in this article): (**a**) No elongation, ξ = 1.00; (**b**) stretched by 25%, ξ = 1.25; (**c**) stretched by 50%, ξ = 1.50; (**d**) stretched by 75%, ξ = 1.75; and (**e**) showing the relative elongation and concomitant narrowing of the RBCs by superimposing the images. Colour coding was used to provide distinction between the RBCs in (**e**). In the boundary discretization MaxCellMeasure was set to 0.1 giving ~ 8000 mesh points.

The affine transformation, which combines both rotation and strain, produced shape changes such as those shown in Fig. 4. For this figure the RBC was rotated by 45° from the *x*,*y*-plane and then increasingly stretched.

On stretching (in the z-direction) the RBC was elongated and became narrower, an effect best seen in the overlapping images in Fig. 4e. However, the width of the RBC at the dimples and the maximum curvature at the rim both increased, as if the opposite faces of the cell were being pulled apart.

### Volume and surface area during distortion

The surface area of an RBC declines with age in the blood circulation^30,31^.

It is also known that an RBC's surface area cannot be increased by more than ~ 15% before it ruptures; this was discovered in studies with RBCs swelling in hypotonic media^32^. In our own experiments with RBCs suspended in gelatin gel that is then stretched, haemolysis is very extensive with two-fold stretching (ξ = 2) (unpublished results). Therefore, it was important to explore the volume and surface area inter-relationships that are brought about by the affine transformation [Eqs. (5) and (6)].

**Figure 5:**
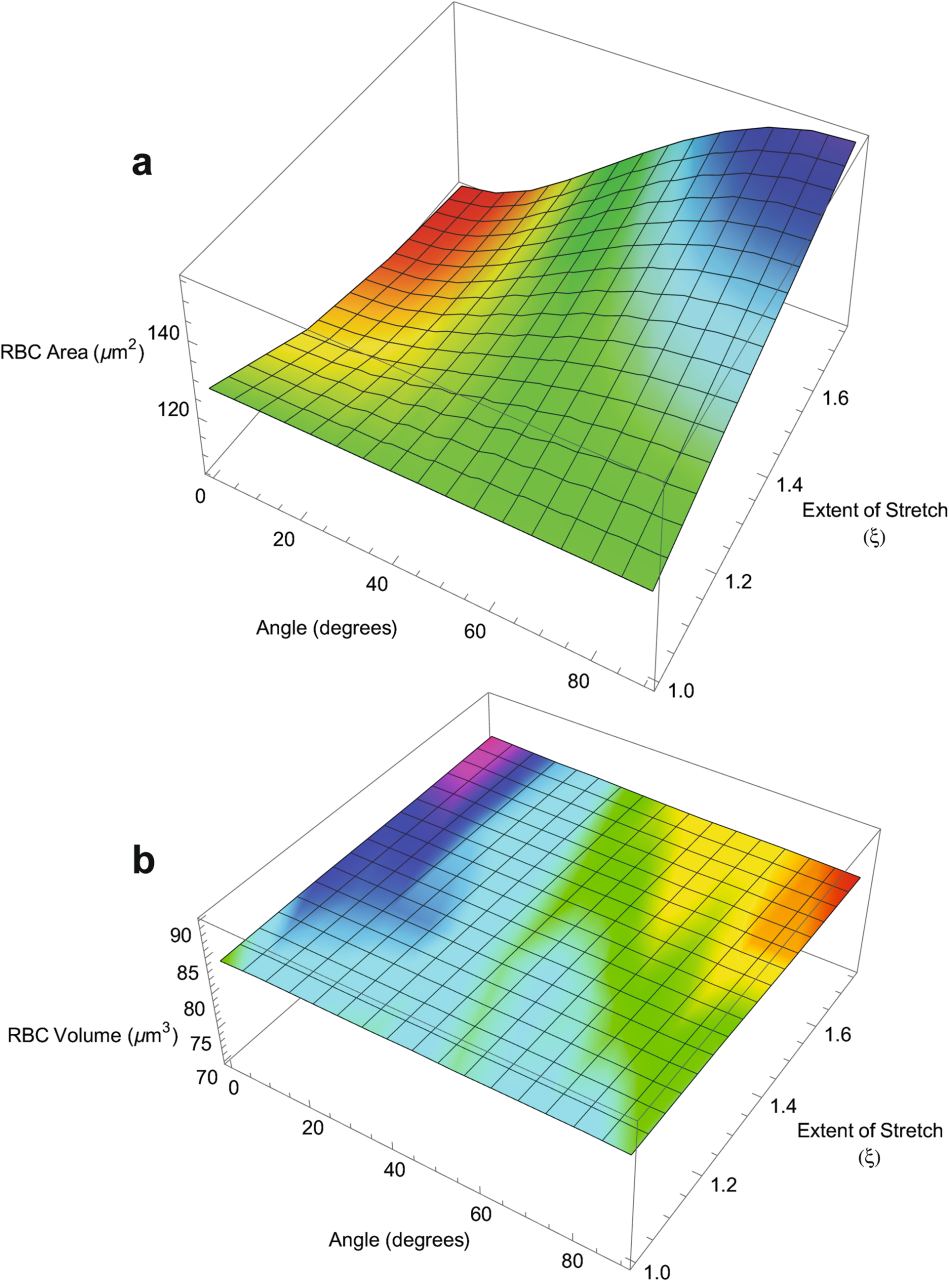
Dependence of RBC surface area (**a**) and volume (**b**) on rotation about to the *x*-axis (θ) and stretching along the *z*-axis (ξ). The relaxed RBC had the same dimensions as in Fig. 1.

Figure 5a shows that the surface area decreases on stretching if the RBC lies across the strain field; but it increases by up to 21% as the cell is stretched by 75% (ξ = 1.75) when aligned with its disc-plane parallel to the strain field. Meanwhile, the volume of the RBC scarcely changed under all the angles of orientation and extents of stretching explored herein (Fig. 5b).

Overall, we concluded from Fig. 5 that RBCs sustain increased *or* decreased surface area depending on their initial orientation in the gel on stretching the sample, while the volume did not change significantly. The extent of area change is much less than would occur with a sphere; and this helps explain why the biconcave disc shape has been naturally selected. Specifically, the particular shape enables volume and surface area preservation during passage of the RBC through the capillaries of the peripheral tissues and lungs^2,30,31,33^.

### Colour-coded curvatures for different values of θ and ξ

**Figure 6:**
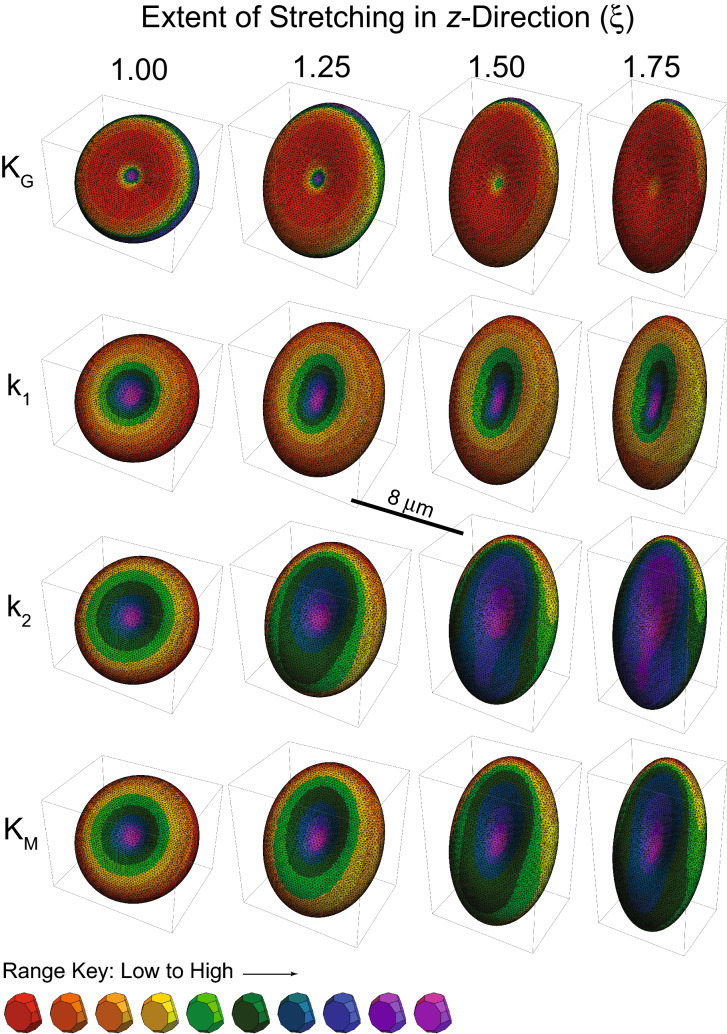
Curvatures of RBC rotated 45° around the *x*-axis and stretched in the *z*-direction by the extents (ξ) indicated above. The relaxed RBC had the same dimensions as in Fig. 1. The central scale bar indicates *d* = 8 μm, the main diameter of the fully relaxed RBC. K_G_ denotes Gaussian Curvature; k_1_, Principal Curvature (maximum); k_2_, Principal Curvature (minimum); and K_M_, Mean Curvature. The values of the respective curvatures across the 10 subdivisions of the domain of values are those shown in the central column of the polygonal graphs in Fig. 7.

Figure 6 shows RBCs that were tilted at 45° around the *x*-axis and progressively strained from no extension to a maximum of ξ = 1.75. The changes in curvature are indicated by the changes in colour; the most notable feature for the Gaussian curvature (K_G_) is the increasing dominance of areas of red denoting increased area of lower values as the RBCs are stretched. On the other hand, the intermediate values (green) dominate the area of the values of k_1_ (the maximum Principal Curvature). Numerous other comparisons can be made, as are taken up in the “Discussion”.

**Figure 7:**
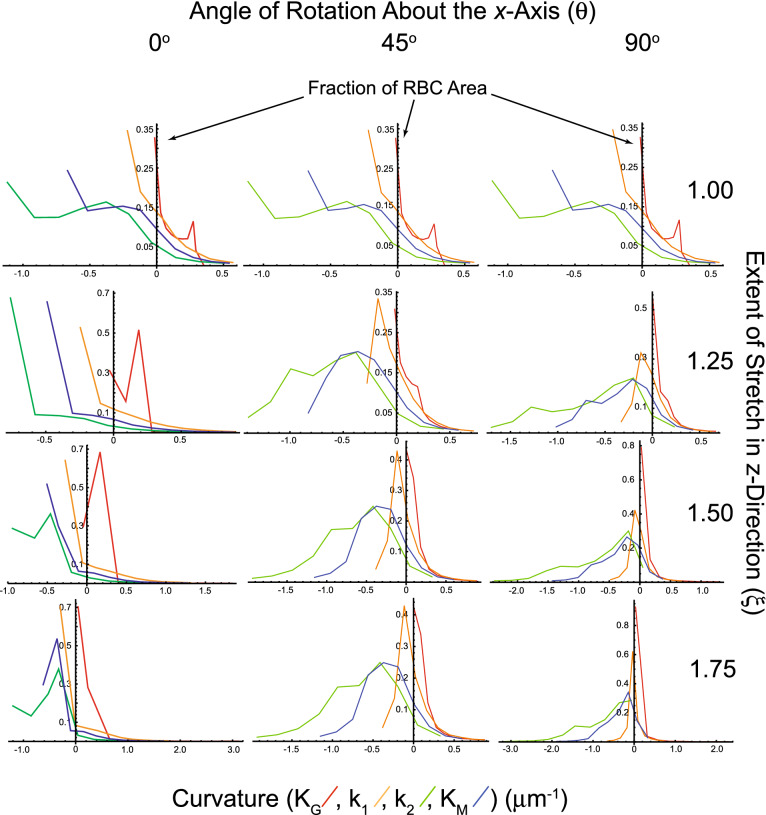
Curvature graphs of an RBC rotated at 0°, 45° and 90° around the *x*-axis and stretched in the *z*-direction by the extents (ξ) indicated on the right. The colours of the polygonal plots indicate: Gaussian Curvature K_G_ (μm^−2^), red; Principal Curvature (maximum), k_1_, orange; Principal Curvature (minimum), k_2_, green; and Mean Curvature K_M_, blue. Each discontinuity of the polygons denotes the mean value in curvature of the bin in the given curvature domain.

Another way of depicting the changes in curvature with orientation and strain is via a form of histogram shown in Fig. 7. The graphs show the fraction of the RBC area that is occupied by triangles with curvature (for each of the four types) in the neighbourhood of the mean values that correspond to 10 bins, arranged uniformly between the minimum and maximum values of the respective curvature.

The values of the curvatures span different ranges in all scenarios of orientation and strain. When there was no strain (top row of Fig. 7, and Fig. 3) the triangles with k_1_ (orange line) of smallest value occupied the largest area. When the RBCs at 0° were stretched the minimum of k_1_ decreased in value but occupied a much larger area of the cell as stretching was increased. Another notable feature at 45° orientation was the shift in k_2_ and the Mean Curvature (green and blue lines) to larger values as stretching was increased, with the maximum area occupied by triangles of intermediate values. Also, there is a clearly defined maximum value in these polygonal graphs. For the RBCs at 90° orientation k_1_ and k_2_ both decreasd with increased stretching, with the maximum area occupied by large values. Overall, the patterns of all four polygonal graphs shifted progressively as stretching was increased, but at each of the angles of orientation the patterns were distinctly different.

This is but a snapshot of RBCs orientated at three angles; for a continuous distribution of angles, we would expect a smooth transition from the left hand column of polygonal graphs through the middle column to the right hand column.

### Edge length distribution as a function of extent of stretching

**Figure 8:**
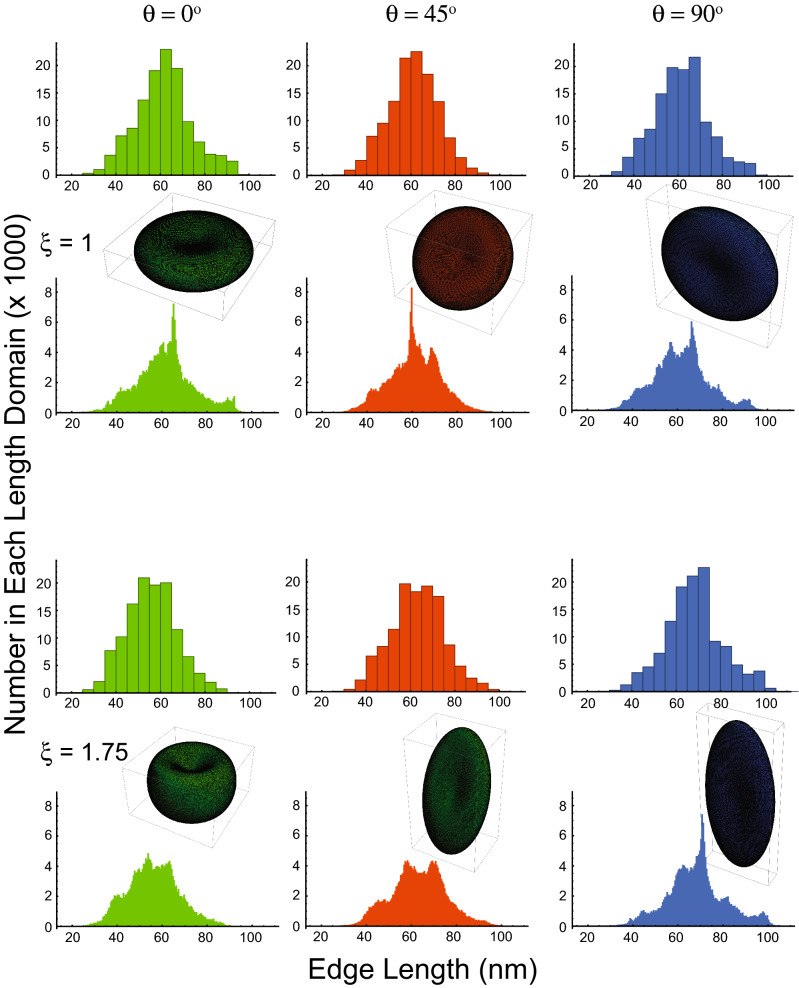
Histograms of the lengths of the 121,000 edges in the triangularization of an RBC at each of three rotations about the *x*-axis with no stretching (ξ = 1), and 75% stretching (ξ = 1.75). The colour coding was used to distinguish the three groups of data according to the rotations: green 0°, red 45°, and blue 90°. The bin numbers were 20 and 200 for the upper and lower pair of histograms at each value of stretching, ξ, respectively. The insets show the RBC shapes from which the histograms were made; they are the same as given in Figs. 4, S4 and S5.

Figure 8 shows that for θ = 0° the median value of edge length of the triangularization decreased on stretching by 75% more than the original value. This is consistent with the fact that Fig. 5 shows that for θ = 0° the surface area decreased with increasing ξ. The main feature for the RBCs at 45° is the emergence of a broad bimodal distribution of edge lengths that is most clearly evident in the bottom of the middle column of the histograms. On the other hand, when the RBCs were at 90°, the distribution of edge lengths remained relatively narrow for stretching all the way to ξ = 1.75. From Fig. 5, it is at this orientation that most increase in area took place, and it is especially clear in the bottom right-hand histogram that the median edge length was ~ 70 nm; this is similar to the second maximum in the second column. Consistent with this observation is that both RBC orientations displayed increases in surface area as well (Fig. 5).

## Discussion

### Triangularization

The fully extended α- and β-spectrin heterotetramer has an end-to-end length of ~ 200 nm^24^; but the filaments of unstretched RBC membranes have an average distance, between the nodal F-actin helices, of 60–70 nm^34^ and up to 85 nm^29^. The automatic triangularization that we used here to model the RBC cytoskeleton was controlled to correspond to 121,000 α- and β-spectrin heterotetramers; and this gave ~ 65 nm as the mean value of the distance between two neighbouring nodes. This would imply that native spectrin has its filaments in a bent or serpentine form that more than halves the end-to-end distance of the tetramer. This state of the struts could be maintained by the central complex coordinated by ankyrin-1^24^ imposing curvature on them.

Given that the fully extended struts of the cytoskeleton are ~ 200 nm long^24^, basic geometry (triangle side *a* gives area = *a*^2^ $\sqrt 3$/4) yields an estimate of the area of the resulting tessellation as follows: a single equilateral triangle of 200 nm on each side has an area of 0.0173 μm^2^. With a closed single-sheet surface like the RBC, each edge in the tessellation is shared with two triangles, and since there are three edges per triangle, the number of triangles is 2/3 the number of edges. This implies that there are 80,667 triangles, which, when multiplied by 0.0173 μm^2^, gives a total area of 1397 μm^2^, a number 10.9 times greater than for the real/actual RBC.

On the other hand, if we assume there are 80,667 cytoskeletal triangles, this implies a triangle area of 128/80,667 = 0.0016 μm^2^, which translates (using the triangle area formula above) to a side length of 60.8 nm. In other words, the internodal distance of the RBC cytoskeletal network should be ~ 60 nm. This number compares favourably with what we obtained as the mean distance of the edges in the tessellation analysed in Fig. 2: viz., 75% of the edges span 60–70 nm.

While the observation of a less-than-fully-extended triangular spectrin mesh could have been deduced without the complicated triangularization used here, the analysis nevertheless adds credibility to the ab initio triangularization process; while the discussion above confirms the consistency of the numerical values reported in the literature with a (fairly) regular triangular tessellation^24^.

### Curvature

In its present form, the analysis of curvature and its graphical representation conveys a semi-quantitative impression of how the distorted RBCs might transmit the locally altered shape of the membrane to Piezo1. However, the range-of-influence of membrane curvature on Piezo1 remains to be determined. Perhaps a finer mesh of triangularization is needed to explore this. The approaches adopted here should be extendable to such situations.

At the level of formal 3D differential geometry it was important to check for conformity of the total curvature with the Gauss–Bonnet Theorem^17^, which states that the integral of the Gaussian curvature over a closed surface will be 4π for surfaces like the RBC, even with its dimples that have regions of negative curvature. Notebook 5 shows an example of this outcome in which the Gaussian curvature in each triangle was multiplied by the area of the respective triangle and then the sum taken over them all. This is tantamount to a finite difference approximation to the surface integral and the result was a gratifying verification of the overall curvature analysis.

### Mesh-triangle area expansion and diminution

**Figure 9:**
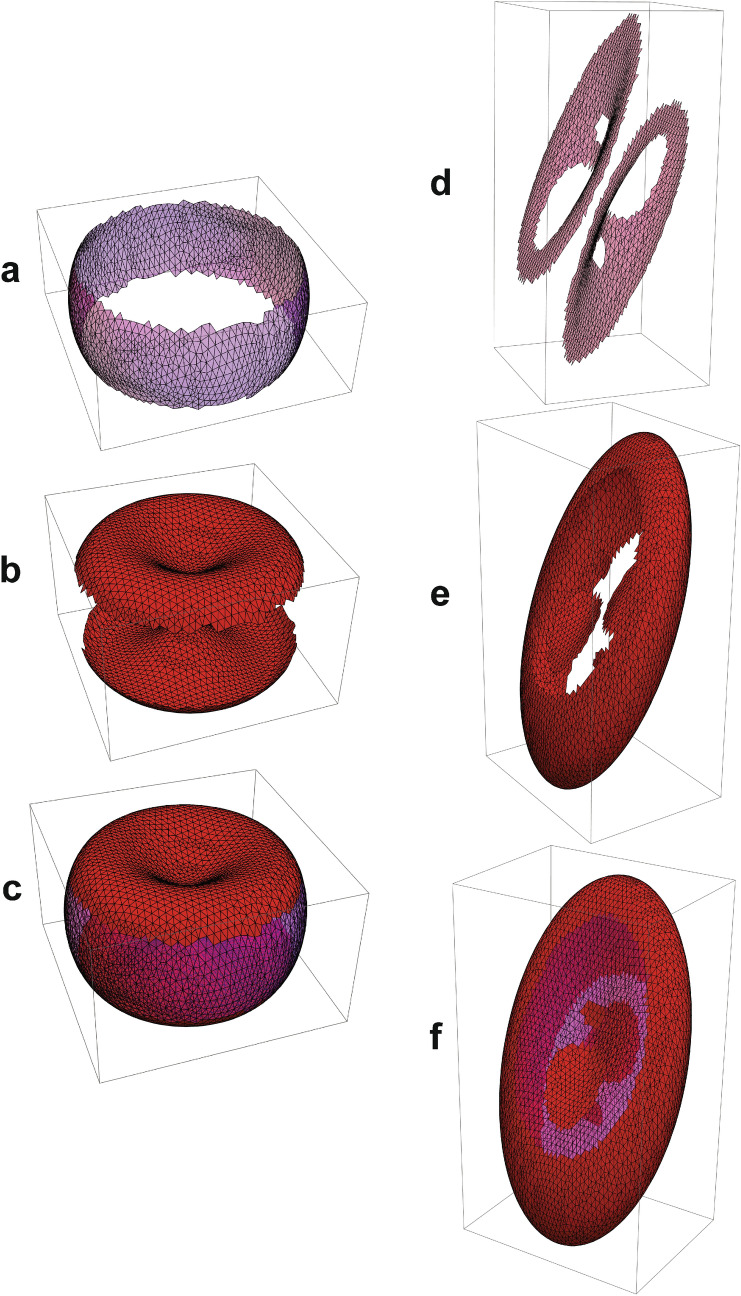
Partitioning of mesh-triangles of an RBC into those that underwent an increase in area and those that were diminished when the cell was strained in the z-direction. The RBCs had an angle of orientation to the strain field of θ = 0° and θ = 45°, respectively, prior to being stretched by the factor ξ = 1.75. In order to speed up computation the triangularization was performed with 13,188 edges and not the real ~ 121,000. The *Mathematica* Notebook was written to achieve direct correspondence between a triangle in the relaxed RBC and its counterpart in the stretched state. (**a**) Shows the mesh-triangles that were increased in area; (**b**) those that were diminished in area; and (**c**) is a union of (**a**) and (**b**), which shows complete closure of the mesh by the two classes of triangles. For the θ = 45° orientation, (**d**) shows the mesh-triangles that were increased in area. Note how these occupy two disconnected manifolds, while (**e**) is the single perforated surface that contains those mesh-triangles that were diminished in area; and (**f**) is the union of the two classes of triangles. Notebook 7 contains the computations not only for this figure but for mapping the differences in the four curvatures between the relaxed and stretched RBCs.

Figure 9a–c are examples of the partitioning of mesh-triangles into those that are expanded and those that are diminished when the RBC is exposed to the linear strain field at 0°. On the other hand, with the RBC at 45° (Figs. 9d–f) the pattern of the mesh-triangles that are expanded or diminished is quite different. In the former case, the expanded triangles are arrayed in a single ring, while in the latter case, there are two separate regions each with two holes in them. For the diminished triangles in the RBC at 0° (Fig. 9b), there are two separate continuous zones; and for the RBC at 45°, there is one centrally perforated sheet. If there were an uneven distribution of Piezo1 throughout the RBC membrane, say in the dimples as has been suggested by Svetina et al.^35^, then the action of Piezo1 would depend on the orientation of the RBCs to the strain field. Hence, it is not obvious whether the net effect of distortion of a randomly orientated population of such RBCs would actually lead to net activation of Piezo1.

Numerous studies have shown that Piezo1 responds to an increase in membrane tension^7,36,37^. Ion channel reconstitution into lipid bilayers has revealed that many eukaryotic channels respond to membrane tension^38^. The idea that ion channels can respond to membrane tension originates from the pioneering studies of bacterial mechanosensitive channels^39,40^; and this has been underscored recently by work using a membrane-tension device with KcsA, that had not previously been considered to be mechanosensitive^41^. Hence, it is conceivable that membrane transporters other than Piezo1 in RBCs could be affected by shape changes of the type modelled here.

It is proposed that expansion of a mesh-triangle corresponds to an increase in local tension, whereas a decrease in area corresponds to relaxation. Therefore, from the numerous studies mentioned above, we conclude that even if Piezo1 were uniformly distributed around the RBC membrane, then members of the channel population would not be uniformly activated (at a given orientation to the direction of the strain field). This is further complicated by the non-uniform local levels of cytoskeletal contractility in RBCs, which will also influence local membrane tension^42^. Thus, the net extent of activation of the Piezo1 channels would depend on the orientation of each RBC to the strain field, and to local levels of RBC contractility. This would make the idea of segregation of Piezo1 to the pits of the RBC dimples less plausible^35^. Of course, studies involving light microscopy used with fluorescent antibodies directed specifically at Piezo1 on RBCs could settle this question.

### Cytoskeletal rearrangement

Since our pictures of RBCs, in various states of distortion are static, we can deduce nothing about the kinetics of cytoskeletal rearrangement of a kind suggested by Gov et al.^43^. However, the images of positive and negative changes in area of mesh-triangles (Fig. 9) suggest that rearrangements of the struts of the geodetic dome would occur such that strain would be re-distributed more uniformly. Whether this rearrangement occurs on the timescale of the blood circulation, or even during the ~ 0.3 s taken to pass through a capillary in the human body^44^, requires more experimentation.

## Conclusions

There are many aspects of RBC shape, volume and flexibility that are ripe for explanation^45,46^, and inevitably these studies will tap into recent findings on Piezo1, and new analytical methods including computational fluid dynamics.

At a deeper conceptual level, the work described here is an exploration of the geometrical constraints on 'biological form' (e.g.,^28,47^); in this case, the particular shapes are those taken up by an RBC in a strain field. The linear strain field used here is the simplest of all, being in a single direction: but it is consistent with that imposed by a stretched gel, as used in our NMR experiments^15^. Much more complicated deformations occur in flowing systems, in which some domains of the RBC membrane are stressed into more positive curvature, while others simultaneously undergo more negative curvature^48–51^. The ability of the RBC to accommodate these contortions decreases with the age of the cell and it is posited as a major factor in what determines RBC survival, for ~ 120 days in the circulation^33^. Transient, distorted shapes exist in RBCs when they are in regions of high velocity that impose non-laminar flow around prosthetic or even healthy heart valves. Flow changes occur during valve development in cardio-genesis in particular, and flow is modified around calcified or diseased valves, not just prosthetic ones^50,51^; so, there is considerable merit in having a computationally accessible means of modelling RBC shape changes by using the methods presented here.

The RBC shapes in various in vivo situations have begun to yield to computation. For example, it is known that the stresses on an RBC can be so extreme around prostheses as to lead to cell rupture. To date, simulating such outcomes really only yield to advanced supercomputing e.g.,^52^.

The other critical aspect of simulations for surface deformation is the distance scale of the deformations that are required to activate mechanosensitive ion channels like Piezo1^11,14^. The changes in curvature invoked at the tip of a patch-clamp pipette are quite extreme, across a diameter of ~ 1 μm or an area of 0.79 μm^2^, implying that 80,667(number of triangles) × 0.79 (μm^2^, area of patch)/128 (μm^2^, total area of RBC) ≅ 500 cytoskeletal triangles are spanned. The resolution of the present simulations and the curvature near the rim of the RBC are in the same range. Therefore, if increased membrane bending activates Piezo1, then it will be those molecules at the rim of the cells (as in Fig. 6, right) that would be activated. On the other hand, if a decrease in curvature (increased area of flatness) is what activates them, then it will be also found in the stretched cell. Even more interesting is the fact that the two Principal Curvatures k_1_ and k_2_ *both* increase on stretching the cell (Fig. 6 right).

However (as alluded to in the “Introduction”), in patch clamping experiments the visible curvature appears *not* to be what activates the channel. The inflation of the membrane dome is driven by the confinement by the micro-pipette that is on the micron scale. It is the tension (and presumably flattening at the nm scale below the resolution of a confocal microscope) that appears to drive the channels to open^14,37,53–55^. Whereas in stretched/compressed gel experiments, the morphological forms taken up by the RBCs will be like those shown in this article. However, more curvature of the membrane on the length scales seen in membrane flickering^56,57^ would be superimposed on these shapes.

Future directions for this computational work will involve larger scale simulations of population-averaged curvatures in cells under strain, and in strain fields that are not simply unidirectional and linear. Then, correlations might be able to be made with experimental measurements like those already reported on stretched/compressed gels^1,2^, including estimates of membrane tension during electrophysiological measurements performed on whole RBCs.

## Supplementary Information

Supplementary Information 1.

Supplementary Information 2.

Supplementary Information 3.

Supplementary Information 4.

Supplementary Information 5.

Supplementary Information 6.

Supplementary Information 7.

Supplementary Information 8.

## Abbreviations

3D: 3-Dimensional
NMR: Nuclear magnetic resonance
RBC: Red blood cell
