# Supplementary material for: Surface model of the human red blood cell simulating changes in membrane curvature under strain
Source: Sci Rep. 2021 Jul 1;11:13712. doi: 10.1038/s41598-021-92699-7 (PMC8249411; doi:10.1038/s41598-021-92699-7)
Supplement: Supplementary file 1 — Supplementary Information 1. [file 41598_2021_92699_MOESM1_ESM.pdf]

SUPPLEMENTARY INFORMATION for:
Surface model of the human red blood cell simulating changes in membrane curvature under strain

Philip W. Kuchel[a*], Charles D. Cox[b,c], Daniel Daners[d], Dmitry Shishmarev[e], and Petrik Galvosas[f]

[a]*School of Life and Environmental Sciences, University of Sydney, Sydney, NSW, Australia.*

[b]*Victor Chang Cardiac Research Institute, Darlinghurst, Sydney, NSW, Australia.*

[c]*St Vincent's Clinical School, Faculty of Medicine, University of New South Wales, Sydney, New South Wales, Australia.*

[d]*School of Mathematics and Statistics, University of Sydney, Sydney, NSW, Australia.*

[e]*John Curtin School of Medical Research, Australian National University, Canberra, ACT, Australia.*

[f]*MacDiamid Institute for Advanced Materials and Nanotechnology, School of Chemical and Physical Sciences, Victoria University Wellington, Wellington, New Zealand.*

Supplementary Introduction

General. Here we provide additional context and simulations in our studies of the changes in curvature that RBCs undergo when they express enhanced glycolytic and transmembrane cation flux in stretched or compressed gels [1, 2]. The formal theory of curvature is dealt with comprehensively in [3], but we provide an overview of it to explain the analyses that were used in this study, especially as it relates to Eq. 1, which we chose to describes the surface of the RBC [4].

Classical curvatures in three dimensions (3D). That more than one value of curvature is required to describe the bending of a Euclidean sheet in 3D is seen by inspecting surfaces like the hyperbolic paraboloid. It appears to bend in two (or more?) directions from a given point. Beginning with Figure S1**a** we have the purple hyperbolic paraboloid (also called a saddle). It has the Cartesian form $a\,x^2 - y^2 - z^2 = 0$ but is more conveniently represented in the parametric form $(v^2, u, -u^2 + a\,v^2)$ when drawing it. Figure S1**a** was generated with $a = 0.3$. The tangent plane is (v, u, 0) (Figure 1**b**), followed by the green tangent parabolic sheet (Figure S1**c**) and

the central parabola. The same reasoning was used to generate the pink tangent parabolic sheet underneath the purple hyperbolic paraboloid. The main pedagogic point is that there are two tangent sheets that meet at the saddle point and that they have oppositely signed values of different radii of curvature. This simple example highlights the requirement to consider two Principal Curvatures (k_1 and k_2) and those derived from them, Gaussian Curvature, $k_1 \times k_2$, and the Mean Curvature that is the average, $(k_1 + k_2)/2$.

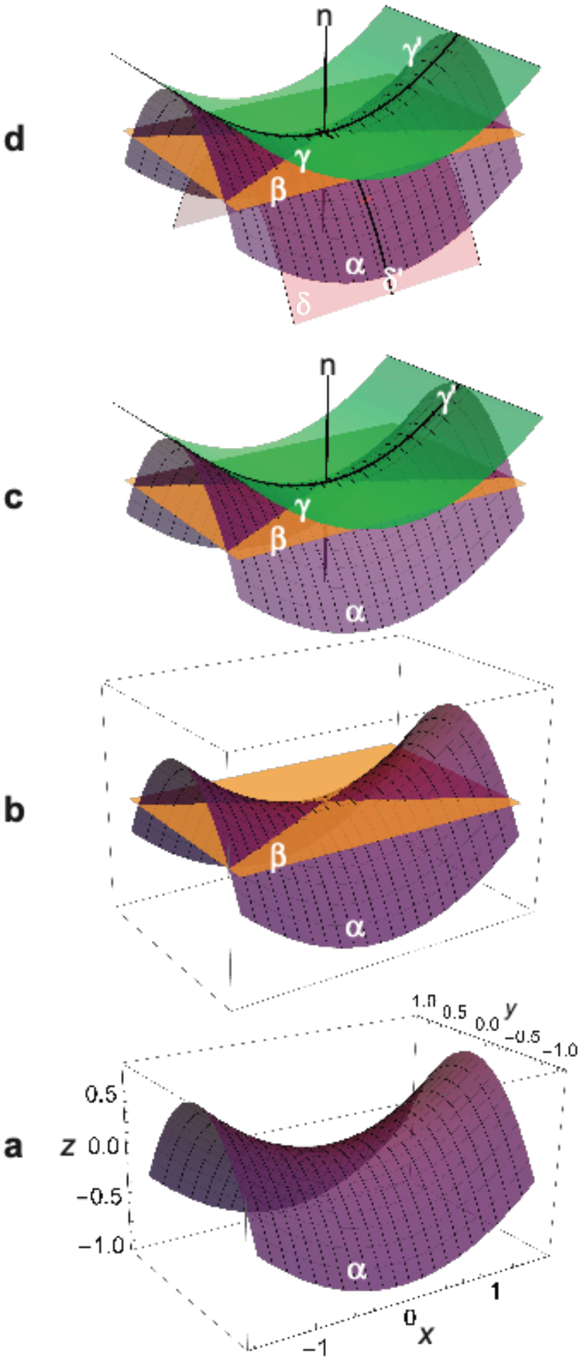

Figure S1. Hyperbolic paraboloid, a 3D surface with curvatures of opposite signs at a saddle point showing the conceptual build-up to two tangent sheets of different curvature at the saddle point. (**a**) Hyperbolic paraboloidal surface (sheet), α; (**b**) unique tangent plane to α at the 'saddle point', β; (**c**) sheet γ, tangent to α along the curve γ', with the normal vector n through the saddle point; and (**d**) sheet δ, tangent to α along the curve δ'. Notebook 1 has the *Mathematica* script used to generate this graphic.

The relevance of each of the four curvatures to understanding likely effects on RBCs of changes in their shape, which are mediated by proteins like Piezo1, are considered in the main Discussion.

Supplementary Results

Symbolic expression for Gaussian Curvature of an RBC at any angle of its main axis of symmetry to a uniform-strain field. It is hard to imagine that this complicated expression would have been able to be derived without using symbolic computation. Conversely, the merit of generating such an expression might be questioned. The reply is that it allows us to see, almost at a glance, the likely sensitivity of the overall expression to perturbations in particular parameter values. For example, the dependence on higher degrees of the strain factor, or the dependence on shape parameters pP, qQ, and rR, etc., and of course, the expressions provide accurate numerical computation of the curvatures.

$$
\begin{aligned}
&\kappa\mathrm{GFunc}[x\_,\ y\_,\ z\_] := \\
&\Big(8\left(2 z^2+\xi^2\left(pP+2\left(x^2+y^2\right)\xi\right)\right) \\
&\quad\Big(16 z^8+12\, pP\, z^6 \xi^2+12\, qQ\, z^6 \xi^2+64 x^2 z^6 \xi^3+64 y^2 z^6 \xi^3+3\, pP^2 z^4 \xi^4+6\, pP\, qQ\, z^4 \xi^4+3\, qQ^2 z^4 \xi^4+40\, pP\, x^2 z^4 \xi^5+ \\
&\quad 32\, qQ\, x^2 z^4 \xi^5+36\, pP\, y^2 z^4 \xi^5+36\, qQ\, y^2 z^4 \xi^5+pP^2\, qQ\, z^2 \xi^6+pP\, qQ^2 z^2 \xi^6+96 x^4 z^4 \xi^6+192 x^2 y^2 z^4 \xi^6+96 y^4 z^4 \xi^6+ \\
&\quad 10\, pP^2 x^2 z^2 \xi^7+8\, pP\, qQ\, x^2 z^2 \xi^7+6\, qQ^2 x^2 z^2 \xi^7+6\, pP^2 y^2 z^2 \xi^7+12\, pP\, qQ\, y^2 z^2 \xi^7+6\, qQ^2 y^2 z^2 \xi^7+44\, pP\, x^4 z^2 \xi^8+ \\
&\quad 28\, qQ\, x^4 z^2 \xi^8+80\, pP\, x^2 y^2 z^2 \xi^8+64\, qQ\, x^2 y^2 z^2 \xi^8+36\, pP\, y^4 z^2 \xi^8+36\, qQ\, y^4 z^2 \xi^8+2\, pP^2\, qQ\, x^2 \xi^9+pP^2\, qQ\, y^2 \xi^9+ \\
&\quad pP\, qQ^2 y^2 \xi^9+64 x^6 z^2 \xi^9+192 x^4 y^2 z^2 \xi^9+192 x^2 y^4 z^2 \xi^9+64 y^6 z^2 \xi^9+4\, pP^2 x^4 \xi^{10}+8\, pP\, qQ\, x^4 \xi^{10}+10\, pP^2 x^2 y^2 \xi^{10}+ \\
&\quad 8\, pP\, qQ\, x^2 y^2 \xi^{10}+6\, qQ^2 x^2 y^2 \xi^{10}+3\, pP^2 y^4 \xi^{10}+6\, pP\, qQ\, y^4 \xi^{10}+3\, qQ^2 y^4 \xi^{10}+16\, pP\, x^6 \xi^{11}+8\, qQ\, x^6 \xi^{11}+44\, pP\, x^4 y^2 \xi^{11}+ \\
&\quad 28\, qQ\, x^4 y^2 \xi^{11}+40\, pP\, x^2 y^4 \xi^{11}+32\, qQ\, x^2 y^4 \xi^{11}+12\, pP\, y^6 \xi^{11}+12\, qQ\, y^6 \xi^{11}+16 x^8 \xi^{12}+64 x^6 y^2 \xi^{12}+96 x^4 y^4 \xi^{12}+ \\
&\quad 64 x^2 y^6 \xi^{12}+16 y^8 \xi^{12}+(pP-qQ)\,\xi^2\left(-z^2+y^2 \xi^3\right) \\
&\quad\left(4 z^4+2 z^2 \xi^2\left(pP+qQ+4\left(x^2+y^2\right)\xi\right)+\xi^4\left(pP\left(qQ+2\left(-x^2+y^2\right)\xi\right)+2\xi\left(qQ\left(3 x^2+y^2\right)+2\left(x^2+y^2\right)^2\xi\right)\right)\right)\mathrm{Cos}[2\theta]- \\
&\quad (pP-qQ)^2\xi^4\left(z^4-6 y^2 z^2 \xi^3+y^4 \xi^6\right)\mathrm{Cos}[4\theta]+8\, pP\, y z^5 \xi^{7/2}\,\mathrm{Sin}[2\theta]-8\, qQ\, y z^5 \xi^{7/2}\,\mathrm{Sin}[2\theta]+4\, pP^2 y z^3 \xi^{11/2}\,\mathrm{Sin}[2\theta]- \\
&\quad 4\, qQ^2 y z^3 \xi^{11/2}\,\mathrm{Sin}[2\theta]+16\, pP\, x^2 y z^3 \xi^{13/2}\,\mathrm{Sin}[2\theta]-16\, qQ\, x^2 y z^3 \xi^{13/2}\,\mathrm{Sin}[2\theta]+16\, pP\, y^3 z^3 \xi^{13/2}\,\mathrm{Sin}[2\theta]- \\
&\quad 16\, qQ\, y^3 z^3 \xi^{13/2}\,\mathrm{Sin}[2\theta]+2\, pP^2\, qQ\, y z\, \xi^{15/2}\,\mathrm{Sin}[2\theta]-2\, pP\, qQ^2 y z\, \xi^{15/2}\,\mathrm{Sin}[2\theta]-4\, pP^2 x^2 y z\, \xi^{17/2}\,\mathrm{Sin}[2\theta]+ \\
&\quad 16\, pP\, qQ\, x^2 y z\, \xi^{17/2}\,\mathrm{Sin}[2\theta]-12\, qQ^2 x^2 y z\, \xi^{17/2}\,\mathrm{Sin}[2\theta]+4\, pP^2 y^3 z\, \xi^{17/2}\,\mathrm{Sin}[2\theta]-4\, qQ^2 y^3 z\, \xi^{17/2}\,\mathrm{Sin}[2\theta]+ \\
&\quad 8\, pP\, x^4 y z\, \xi^{19/2}\,\mathrm{Sin}[2\theta]-8\, qQ\, x^4 y z\, \xi^{19/2}\,\mathrm{Sin}[2\theta]+16\, pP\, x^2 y^3 z\, \xi^{19/2}\,\mathrm{Sin}[2\theta]-16\, qQ\, x^2 y^3 z\, \xi^{19/2}\,\mathrm{Sin}[2\theta]+ \\
&\quad 8\, pP\, y^5 z\, \xi^{19/2}\,\mathrm{Sin}[2\theta]-8\, qQ\, y^5 z\, \xi^{19/2}\,\mathrm{Sin}[2\theta]+4\, pP^2 y z^3 \xi^{11/2}\,\mathrm{Sin}[4\theta]-8\, pP\, qQ\, y z^3 \xi^{11/2}\,\mathrm{Sin}[4\theta]+ \\
&\quad 4\, qQ^2 y z^3 \xi^{11/2}\,\mathrm{Sin}[4\theta]-4\, pP^2 y^3 z\, \xi^{17/2}\,\mathrm{Sin}[4\theta]+8\, pP\, qQ\, y^3 z\, \xi^{17/2}\,\mathrm{Sin}[4\theta]-4\, qQ^2 y^3 z\, \xi^{17/2}\,\mathrm{Sin}[4\theta]\Big)\Big)\Big/ \\
&\Big(\xi^{10} \\
&\quad\Big(\mathrm{Abs}\left[\frac{4 x z^2}{\xi}+2 x \xi\left(pP+2\left(x^2+y^2\right)\xi\right)\right]^2+\mathrm{Abs}\left[\frac{4 y z^2}{\xi}+y\xi\left(pP+qQ+4\left(x^2+y^2\right)\xi\right)+(pP-qQ)\, y\xi\,\mathrm{Cos}[2\theta]+\frac{(pP-qQ)\, z\,\mathrm{Sin}[2\theta]}{\sqrt{\xi}}\right]^2+ \\
&\qquad \mathrm{Abs}\left[\frac{4 z^3+z\xi^2\left(pP+qQ+4\left(x^2+y^2\right)\xi\right)-(pP-qQ)\, z\xi^2\,\mathrm{Cos}[2\theta]+(pP-qQ)\, y\,\xi^{7/2}\,\mathrm{Sin}[2\theta]}{\xi^4}\right]^2\Big)^2\Big)
\end{aligned}
$$

. (S1)

Symbolic expression for Mean Curvature of an RBC at any angle, then exposed to a uniform strain field.

```
xMFunc[x_, y_, z_] :=
 (-ξ^8 (Abs[4 x z^2/ξ + 2 x ξ (pP + 2 (x^2 + y^2) ξ)]^2 + Abs[4 y z^2/ξ + y ξ (pP + qQ + 4 (x^2 + y^2) ξ) + (pP - qQ) y ξ Cos[2 θ] + ((pP - qQ) z Sin[2 θ])/Sqrt[ξ]]^2 +
      Abs[(4 z^3 + z ξ^2 (pP + qQ + 4 (x^2 + y^2) ξ) - (pP - qQ) z ξ^2 Cos[2 θ] + (pP - qQ) y ξ^(7/2) Sin[2 θ])/ξ^4]^2)
    (4 z^2 (3 + 2 ξ^3) + ξ^2 (pP + 3 pP ξ^3 + qQ (1 + ξ^3) + 4 (x^2 + y^2) ξ (1 + 4 ξ^3)) + (pP - qQ) ξ^2 (-1 + ξ^3) Cos[2 θ]) +
   8 x^2 ξ^6 (2 z^2 + ξ^2 (pP + 2 (x^2 + y^2) ξ))
    (8 z^4 + 2 pP z^2 ξ^2 + 2 qQ z^2 ξ^2 + 8 x^2 z^2 ξ^3 + 8 y^2 z^2 ξ^3 + 4 z^4 ξ^3 + 4 pP z^2 ξ^5 + 16 x^2 z^2 ξ^6 + 16 y^2 z^2 ξ^6 + pP^2 ξ^7 + 8 pP x^2 ξ^8 +
      6 pP y^2 ξ^8 + 2 qQ y^2 ξ^8 + 12 x^4 ξ^9 + 24 x^2 y^2 ξ^9 + 12 y^4 ξ^9 + 2 (pP - qQ) ξ^2 (-z^2 + y^2 ξ^6) Cos[2 θ] + 2 (pP - qQ) y z ξ^(7/2) (1 + ξ^3) Sin[2 θ]) +
   (4 z^3 + z ξ^2 (pP + qQ + 4 (x^2 + y^2) ξ) - (pP - qQ) z ξ^2 Cos[2 θ] + (pP - qQ) y ξ^(7/2) Sin[2 θ])
    (16 x^2 z ξ^6 (2 z^2 + ξ^2 (pP + 2 (x^2 + y^2) ξ)) + ξ^6 (8 y z + (pP - qQ) Sqrt[ξ] Sin[2 θ])
       (4 y z^2 + y ξ^2 (pP + qQ + 4 (x^2 + y^2) ξ) + (pP - qQ) y ξ^2 Cos[2 θ] + (pP - qQ) z Sqrt[ξ] Sin[2 θ]) +
      (12 z^2 + ξ^2 (pP + qQ + 4 (x^2 + y^2) ξ) - (pP - qQ) ξ^2 Cos[2 θ])
       (4 z^3 + z ξ^2 (pP + qQ + 4 (x^2 + y^2) ξ) - (pP - qQ) z ξ^2 Cos[2 θ] + (pP - qQ) y ξ^(7/2) Sin[2 θ])) +
   ξ^6 (4 y z^2 + y ξ^2 (pP + qQ + 4 (x^2 + y^2) ξ) + (pP - qQ) y ξ^2 Cos[2 θ] + (pP - qQ) z Sqrt[ξ] Sin[2 θ])
    (16 x^2 y ξ^6 (2 z^2 + ξ^2 (pP + 2 (x^2 + y^2) ξ)) + ξ^3 (4 z^2 + ξ^2 (pP + qQ + 4 (x^2 + 3 y^2) ξ) + (pP - qQ) ξ^2 Cos[2 θ])
       (4 y z^2 + y ξ^2 (pP + qQ + 4 (x^2 + y^2) ξ) + (pP - qQ) y ξ^2 Cos[2 θ] + (pP - qQ) z Sqrt[ξ] Sin[2 θ]) +
      (8 y z + (pP - qQ) Sqrt[ξ] Sin[2 θ]) (4 z^3 + z ξ^2 (pP + qQ + 4 (x^2 + y^2) ξ) - (pP - qQ) z ξ^2 Cos[2 θ] + (pP - qQ) y ξ^(7/2) Sin[2 θ]))) /
 (2 ξ^12
   (Abs[4 x z^2/ξ + 2 x ξ (pP + 2 (x^2 + y^2) ξ)]^2 + Abs[4 y z^2/ξ + y ξ (pP + qQ + 4 (x^2 + y^2) ξ) + (pP - qQ) y ξ Cos[2 θ] + ((pP - qQ) z Sin[2 θ])/Sqrt[ξ]]^2 +
     Abs[(4 z^3 + z ξ^2 (pP + qQ + 4 (x^2 + y^2) ξ) - (pP - qQ) z ξ^2 Cos[2 θ] + (pP - qQ) y ξ^(7/2) Sin[2 θ])/ξ^4]^2)^(3/2))
```

. (S2)

Triangularization. The partitioning of the model RBC surface into triangles was carried out automatically in *Mathematica* [5] as described in Theory of Methods. In a strictly geometrical sense, the area of the triangles cannot all be the same and yet it was important to gain a sense of what values these spanned. It is a simple matter (in *Mathematica*) to sort the triangle areas into batches (bins) according to a specified mean value. We chose 10 bins, and by colour-coding the triangles of different sizes it was possible to draw them on the surface. Figure S1**a** shows that the colouring is dominated by green triangles, which correspond to areas in the neighbourhood of 1700 nm^2.

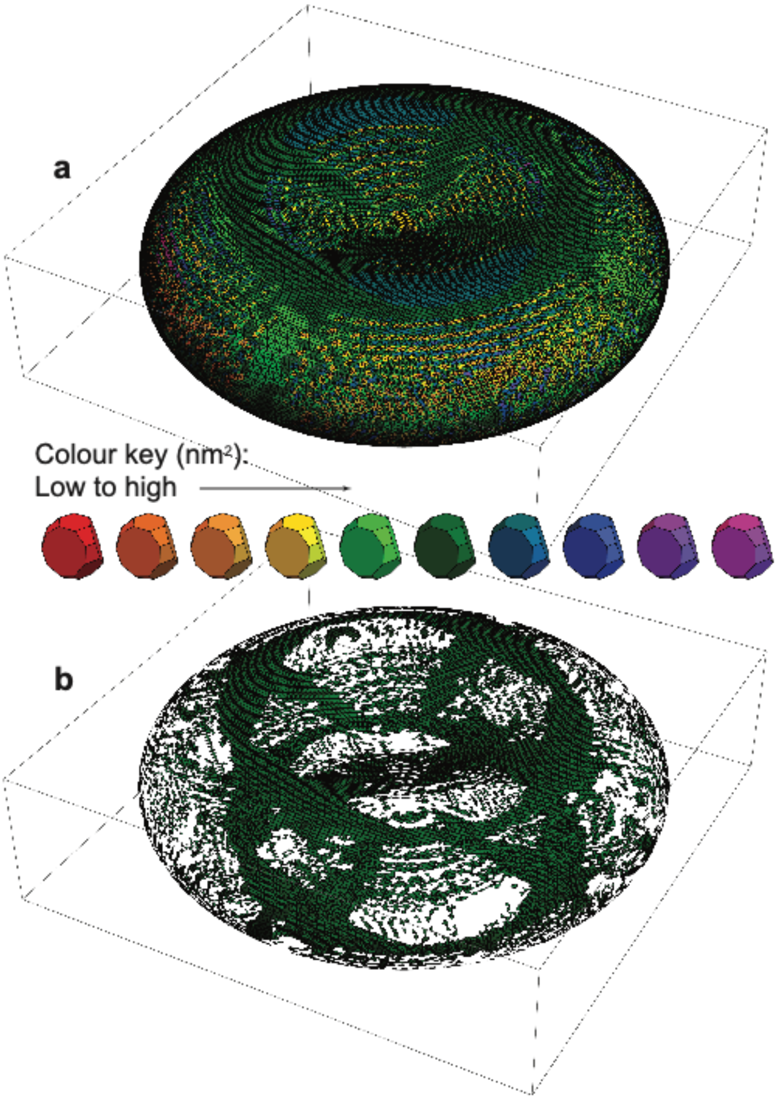

Figure S2. RBC surface described by Eq. 1 with dimensions as used in Figure 1 (d = 8 μm, b = 1 μm, and h = 2.12 μm). **(a)** The RBC was partitioned into 81,264 triangles, with 40,634 edges, and 121,896 edges (struts), as described in Theory of Methods. **(b)** The triangle areas were sorted into 10 domains of equal span, from a minimum of 0.0094 to a maximum of 3431 nm^2. Shown here are the 27541 triangles in the 6th bin, with a mean area of 1873 nm^2. Over the whole RBC the average triangle area was 1575 nm^2. The colour coding came out to be centred on the two shades of green.

The other way of conveying a sense of triangle-size distribution is by directly applying *Mathematica's* Histogram function to the list of areas. The result of doing this for the triangles shown in Figure S2 is shown here:

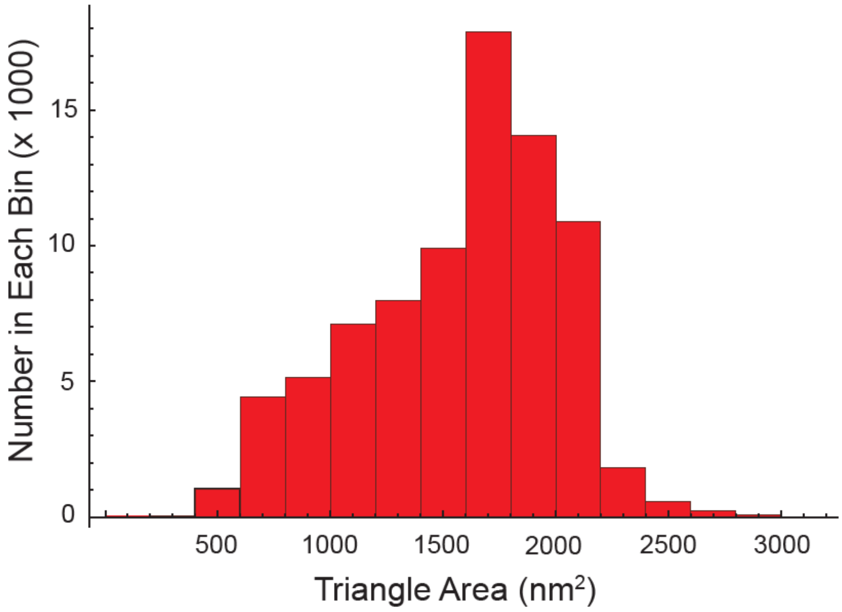

Figure S3. Histogram of the areas of the triangles shown in Figure S2.

It is evident that the majority of the triangle areas lie in a partitioning around ~1700 nm^2. There were 81,264 triangles covering the whole area of 128 μm^2 so the average value was 1575 nm^2. This smaller value than the median is consistent with the skewness that is evident in the histogram. The asymmetry was further exposed by the graphic of the second partitioning (not shown here but in Notebook 2) in which the small triangles lie around the rim of the RBC. Whether this skewed distribution of mesh-triangle sizes occurs in a real RBC is unknown. It would only be expected if the algorithm that performs the triangularization in *Mathematica* relies on calculating local curvature in a way that assembly of the real cytoskeleton might also, in effect, do.

Rotation and elongation. Figures S4 and S5 show the results of triangularization of the RBC surface, with the cells orientated at 0° and 90° in the strain field. The *Mathematica* script was the same as that used for Figure 4.

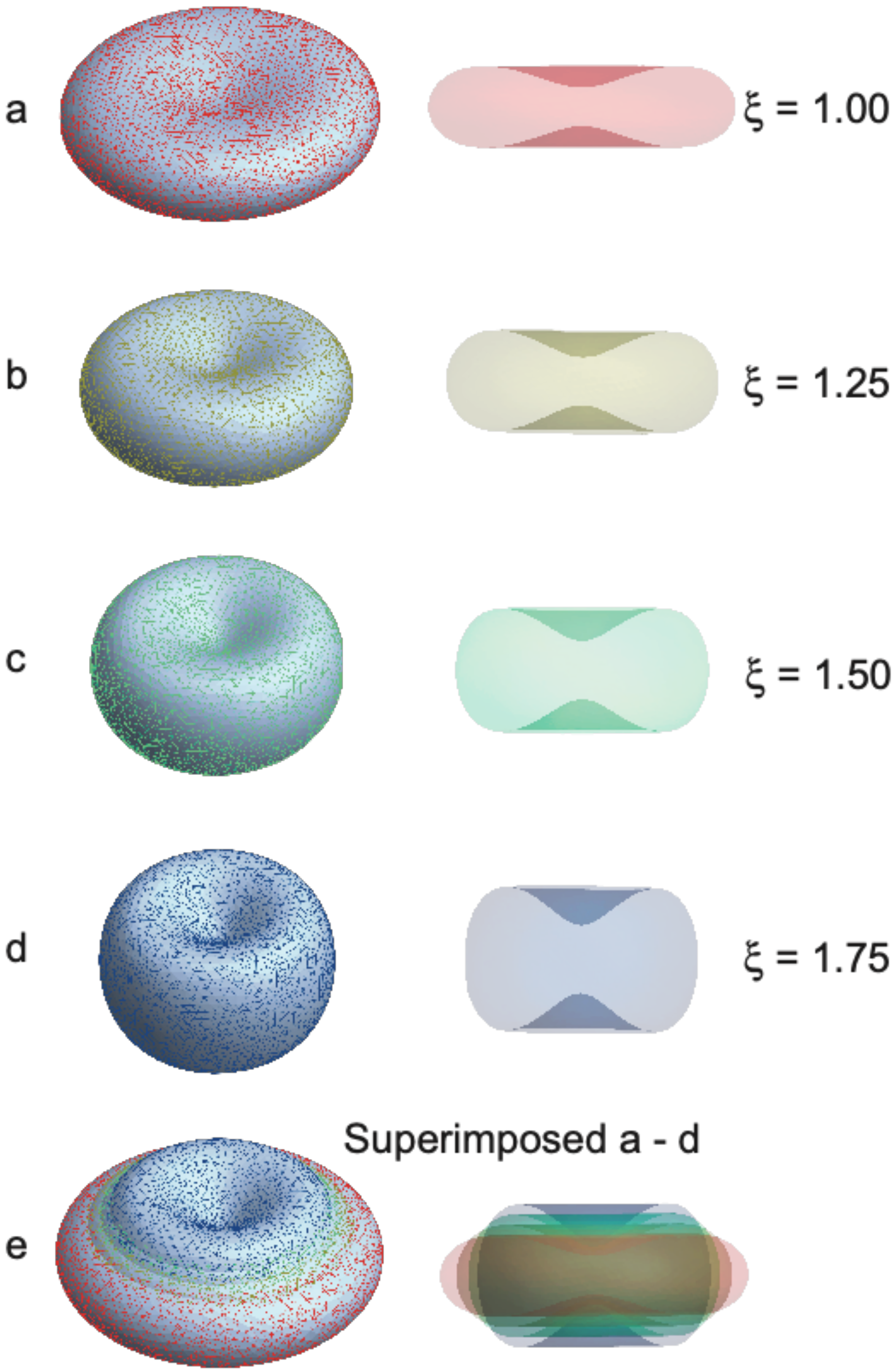

Figure S4. RBCs lying in the *x,y*-plane with a linear strain field applied in the *z*-direction. The undistorted RBC had the same dimensions as in Figure 1. RBCs (left) and their cross-sections (right): (**a**) undistorted RBC, $\xi = 1$;

(**b**) elongated 25%, ξ = 1.25; (**c**) elongated 50%, ξ = 1.5; (**d**) elongated 75%, ξ = 1.25; and (**e**) superimposed images to convey the relative dimensions. Colour coding was used to distinguish between the RBCs in **e**.

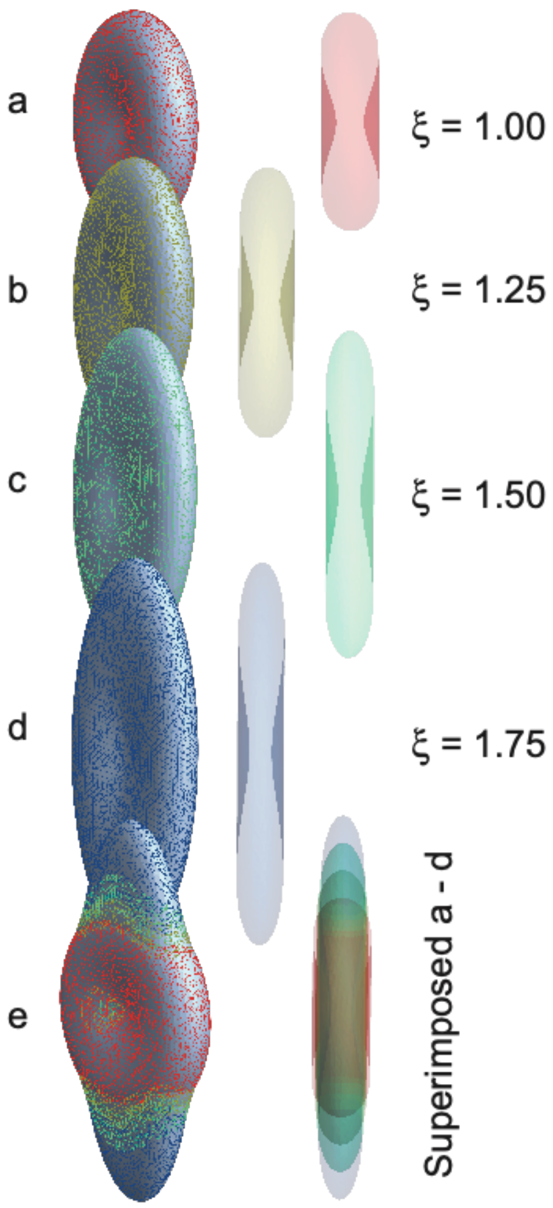

Figure S5. RBCs oriented along the *z*-axis in the *y*,*z*-plane, with a linear strain field applied in the *z*-direction. The undistorted RBC had the same dimensions as in Figure 1. RBCs (left) and their cross-sections (right): (**a**) undistorted RBC, ξ = 1; (**b**) elongated 25%, ξ = 1.25; (**c**) elongated 50%, ξ = 1.5; (**d**) elongated 75%, ξ = 1.25; and (**e**) superimposed images to convey the relative dimensions. Colour coding was used to distinguish between the RBCs in **e**.

Curvatures for θ = 0º and 90º. Figures S6 and S7 show the results of using the same *Mathematica* script as for Figure 6 but with the values of θ and ξ as indicated.

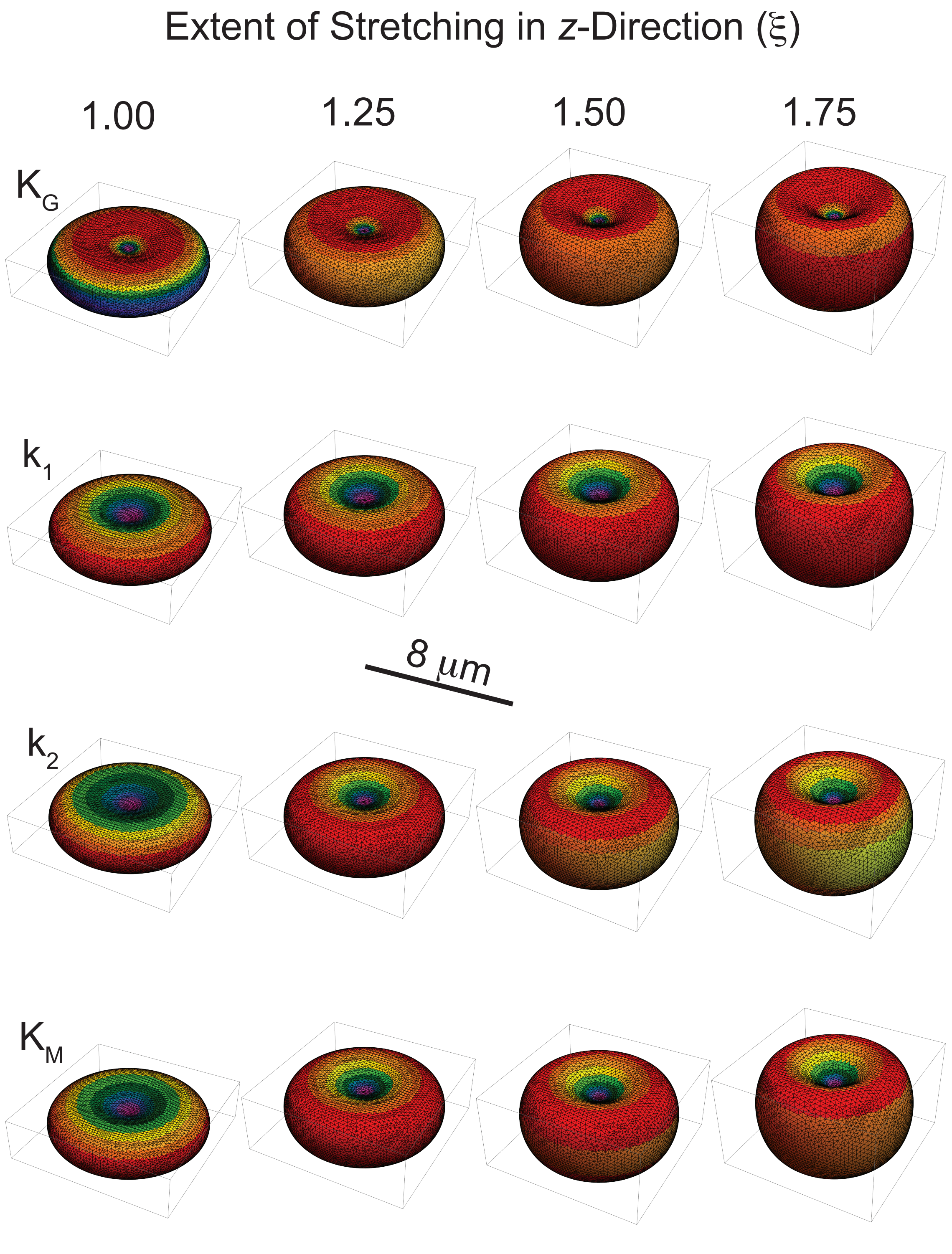

Figure S6. RBCs oriented with θ = 0º and stretched in the *z*-direction. Curvatures versus extent of stretching. The relaxed RBC had the same dimensions as in Figure 1. The central scale bar indicates $d = 8$ μm, the main diameter of the fully relaxed RBC. K_G denotes Gaussian Curvature; k_1, Principal Curvature (maximum); k_2, Principal Curvature (minimum); and K_M, Mean Curvature. The values of the respective curvatures across the 10 subdivisions of the domain of values are those shown in the central col umn of polygonal graphs in Figure 7.

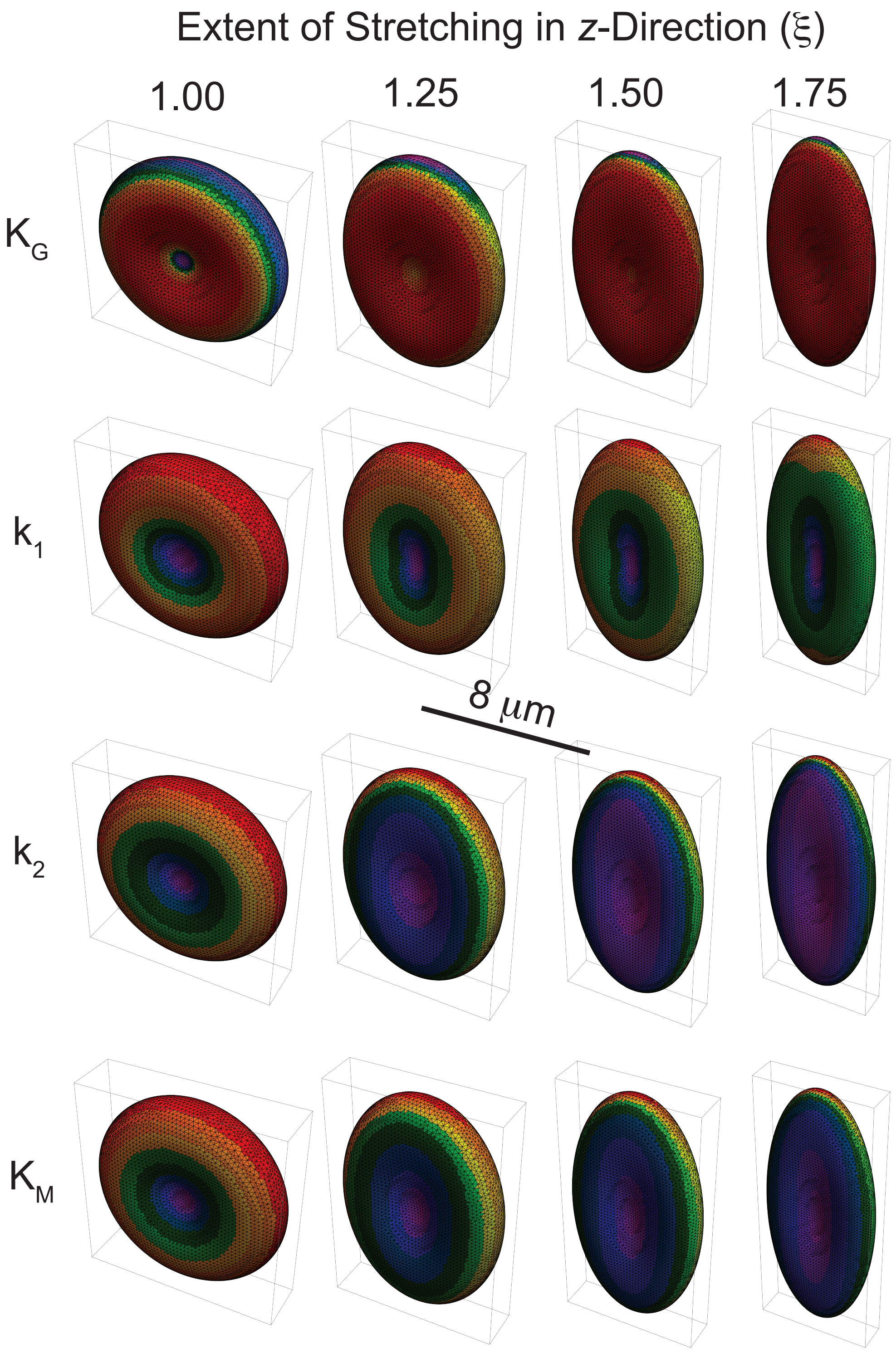

Figure S7. RBC oriented with θ = 90° and stretched in the *z*-direction. Curvatures versus extent of stretching. The central scale bar indicates $d = 8$ μm, the main diameter of the fully relaxed RBC. K_G denotes Gaussian Curvature; k_1, Principal Curvature (maximum); k_2, Principal Curvature (minimum); and K_M, Mean Curvature. The values of the respective curvatures across the 10 subdivisions of the domain of values are those shown in the central column of polygonal graphs in Figure 7.

Keeping track of each mesh-triangle

There is considerable merit in keeping track of each mesh-triangle as they undergo deformation when the RBC is stretched. For convenience, in most situations like those depicted in Figure 1-8, the surface of the RBC was distorted using the affine transform, and then it was triangularized. The differential geometry expressions of curvature were then applied to each triangle. However, when differences that arose at each triangle were needed, as for Figure 9, it was necessary to first triangularize the surface of the relaxed RBC and to then apply the affine transformation to the mesh points. This way, each triangle retained its relative position on the surface, and differences in area and curvature could be automatically computed to graph fields in which differences were either positive or negative. An example of this process is given in Notebook 7.

Supplementary Concluding Comment

The attached Notebooks provide the *Mathematica* scripts that were used to carry out the symbolic differential geometrical analysis, and to generate the figures. Additional figures that correspond to those in the main text are included here as well.

Supplementary References

1. Kuchel, P.W., et al., *Enhanced Ca^{2+} influx in mechanically distorted erythrocytes measured with ^{19}F nuclear magnetic resonance spectroscopy.* Scientific Reports, 2021. **11**(1).
2. Kuchel, P.W. and D. Shishmarev, *Accelerating metabolism and transmembrane cation flux by distorting red blood cells.* Science Advances, 2017. **3** (10): p. eaao1016.
3. Abbena, A., S. Salamon, and A. Gray, *Modern Differential Geometry of Curves and Surfaces with Mathematica*. 2006: Chapman and Hall/CRC. 1016.
4. Goldman, R., *Curvature formulas for implicit curves and surfaces.* Computer Aided Geometric Design, 2005. **22**: p. 632-658.
5. Mathematica, *Wolfram Research, Inc. (www.wolfram.com), Mathematica Online, Champaign, IL (2020).*
