# Supplementary material for: Surface model of the human red blood cell simulating changes in membrane curvature under strain
Source: Sci Rep. 2021 Jul 1;11:13712. doi: 10.1038/s41598-021-92699-7 (PMC8249411; doi:10.1038/s41598-021-92699-7)

Notebook 1 Calculations for Figure S1: Two curvatures

Plot a hyperbolic paraboloid showing the tangent plane at the origin, the two sheets, and the lines of the central parabolas through the origin. Also draw the positive and negative normal vectors to the tangent plane at the origin

```
paraboloid = ParametricPlot3D[{v, u , -u^2 + 0.3 v^2}, {u, -1, 1}, {v, -1.5, 1.5},
  AspectRatio → Automatic, Mesh → 20, MeshFunctions → {#2 &, #1 &},
  MeshStyle → {Opacity[0.2], {Black, Black}}, Axes → True,
  AxesStyle → {Directive[Black, 18], Directive[Black, 14], Directive[Black, 18]},
  PlotStyle → {Opacity[0.8], Directive[Purple]}, Boxed → True]
```

Out[ ]=

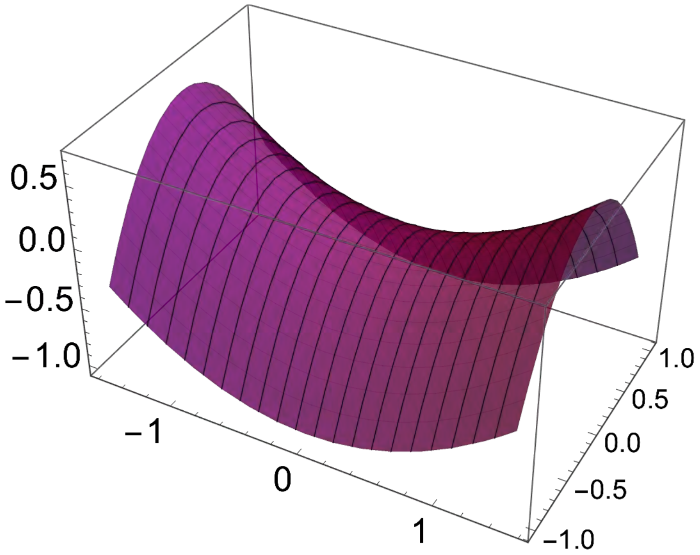

In[•]:=
```
pPlane2 = ParametricPlot3D[{v, u, 0}, {u, -1, 1},
  {v, -1.5, 1.5}, AspectRatio → Automatic, Mesh → None,
  PlotStyle → {Opacity[0.8], RGBColor[1, 0.8, 0.2]}, Axes → True, Boxed → True]
```

Out[•]=

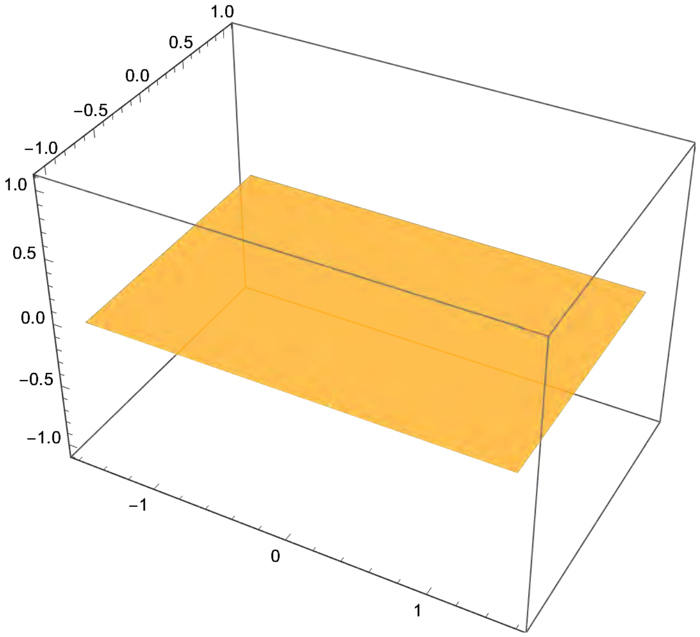

In[•]:=
```
parabola1 = Plot3D[{ 0.3 u^2 }, {u, -1.5, 1.5},
  {v, -1.5, 1.5}, AspectRatio → Automatic, Mesh → None,
  PlotStyle → {Opacity[0.8], Directive[Green]}, Boxed → False, Axes → False]
```

Out[•]=

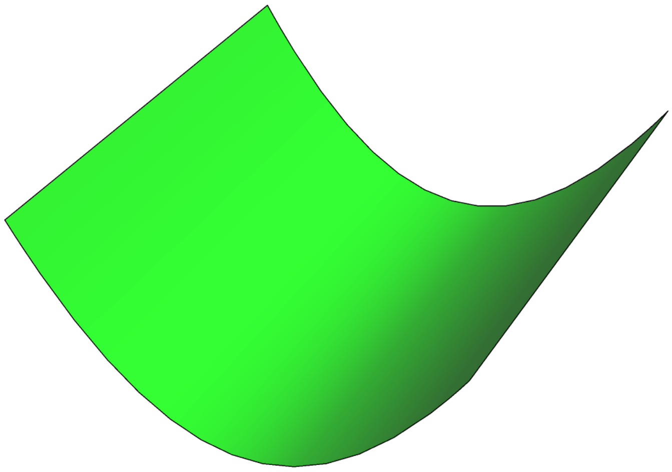

In[ ]:=

```
parabola2 =
 Plot3D[{-v^2}, {u, -1, 1}, {v, -1.5, 1.5}, AspectRatio → Automatic, Mesh → None,
  PlotStyle → {Opacity[0.5], Directive[Red]}, Boxed → False, Axes → False]
```

Out[ ]=

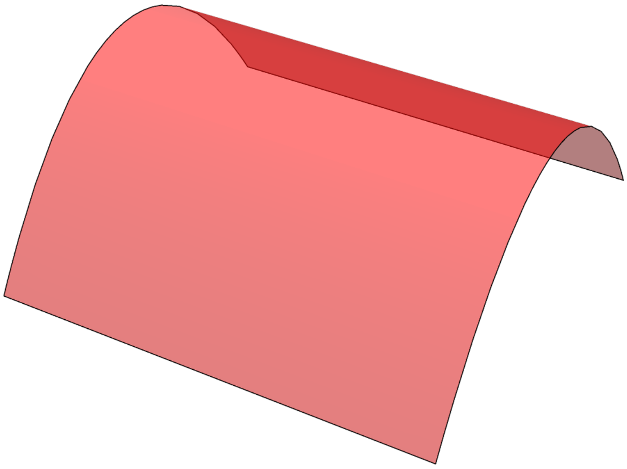

In[ ]:=

```
lineList1 = Table[{u, 0, 0.3 u^2}, {u, -1.5, 1.5, 0.1}];
lineList2 = Table[{0, v, -v^2}, {v, -1.5, 1.5, 0.1}];
line1 = Graphics3D[{Thick, Black, Line[{{0, 0, 0}, {0, 0, 1}}]}]
```

Out[ ]=

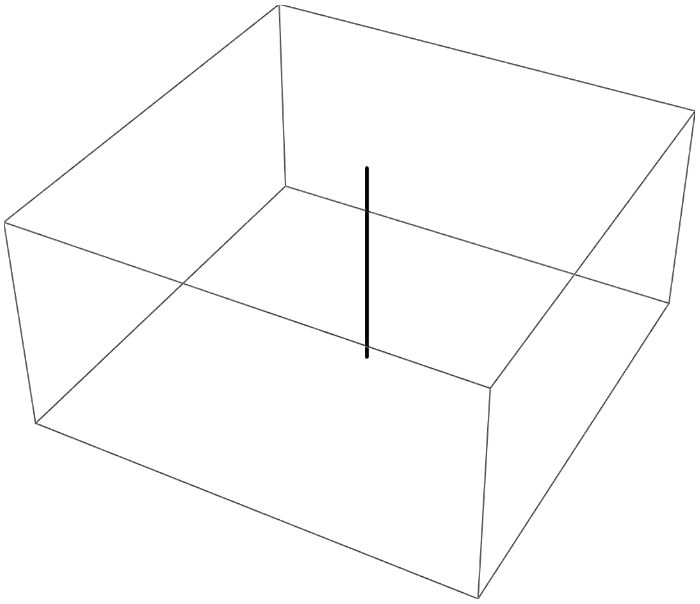

In[ ]:=

```
line2 = Graphics3D[{Thick, Black, Line[{{0, 0, 0}, {0, 0, -1}}]}]
```

Out[ ]=

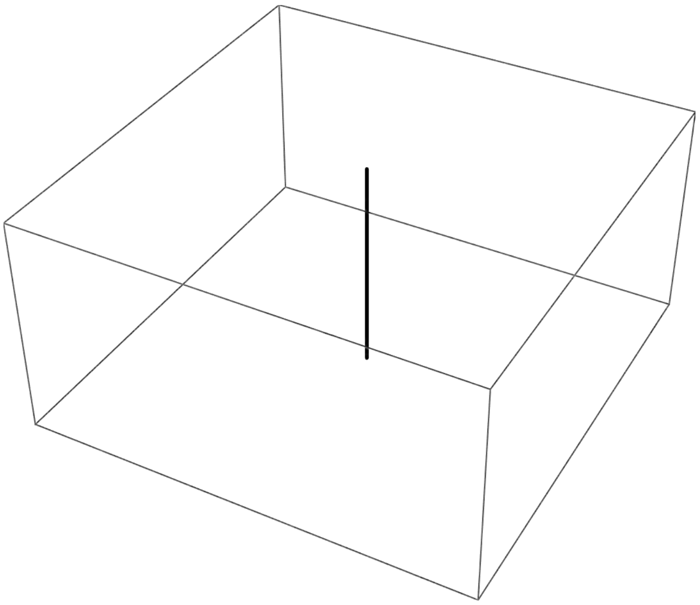

In[ ]:=

```
parab1 = Graphics3D[{Thick, Black, Line[lineList1]}]
```

Out[ ]=

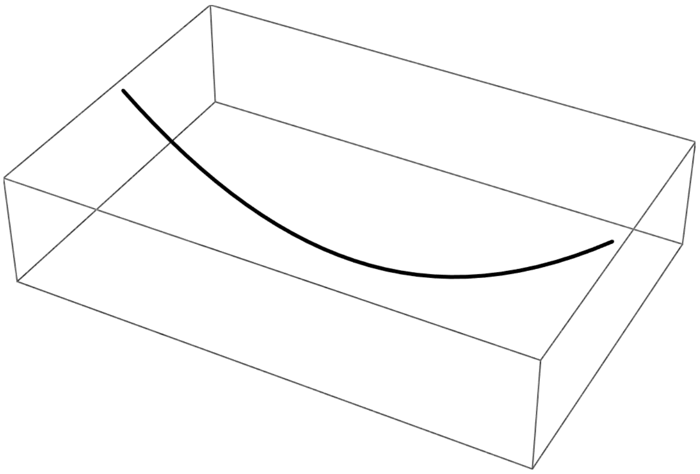

In[•]:= `parab2 = Graphics3D[{Thick, Black, Line[lineList2]}]`

Out[•]=
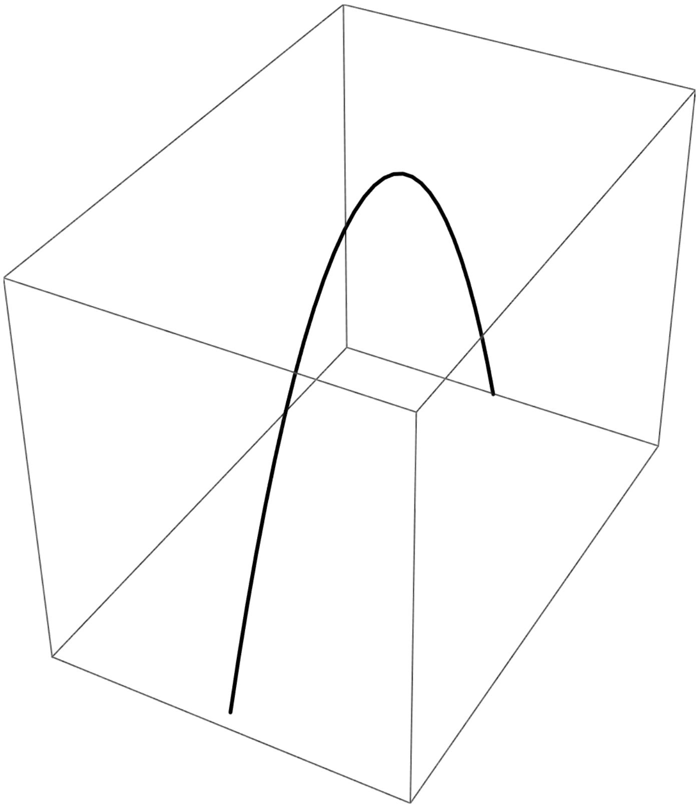

In[•]:= `ss1 = Show[{paraboloid}] (* Plot the frame and axes *)`

Out[•]=
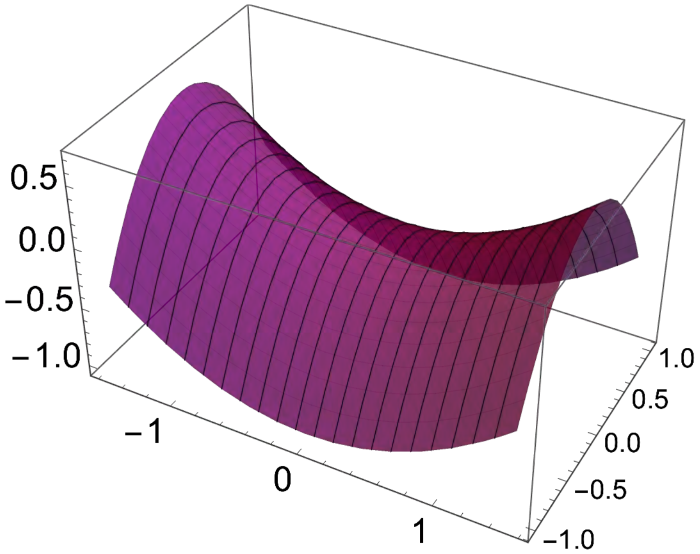

In[ ]:=
```
ss2 = Show[{pPlane2, paraboloid}, Axes → False] (* Plotthe frame and not axes *)
```

Out[ ]=

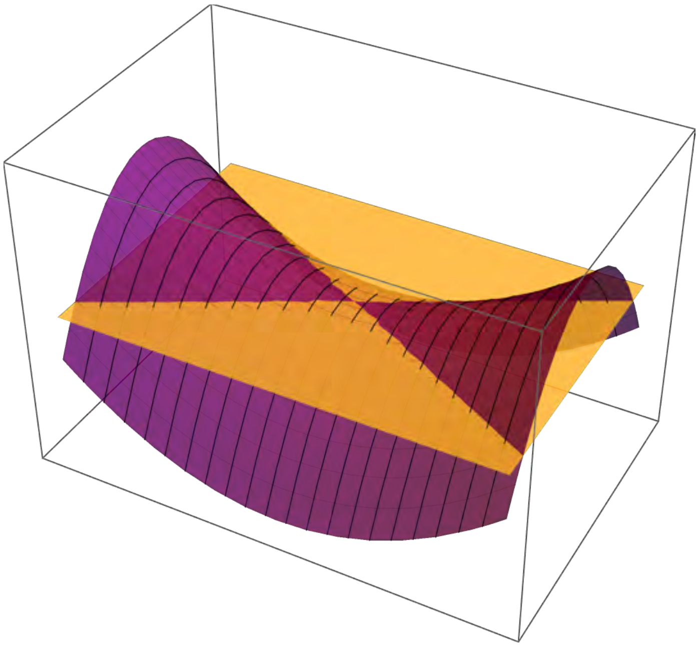

In[ ]:=
```
ss3 = Show[{pPlane2, parabola1, paraboloid, line1, line2, parab1}, Axes → False,
   Boxed → False]  (* Remove the frame and the axes to reduce clutter *)
ss4 = Show[{pPlane2, parabola1, parabola2, paraboloid, line1,
    line2, parab1, parab2}, Axes → False, Boxed → False]
   (* Remove the frame and the axes to reduce clutter *)
ss5 =
  Show[{paraboloid, pPlane2, parabola1, parabola2, line1, line2, parab1, parab2},
   Axes → True, Boxed → True] (* As for Figure S1 *)
```

Out[ ]=

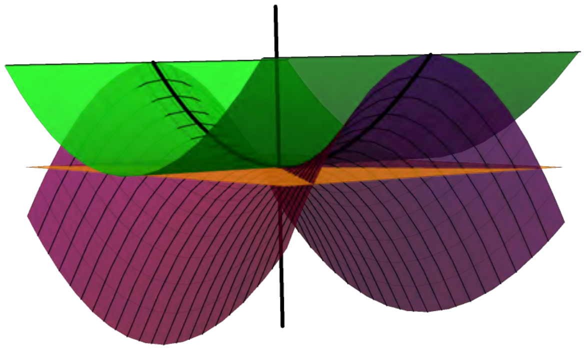

Out[ ]=

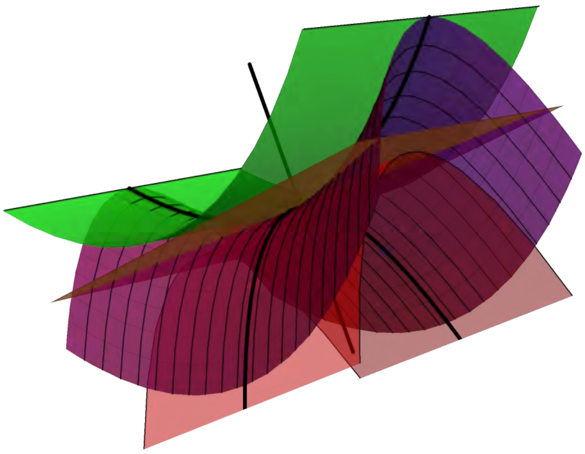

Out[ ]=

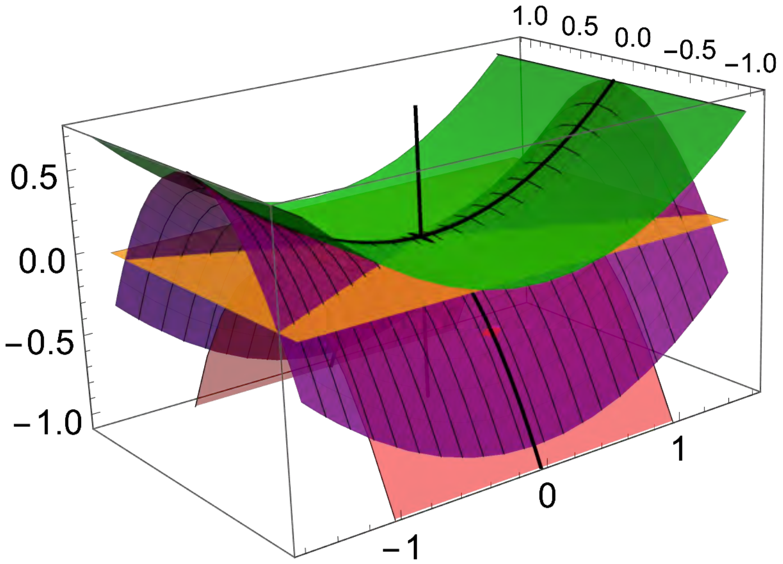

Supplement: Supplementary file 2 — Supplementary Information 2. [file 41598_2021_92699_MOESM2_ESM.pdf]
