# Supplementary material for: Surface model of the human red blood cell simulating changes in membrane curvature under strain
Source: Sci Rep. 2021 Jul 1;11:13712. doi: 10.1038/s41598-021-92699-7 (PMC8249411; doi:10.1038/s41598-021-92699-7)
Supplement: Supplementary file 3 — Supplementary Information 3. [file 41598_2021_92699_MOESM3_ESM.pdf]

Notebook 2 Calculations for Figure 1: Triangularization of the RBC surface

Compute the distribution of edge lengths in the triangularization of the RBC. Specify 242,000 α-spectrin and β-spectrin molecules per RBC and that 2 αs and 2 βs make up one side of a triangle in the cytoskeletal network/mesh. First give the three length parameter of the RBC, then the affine transform to invoke rotation and linear strain in one direction only, and then the expression for the surface of the RBC

In[ ]:=

```
Clear[d, b, h, pP, qQ, rR, ξ, θ]; (* Clear all potentially assigned values *)

d = 8.0;    (* Main diameter of the biconcave disc *)
b = 1;      (* Thickness of the biconcave disc at the centre *)
h = 2.12;  (* Maximum thickness of the biconcave disc out near the rim...
 like the width of a car tyre *)
```

$$pP = -\frac{d^2}{2} + \frac{h^2}{2}\left(\frac{d^2}{b^2} - 1\right) - \frac{h^2}{2}\left(\frac{d^2}{b^2} - 1\right)\left(1 - \frac{b^2}{h^2}\right)^{\frac{1}{2}};$$

(* Coefficient of the x^2+ y^2 term *)

$$qQ = \frac{d^2}{b^2} pP + \frac{b^2}{4}\left(\frac{d^4}{b^4} - 1\right); \quad \text{(* Coefficient of the } z^2 \text{ term *)}$$

$$rR = -\frac{d^2}{4} pP - \frac{d^4}{16}; \quad \text{(* The constant term *)}$$

```
tensorRot = {{1, 0, 0}, {0, Cos[θ], -Sin[θ]}, {0, Sin[θ], Cos[θ]}};
(* Euler rotation matrix...around the x-axis *)
tensorStretch = {{1/ √ξ , 0, 0}, {0, 1/ √ξ , 0}, {0, 0, ξ}};
(* Strain tensor...elongation in the z-direction *)
θ = 0.0 ; (* Rotation angle *)
ξ = 1.0;  (* Elongation factor...1 means original length,
2 means twice the original length etc *)

trf = InverseFunction[AffineTransform[tensorStretch.tensorRot]];
(* The affine transformation that invokes rotation and stretching together *)

rbc0 = ImplicitRegion[(x^2 + y^2 + z^2)^2 + pP (x^2 + y^2) + qQ z^2 + rR < 0 /.
    Thread[{x, y, z} → trf[{x, y, z}]], {{x, -7, 7}, {y, -7, 7}, {z, -7, 7}}];
(* RBC surface as an implict function, with the three coefficients pP,
qQ, and rR...double letters used to avoid single-
 letter symbols that could be reserved by Mathematica *)

bmr0 = BoundaryDiscretizeRegion[rbc0, MaxCellMeasure → 0.00858, AspectRatio → 1]
 (* Discretize the surface. MaxCellMeasure chosen by
  iteration of this Cell to give a mesh with ~121k edges *)
```

Out[ ]=

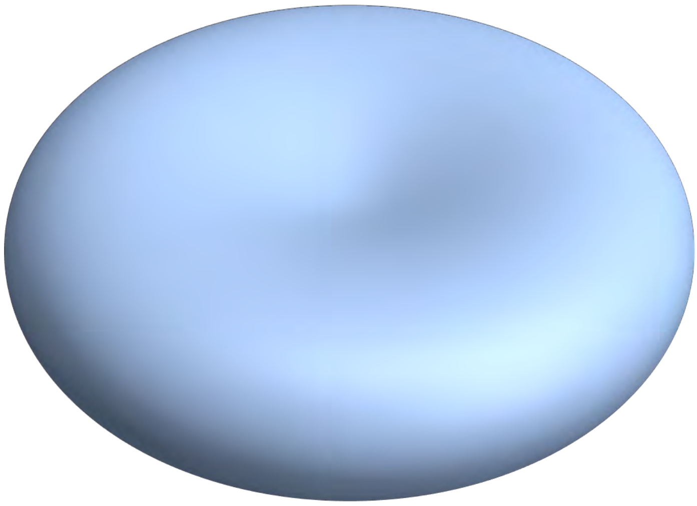

Compute the volume and surface area of the boundary discretized region. The lengths of the respective Lists give the number of mesh points, and triangles and then the number of edges is calculated from the number of triangles

In[ ]:=
```
{RegionMeasure[bmr0], RegionMeasure[RegionBoundary[bmr0]]}
RBCArea = RegionMeasure[RegionBoundary[bmr0]];
meshCoords = MeshCoordinates[bmr0];
(* The mesh coordinates come from the boundary discretized graphics values *)
meshTriangles = MeshPrimitives[bmr0, 2];
(* The list of mesh triangles is derived from the
 BoundaryDiscretizeRegion[rbc0] of the ListContourPlot3D function *)
Length@meshCoords
Length@meshTriangles
edgesNumber = 1.5 * Length@meshTriangles
```

Out[ ]= {85.969, 128.016}

Out[ ]= 40 136

Out[ ]= 80 268

Out[ ]= 120 402.

Choose a colour that best highlights the black mesh against its coloured background

In[ ]:=
```
Graphics3D[{RGBColor[0, 1, 0], Opacity[0.9],
  PolyhedronData["JabulaniPolyhedron", "Polygons"]}, Boxed → False]
```

Out[ ]=

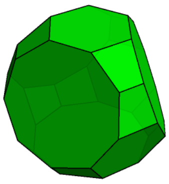

In[•]:=
```
gphGreenPlus =
  Graphics3D[{RGBColor[0, 1, 0], Opacity[0.9], meshTriangles}, Boxed → False]
```

Out[•]=

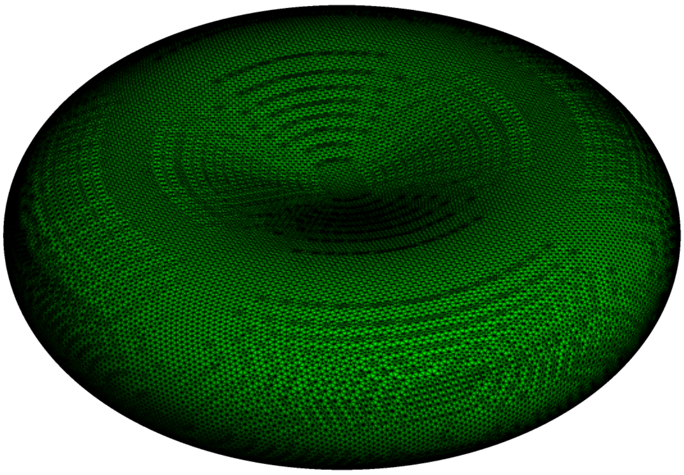

In[•]:=
```
l3 = Length[meshTriangles]
  (* Assign the value of the number of triangles to l3 *)
```

Out[•]= 80 268

Obtain the list of edges for the Length[meshTriangles] triangles ...test first with j = 1

In[•]:=
```
j = 1;

v1 = meshTriangles⟦j⟧⟦1⟧⟦1⟧;
(* meshTriangles⟦j⟧⟦1⟧ is the first element of the triangle list,
which is the three triple corrdinates...so
 meshTriangles⟦j⟧⟦1⟧⟦1⟧ is the first vertex (3-tuple) *)
v2 = meshTriangles⟦j⟧⟦1⟧⟦2⟧;
(* meshTriangles⟦j⟧⟦1⟧⟦2⟧ is the second vertex (3-tuple) *)
v3 = meshTriangles⟦j⟧⟦1⟧⟦3⟧;
(* meshTriangles⟦j⟧⟦1⟧⟦3⟧ is the third vertex (3-tuple) *)
L1 = Norm[v2 - v1]
L2 = Norm[v3 - v2]
L3 = Norm[v1 - v3]
```

Out[•]= 0.0750778

Out[•]= 0.0561406

Out[•]= 0.0650829

Perform a For loop using the Norm function on the side vectors L1, L2, and

L3, and collect all the side-lengths

In[ ]:=

```
Clear[j];

lengthList = {};
Timing[For[j = 1, j ≤ l3, j++,
   v1 = meshTriangles⟦j⟧⟦1⟧⟦1⟧;
   (* meshTriangles⟦j⟧⟦1⟧ is the first element of the triangle list,
   which is the three triple corrdinates...so meshTriangles⟦j⟧⟦1⟧⟦1⟧
    is the first vertex (3-tuple) *)
   v2 = meshTriangles⟦j⟧⟦1⟧⟦2⟧; (* meshTriangles⟦j⟧⟦1⟧⟦2⟧
    is the second vertex (3-tuple) *)
   v3 = meshTriangles⟦j⟧⟦1⟧⟦3⟧; (* meshTriangles⟦j⟧⟦1⟧⟦3⟧
    is the third vertex (3-tuple) *)
   L1 = Norm[v2 - v1];
   L2 = Norm[v3 - v2];
   L3 = Norm[v1 - v3];

   lengthList = AppendTo[lengthList, {L1, L2, L3}];
  ];]
```

Out[ ]= {29.6465, Null}

In[∘]:=
```
lengthList = Flatten@lengthList;
(* Make the side lengths into a single list *)
distribLengths = Histogram[lengthList, 20,
  ChartStyle → RGBColor[0, 1, 0], PlotRange → {{0.015, 0.11}, {0, 42 000}}]
 (* Histogram displays the frequency of occurrence of edge lengths across
  20 bins. Note that since each side is shared by two triangles the number of
  sides in the list is twice the actual number of sides in the polyhedron *)
```

Out[∘]=

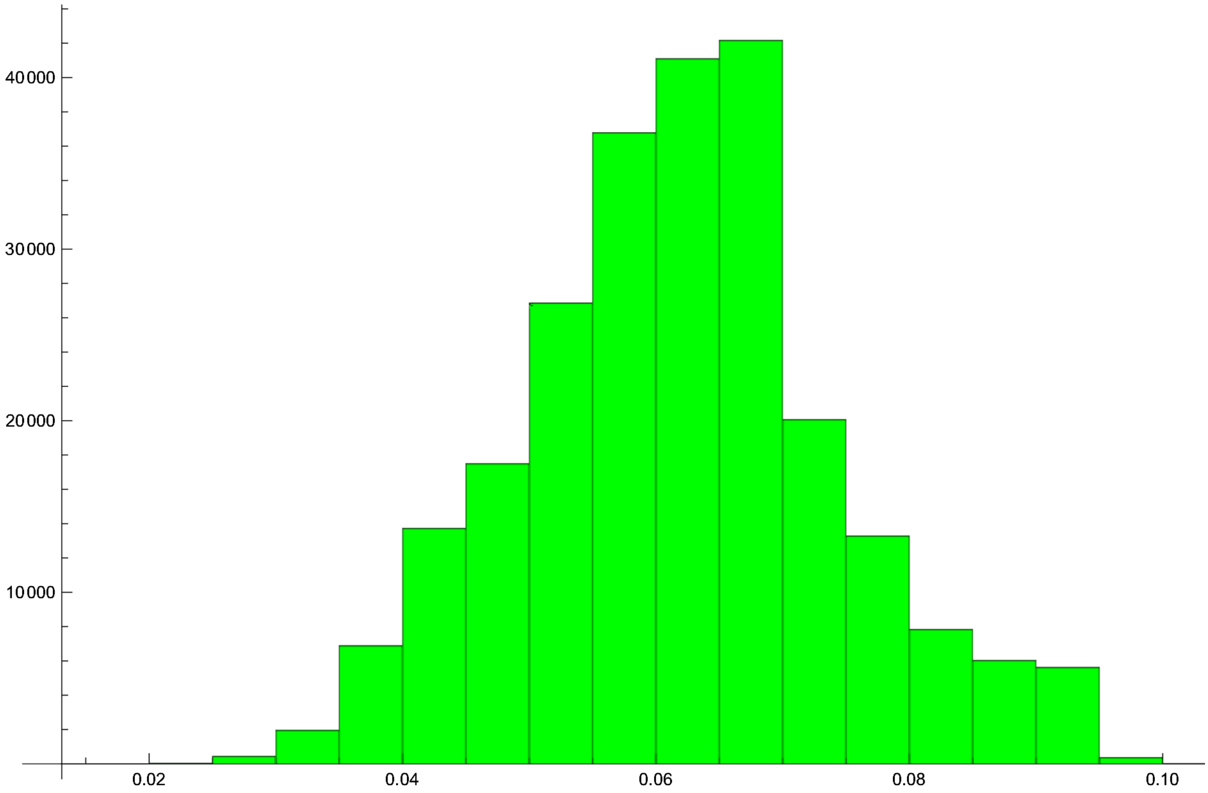

In[ ]:=
```
distribLengths2 = Histogram[lengthList, 200, ChartStyle → Red,
  PlotRange → {{0.015, 0.11}, {0, 6500}}](* This histogram that shows
  the frequency of occurrence of edge lengths across 200 bins. *)
```

Out[ ]=

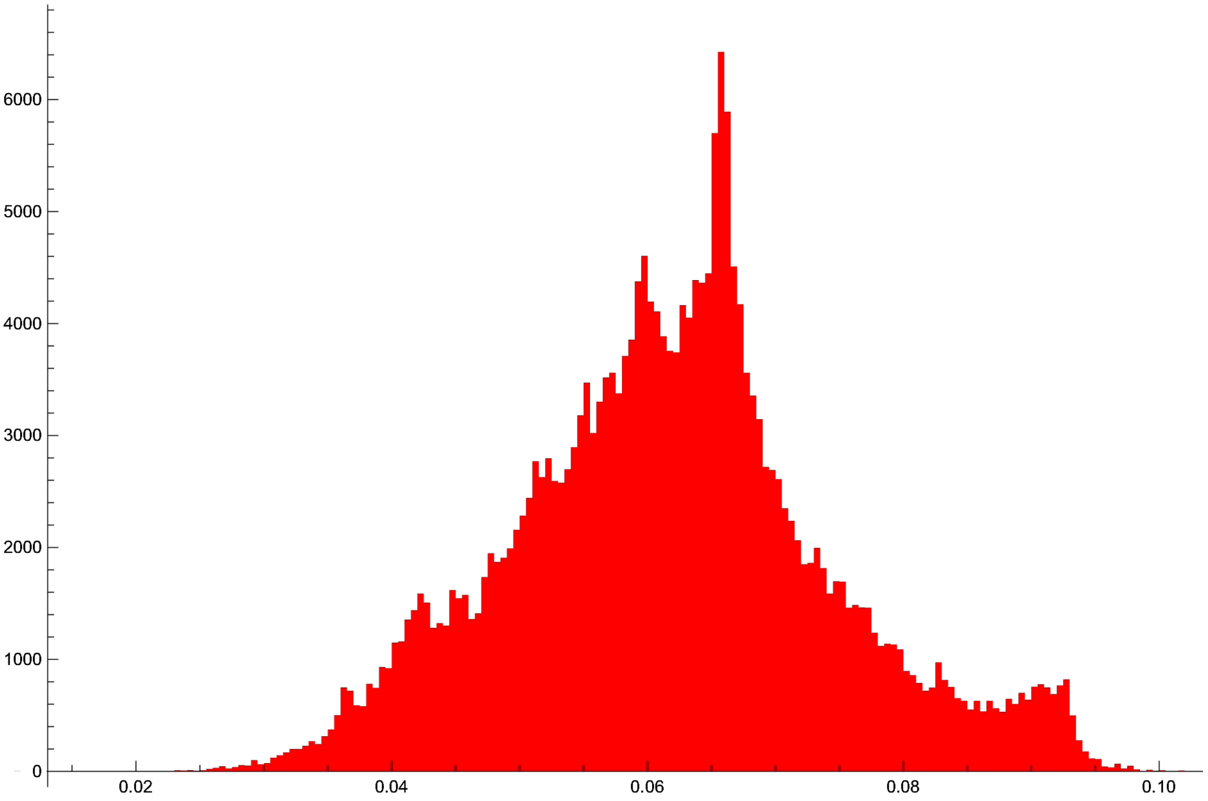

Mean length calculation...this indicates a mean edge (strut) length of 62 nm...while the median (using the Get Coordinates functionality obtained with the control key) is 44682 in the range 65 - 70 nM; or 38% of the edges...or 42744 (2nd largest group) + 44682 (1argest group) in the range 60 - 70 nm making this 75% of the edges. Note the
numbers are twice the actual numbers in the RBC because each side of a triangle is counted twice.

In[ ]:=
```
totalLength = Total@lengthList
numberOfLengths = Length@lengthList
averageLength = totalLength / numberOfLengths
```

Out[ ]= 14 902.5

Out[ ]= 240 804

Out[ ]= 0.0618863
