# Supplementary material for: Surface model of the human red blood cell simulating changes in membrane curvature under strain
Source: Sci Rep. 2021 Jul 1;11:13712. doi: 10.1038/s41598-021-92699-7 (PMC8249411; doi:10.1038/s41598-021-92699-7)

Notebook 3 Calculations for Figure 2: Length distributions of mesh

Plot the distributions of triangle areas of an RBC with 121k edges into 10 bins

In[∘]:=
```
Clear[d, b, h, pP, qQ, rR, ξ, θ];

d = 8.0;  (* Main diameter of the biconcave disc *)
b = 1;  (* Thickness of the biconcave disc at the centre *)
h = 2.12;  (* Maximum thickness of the biconcave disc out near the rim...
 like the width of a car tyre *)
```

$$pP = -\frac{d^2}{2} + \frac{h^2}{2}\left(\frac{d^2}{b^2} - 1\right) - \frac{h^2}{2}\left(\frac{d^2}{b^2} - 1\right)\left(1 - \frac{b^2}{h^2}\right)^{\frac{1}{2}} ;$$

(* Coefficient of the x^2+ y^2 term *)

$$qQ = \frac{d^2}{b^2} pP + \frac{b^2}{4}\left(\frac{d^4}{b^4} - 1\right); \quad \text{(* Coefficient of the } z^2 \text{ term *)}$$

$$rR = -\frac{d^2}{4} pP - \frac{d^4}{16} ; \quad \text{(* The constant term *)}$$

```
tensorRot = {{1, 0, 0}, {0, Cos[θ], -Sin[θ]}, {0, Sin[θ], Cos[θ]}};
tensorStretch = {{1/√ξ, 0, 0}, {0, 1/√ξ, 0}, {0, 0, ξ}};
θ = 0.0 ;
ξ = 1;

trf = InverseFunction[AffineTransform[tensorStretch.tensorRot]];
```

$$rbc0 = \text{ImplicitRegion}\left[\left(x^2 + y^2 + z^2\right)^2 + pP\left(x^2 + y^2\right) + qQ\, z^2 + rR < 0 \;/.\right.$$
$$\left.\text{Thread}[\{x, y, z\} \to \text{trf}[\{x, y, z\}]], \{\{x, -7, 7\}, \{y, -7, 7\}, \{z, -7, 7\}\}\right];$$

```
bmr0 = BoundaryDiscretizeRegion[rbc0,
   MaxCellMeasure → 0.0083, AspectRatio → 1, MeshCellStyle → LightRed];
(* Note the mesh size set to 0.2 to give a computationally
 reasonable number of triangles! *)
```

In[ ]:=
```
{RegionMeasure[bmr0], RegionMeasure[RegionBoundary[bmr0]]}
(* RBC volume and area *)
RBCArea = RegionMeasure[RegionBoundary[bmr0]];  (* RBC area *)
meshCoords = MeshCoordinates[bmr0];
(* The mesh coordinates come from the boundary discretized graphics values *)
lengthNumber = 3 Length@meshCoords
(* Number of edges in the triangularized RBC *)
```

Out[ ]= {85.9695, 128.016}

Out[ ]= 123 888

In[ ]:=
```
meshTriangles = MeshPrimitives[bmr0, 2];
(* The list of mesh triangles is derived from the
 BoundaryDiscretizeRegion[rbc0] of the ImplicitRegion function *)
```

In[ ]:=
```
l3 = Length[meshTriangles] (* Number of triangles *)
l4 = Length[meshCoords]    (* Number of mesh points *)
gphD1 = Graphics3D[{Green, meshTriangles}]
 (* The plot of the mesh triangles simply uses Graphics3D *)
```

Out[ ]= 82 588

Out[ ]= 41 296

Out[ ]=
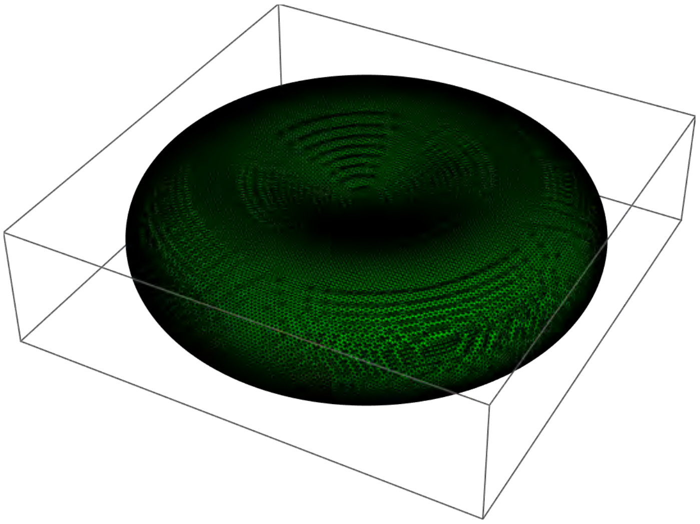

Colour code from smallest to largest values of curvature

In[ ]:=
```
colourList = {{1, 0, 0}, {1, 0.5, 0}, {1, 0.7, 0.2}, {1, 1, 0}, {0, 1, 0},
   {0, 0.5, 0}, {0, 0.5, 0.7}, {0.2, 0.4, 1}, {0.6, 0.3, 1}, {0.8, 0.2, 1}};
plotFunc := Graphics3D[{FaceForm[RGBColor[#1]], PolyhedronData[
     "JabulaniPolyhedron", "Polygons"]}, Boxed → False, ImageSize → 60] &
colourKeyPlot = plotFunc /@ colourList // Row
```

Out[ ]=

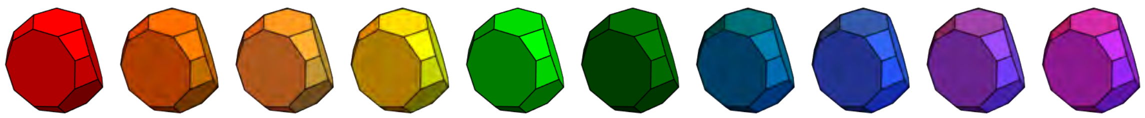

Obtain the list of areas of the l3 triangles

In[ ]:=
```
Clear[j];

triangleProps = {};  (* Name means "triangle properties" *)
For[j = 1, j ≤ l3, j++,
  v1 = meshTriangles⟦j⟧⟦1⟧⟦1⟧;
  (* meshTriangles⟦j⟧⟦1⟧ is the first element of the triangle list,
  which is the three triple corrdinates  so meshTriangles⟦j⟧⟦1⟧⟦1⟧
   is the first vertex (triple-corrdinate) *)
  v2 = meshTriangles⟦j⟧⟦1⟧⟦2⟧ ;
  (* The second vertex (triple-corrdinate) *)
  v3 = meshTriangles⟦j⟧⟦1⟧⟦3⟧; (* The third vertex (triple-corrdinate) *)
  area = (1 / 2) Cross[(v2 - v1), (v3 - v1)] // Norm;
  (* Formula for the area of a triangle: half the normal of
     the cross product of the vectors of two of the sides *)
  triangle = {j, area};
  triangleProps = AppendTo[triangleProps, triangle];
 ];
```

Find the maximum and minimum values of the areas, so each domain-span can be divided into 10 sub-domains

In[ ]:=
```
areaMin = Min@Table[triangleProps⟦i⟧ [[2]], {i, 1, l3}]
areaMax = Max@Table[triangleProps⟦i⟧ [[2]], {i, 1, l3}]
```

Out[ ]= 0.000217149

Out[ ]= 0.00334751

Plot a histogram of the triangle areas

In[∘]:=
```
areas = Flatten[Table[triangleProps⟦i, 2⟧, {i, 1, l3}]];
histArea = Histogram[areas, 20, ChartStyle → RGBColor[1, 0, 0]]
```

Out[∘]=

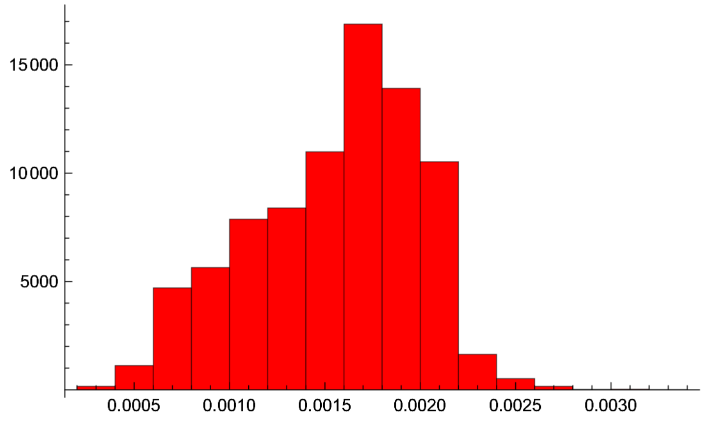

Calculate the mean area of a triangle

```
RBCArea 10^6 / l3 (* This area is in μm^2
   or multiply by 10^6 to get the number expressed in nm^2 *)
```

Out[∘]= 1550.06

Area swept out by the arms of the Piezo1 triskelion of diameter 22 nm...in nm^2

In[∘]:=
```
π 11^2 // N
(RBCArea 10^6 / l3) / (π 10^2)
```

Out[∘]= 380.133

Out[∘]= 4.93399

Divide the list of triangle areas into 10 bins with domains of ascending order of values

```
(* Create a set of 10 empty bins to
 receive the triangle index and the area *)
bin[1] = {};
bin[2] = {};
bin[3] = {};
bin[4] = {};
bin[5] = {};
bin[6] = {};
bin[7] = {};
bin[8] = {};
bin[9] = {};
bin[10] = {};

minP = areaMin;
maxP = areaMax;
delta = (maxP - minP) / 10.0;

(* This sorts the triangles according to their area *)
For[j = 1, j ≤ 10, j++,
  For[i = 1, i ≤ l3, i++,
    d = triangleProps[[i]][[2]];
    If[d ≥ (minP + (j - 1) delta) && d < (minP + j delta), AppendTo[bin[j], {i, d}]];
   ];
 ];
```

Plot each of the sets of triangles in the lists in the respective bins...show only two here to save space

```
In[ ]:= Clear[gphA];
outGraphs = For[i = 1, i ≤ 10, i++,
    indexNos = Table[Flatten[Transpose[bin[i]][[1]], 1][[j]], {j, 1, Length@bin[i]}];
    binSubSet = meshTriangles[[#]] & /@ indexNos;
    meanBin[i] = Total[Flatten[Transpose[bin[i]][[2]], 1]] / Length[bin[i]];
    gphA[i] = Graphics3D[{RGBColor[colourList[[i]]], binSubSet}];
  ];
```

In[ ]:= gphA[1]
gphA[6]

Out[ ]=

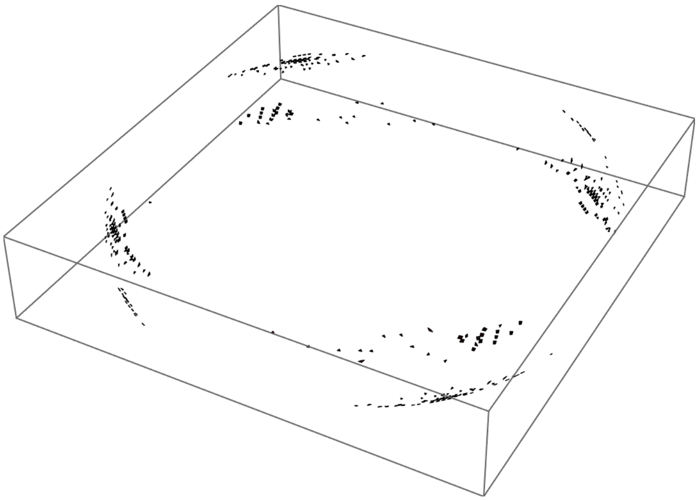

Out[ ]=

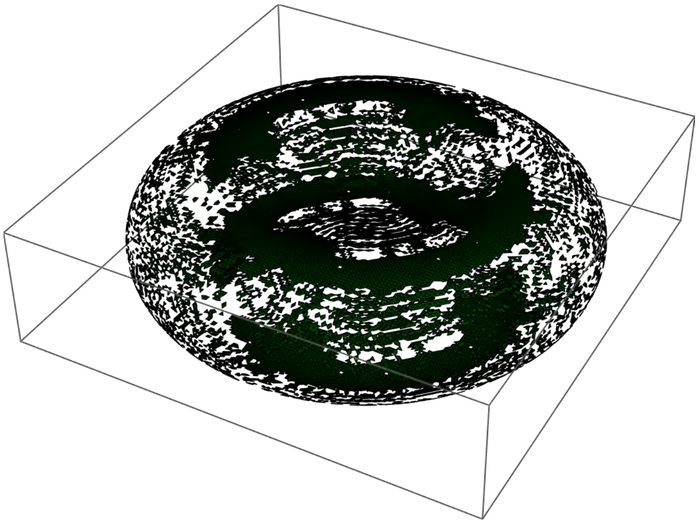

Show the full superposition of all 10 bins of triangles

In[•]:=
```
Show[{gphA[1], gphA[2], gphA[3], gphA[4],
  gphA[5], gphA[6], gphA[7], gphA[8], gphA[9], gphA[10]}]
```

Out[•]=

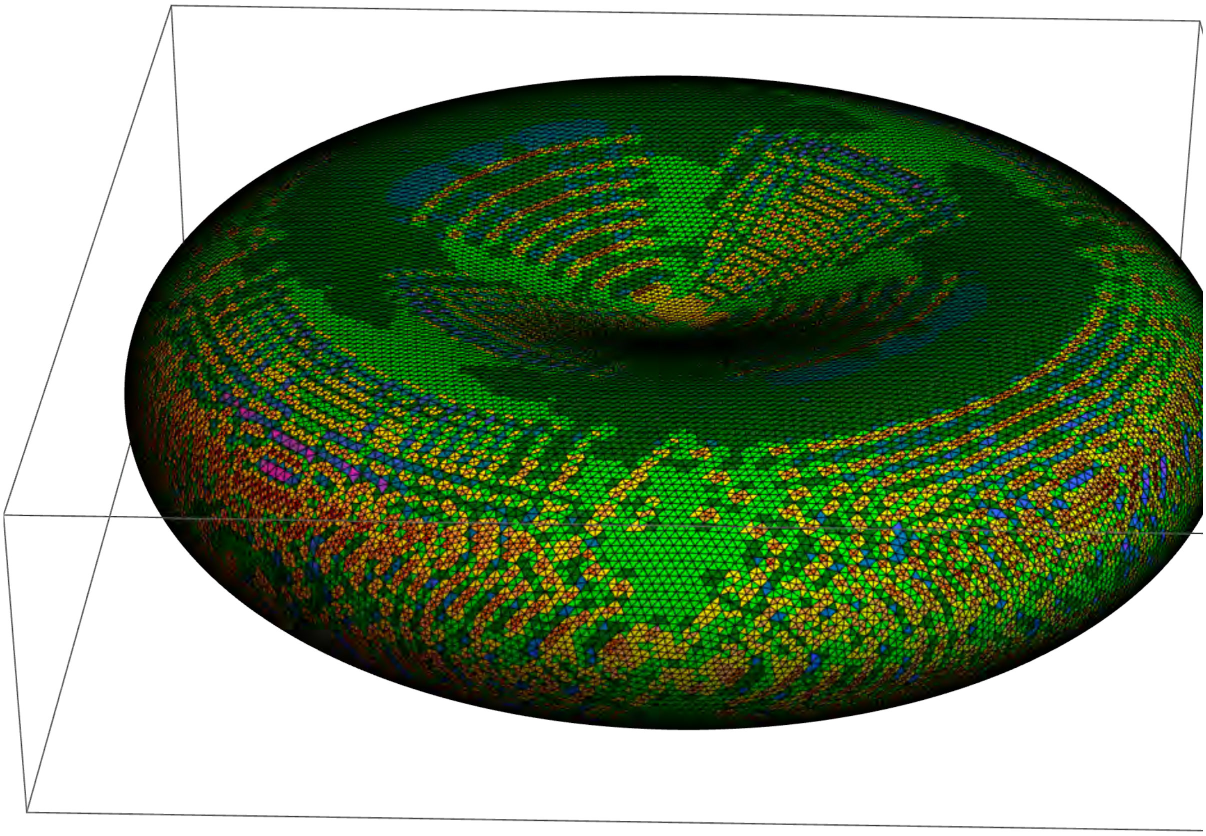

Supplement: Supplementary file 4 — Supplementary Information 4. [file 41598_2021_92699_MOESM4_ESM.pdf]
