# Supplementary material for: Surface model of the human red blood cell simulating changes in membrane curvature under strain
Source: Sci Rep. 2021 Jul 1;11:13712. doi: 10.1038/s41598-021-92699-7 (PMC8249411; doi:10.1038/s41598-021-92699-7)
Supplement: Supplementary file 5 — Supplementary Information 5. [file 41598_2021_92699_MOESM5_ESM.pdf]

Notebook 4 Symbolic calculation of the four curvature expressions for the affine transformed RBC surface

Derive the general expressions of the four curvatures of the affine-transformed RBC

```
In[ ]:= Clear[d, b, h, pP, qQ, rR, ξ, θ];

d = 8.0;   (* Main diameter of the biconcave disc *)
b = 1;     (* Thickness of the biconcave disc at the centre *)
h = 2.12;  (* Maximum thickness of the biconcave disc out near the rim...
 like the width of a car tyre *)
pP = -d^2/2 + h^2/2 (d^2/b^2 - 1) - h^2/2 (d^2/b^2 - 1) (1 - b^2/h^2)^(1/2) ;
(* Coefficient of the x^2+ y^2 term *)
qQ = d^2/b^2 pP + b^2/4 (d^4/b^4 - 1);   (* Coefficient of the z^2 term *)
rR = -d^2/4 pP - d^4/16 ; (* The constant term *)

tensorRot = {{1, 0, 0}, {0, Cos[θ], -Sin[θ]}, {0, Sin[θ], Cos[θ]}};
tensorStretch = {{1/Sqrt[ξ], 0, 0}, {0, 1/Sqrt[ξ], 0}, {0, 0, ξ}};
θ = π / 4 ;
ξ = 1;

trf = InverseFunction[AffineTransform[tensorStretch.tensorRot]];

rbc0 = ImplicitRegion[(x^2 + y^2 + z^2)^2 + pP (x^2 + y^2) + qQ z^2 + rR < 0 /.
    Thread[{x, y, z} → trf[{x, y, z}]], {{x, -7, 7}, {y, -7, 7}, {z, -7, 7}}];

bmr0 = BoundaryDiscretizeRegion[rbc0, MaxCellMeasure → 0.2, AspectRatio → 1]
 (* Note the mesh size set to 0.2 to give a
  computationally reasonable number of triangles! *)
```

Out[•]=

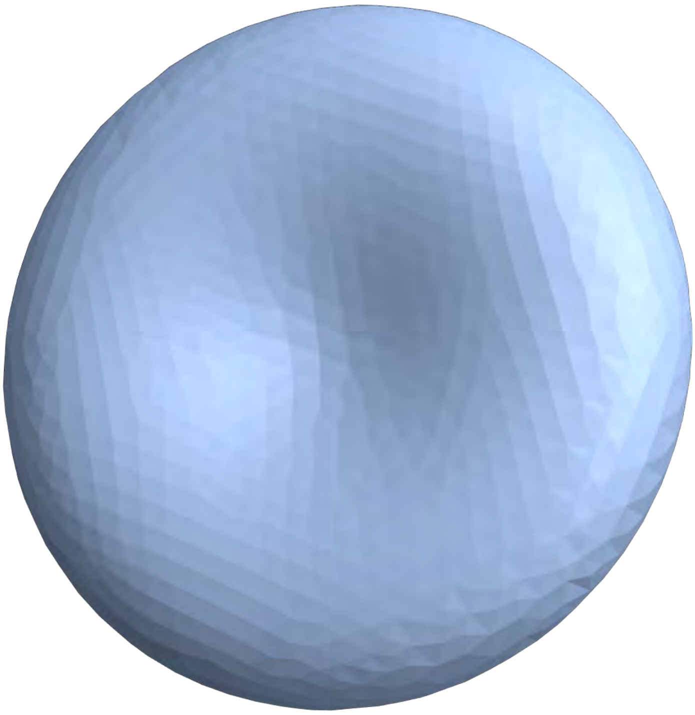

Test the AffineTransform and InverseFunction aspects of the analysis

In[•]:=
```
Clear[pP, qQ, rR, θ, ξ];
tensorRot = {{1, 0, 0}, {0, Cos[θ], -Sin[θ]}, {0, Sin[θ], Cos[θ]}};
tensorStretch = {{1/ √ξ, 0, 0}, {0, 1/ √ξ, 0}, {0, 0, ξ}};
trf = InverseFunction[AffineTransform[tensorStretch.tensorRot]];
trf[{xx, yy, zz}]
```

Out[•]=

$$\left\{xx\sqrt{\xi},\ \frac{yy\sqrt{\xi}\,\mathrm{Cos}[\theta]}{\mathrm{Cos}[\theta]^2+\mathrm{Sin}[\theta]^2}+\frac{zz\,\mathrm{Sin}[\theta]}{\xi\left(\mathrm{Cos}[\theta]^2+\mathrm{Sin}[\theta]^2\right)},\ \frac{zz\,\mathrm{Cos}[\theta]}{\xi\left(\mathrm{Cos}[\theta]^2+\mathrm{Sin}[\theta]^2\right)}-\frac{yy\sqrt{\xi}\,\mathrm{Sin}[\theta]}{\mathrm{Cos}[\theta]^2+\mathrm{Sin}[\theta]^2}\right\}$$

In[ ]:= $(x^2+y^2+z^2)^2 + pP\,(x^2+y^2) + qQ\,z^2 + rR$ /.

$\left\{\left\{x \to x\sqrt{\xi},\ y \to \frac{y\sqrt{\xi}\ \mathrm{Cos}[\theta]}{\mathrm{Cos}[\theta]^2+\mathrm{Sin}[\theta]^2} + \frac{z\,\mathrm{Sin}[\theta]}{\xi\,(\mathrm{Cos}[\theta]^2+\mathrm{Sin}[\theta]^2)},\ z \to \frac{z\,\mathrm{Cos}[\theta]}{\xi\,(\mathrm{Cos}[\theta]^2+\mathrm{Sin}[\theta]^2)} - \frac{y\sqrt{\xi}\ \mathrm{Sin}[\theta]}{\mathrm{Cos}[\theta]^2+\mathrm{Sin}[\theta]^2}\right\}\right\}$

Out[ ]= $\left\{rR + qQ\left(\frac{z\,\mathrm{Cos}[\theta]}{\xi\,(\mathrm{Cos}[\theta]^2+\mathrm{Sin}[\theta]^2)} - \frac{y\sqrt{\xi}\ \mathrm{Sin}[\theta]}{\mathrm{Cos}[\theta]^2+\mathrm{Sin}[\theta]^2}\right)^2 + pP\left(x^2\,\xi + \left(\frac{y\sqrt{\xi}\ \mathrm{Cos}[\theta]}{\mathrm{Cos}[\theta]^2+\mathrm{Sin}[\theta]^2} + \frac{z\,\mathrm{Sin}[\theta]}{\xi\,(\mathrm{Cos}[\theta]^2+\mathrm{Sin}[\theta]^2)}\right)^2\right) + \left(x^2\,\xi + \left(\frac{y\sqrt{\xi}\ \mathrm{Cos}[\theta]}{\mathrm{Cos}[\theta]^2+\mathrm{Sin}[\theta]^2} + \frac{z\,\mathrm{Sin}[\theta]}{\xi\,(\mathrm{Cos}[\theta]^2+\mathrm{Sin}[\theta]^2)}\right)^2 + \left(\frac{z\,\mathrm{Cos}[\theta]}{\xi\,(\mathrm{Cos}[\theta]^2+\mathrm{Sin}[\theta]^2)} - \frac{y\sqrt{\xi}\ \mathrm{Sin}[\theta]}{\mathrm{Cos}[\theta]^2+\mathrm{Sin}[\theta]^2}\right)^2\right)^2\right\}$

Use the definitions of the function *F*[x,y,z] and derivative matrices as specified by Ron Goldman and in Alfred Gray’s book

In[ ]:= fF := $rR + qQ\left(\frac{z\,\mathrm{Cos}[\theta]}{\xi\,(\mathrm{Cos}[\theta]^2+\mathrm{Sin}[\theta]^2)} - \frac{y\sqrt{\xi}\ \mathrm{Sin}[\theta]}{\mathrm{Cos}[\theta]^2+\mathrm{Sin}[\theta]^2}\right)^2 + pP\left(x^2\,\xi + \left(\frac{y\sqrt{\xi}\ \mathrm{Cos}[\theta]}{\mathrm{Cos}[\theta]^2+\mathrm{Sin}[\theta]^2} + \frac{z\,\mathrm{Sin}[\theta]}{\xi\,(\mathrm{Cos}[\theta]^2+\mathrm{Sin}[\theta]^2)}\right)^2\right) + \left(x^2\,\xi + \left(\frac{y\sqrt{\xi}\ \mathrm{Cos}[\theta]}{\mathrm{Cos}[\theta]^2+\mathrm{Sin}[\theta]^2} + \frac{z\,\mathrm{Sin}[\theta]}{\xi\,(\mathrm{Cos}[\theta]^2+\mathrm{Sin}[\theta]^2)}\right)^2 + \left(\frac{z\,\mathrm{Cos}[\theta]}{\xi\,(\mathrm{Cos}[\theta]^2+\mathrm{Sin}[\theta]^2)} - \frac{y\sqrt{\xi}\ \mathrm{Sin}[\theta]}{\mathrm{Cos}[\theta]^2+\mathrm{Sin}[\theta]^2}\right)^2\right)^2$

```
(* The discocyte surface defined by F[x,y,z]*)

delF = {D[fF, x], D[fF, y], D[fF, z]} ;
(* Ron Goldman's curvature theory uses these derivative functions *)
HF = {{D[D[fF, x], x], D[D[fF, x], y], D[D[fF, x], z]},
   {D[D[fF, y], x], D[D[fF, y], y], D[D[fF, y], z]},
   {D[D[fF, z], x], D[D[fF, z], y], D[D[fF, z], z]}};
```

```
In[•]:= Cofactor[m_List?MatrixQ, {i_Integer, j_Integer}] :=
         (-1) ^ (i + j) Det[Drop[Transpose[Drop[Transpose[m], {j}]], {i}]]
        (* A neat way of calculating the expression for the Cofactor
         matrix was obtained from Mathematica's MathWorld *)

        HStarF = {{Cofactor[HF, {1, 1}], Cofactor[HF, {1, 2}], Cofactor[HF, {1, 3}]},
             {Cofactor[HF, {2, 1}], Cofactor[HF, {2, 2}], Cofactor[HF, {2, 3}]}, {Cofactor[
               HF, {3, 1}], Cofactor[HF, {3, 2}], Cofactor[HF, {3, 3}]}} // Simplify;
        trHF = Tr[HF] // Simplify ;(*Trace of the Hessian matrix *)
```

The expressions for the Gaussian and Mean Curvatures follow...(Note Abs denotes the absolute value)

```
In[•]:= κG = delF.HStarF.Transpose[delF] / Norm[delF]^4 // Simplify ;
        (* Gaussian curvature *)
        κM = (delF.HF.Transpose[delF] - Norm[delF]^2 trHF) / (2 Norm[delF]^3) // Simplify ;
        (* Mean Curvature *)
```

The expressions for the Principal Curvatures k $_1$ and k $_2$ follow very simply from the two above...

```
In[•]:= PCk1 = κM + Sqrt[κM^2 - κG] ;
        PCk2 = κM - Sqrt[κM^2 - κG] ;
```

where the square roots are printed as $\sqrt{\kappa M^2 - \kappa G}$.
