# Supplementary material for: Surface model of the human red blood cell simulating changes in membrane curvature under strain
Source: Sci Rep. 2021 Jul 1;11:13712. doi: 10.1038/s41598-021-92699-7 (PMC8249411; doi:10.1038/s41598-021-92699-7)
Supplement: Supplementary file 6 — Supplementary Information 6. [file 41598_2021_92699_MOESM6_ESM.pdf]

Notebook 5 Test for Total Curvature of the RBC: Effectively a finite difference approximation of the surface integral of the Gaussian curvature based on the mesh

First, obviate the routine derivation of the Gaussian and Mean Curvature expressions that are in the previous NoteBooks

```
In[•]:= Clear[x, y, z, ξ, θ, pP, qQ, rR];
ϰGFunc[x_, y_, z_] :=
  (8 × (2 z^2 + ξ^2 (pP + 2 (x^2 + y^2) ξ)) × (16 z^8 + 12 pP z^6 ξ^2 + 12 qQ z^6 ξ^2 + 64 x^2 z^6 ξ^3 +
      64 y^2 z^6 ξ^3 + 3 pP^2 z^4 ξ^4 + 6 pP qQ z^4 ξ^4 + 3 qQ^2 z^4 ξ^4 + 40 pP x^2 z^4 ξ^5 + 32 qQ x^2 z^4 ξ^5 +
      36 pP y^2 z^4 ξ^5 + 36 qQ y^2 z^4 ξ^5 + pP^2 qQ z^2 ξ^6 + pP qQ^2 z^2 ξ^6 + 96 x^4 z^4 ξ^6 +
      192 x^2 y^2 z^4 ξ^6 + 96 y^4 z^4 ξ^6 + 10 pP^2 x^2 z^2 ξ^7 + 8 pP qQ x^2 z^2 ξ^7 + 6 qQ^2 x^2 z^2 ξ^7 +
      6 pP^2 y^2 z^2 ξ^7 + 12 pP qQ y^2 z^2 ξ^7 + 6 qQ^2 y^2 z^2 ξ^7 + 44 pP x^4 z^2 ξ^8 + 28 qQ x^4 z^2 ξ^8 +
      80 pP x^2 y^2 z^2 ξ^8 + 64 qQ x^2 y^2 z^2 ξ^8 + 36 pP y^4 z^2 ξ^8 + 36 qQ y^4 z^2 ξ^8 + 2 pP^2 qQ x^2 ξ^9 +
      pP^2 qQ y^2 ξ^9 + pP qQ^2 y^2 ξ^9 + 64 x^6 z^2 ξ^9 + 192 x^4 y^2 z^2 ξ^9 + 192 x^2 y^4 z^2 ξ^9 +
      64 y^6 z^2 ξ^9 + 4 pP^2 x^4 ξ^10 + 8 pP qQ x^4 ξ^10 + 10 pP^2 x^2 y^2 ξ^10 + 8 pP qQ x^2 y^2 ξ^10 +
      6 qQ^2 x^2 y^2 ξ^10 + 3 pP^2 y^4 ξ^10 + 6 pP qQ y^4 ξ^10 + 3 qQ^2 y^4 ξ^10 + 16 pP x^6 ξ^11 +
      8 qQ x^6 ξ^11 + 44 pP x^4 y^2 ξ^11 + 28 qQ x^4 y^2 ξ^11 + 40 pP x^2 y^4 ξ^11 + 32 qQ x^2 y^4 ξ^11 +
      12 pP y^6 ξ^11 + 12 qQ y^6 ξ^11 + 16 x^8 ξ^12 + 64 x^6 y^2 ξ^12 + 96 x^4 y^4 ξ^12 + 64 x^2 y^6 ξ^12 +
      16 y^8 ξ^12 + (pP - qQ) ξ^2 (-z^2 + y^2 ξ^3) (4 z^4 + 2 z^2 ξ^2 (pP + qQ + 4 (x^2 + y^2) ξ) +
         ξ^4 (pP (qQ + 2 (-x^2 + y^2) ξ) + 2 ξ (qQ (3 x^2 + y^2) + 2 (x^2 + y^2)^2 ξ))) Cos[2 θ] -
      (pP - qQ)^2 ξ^4 (z^4 - 6 y^2 z^2 ξ^3 + y^4 ξ^6) Cos[4 θ] + 8 pP y z^5 ξ^(7/2) Sin[2 θ] -
      8 qQ y z^5 ξ^(7/2) Sin[2 θ] + 4 pP^2 y z^3 ξ^(11/2) Sin[2 θ] - 4 qQ^2 y z^3 ξ^(11/2) Sin[2 θ] +
      16 pP x^2 y z^3 ξ^(13/2) Sin[2 θ] - 16 qQ x^2 y z^3 ξ^(13/2) Sin[2 θ] + 16 pP y^3 z^3 ξ^(13/2) Sin[2 θ] -
      16 qQ y^3 z^3 ξ^(13/2) Sin[2 θ] + 2 pP^2 qQ y z ξ^(15/2) Sin[2 θ] - 2 pP qQ^2 y z ξ^(15/2) Sin[2 θ] -
      4 pP^2 x^2 y z ξ^(17/2) Sin[2 θ] + 16 pP qQ x^2 y z ξ^(17/2) Sin[2 θ] -
      12 qQ^2 x^2 y z ξ^(17/2) Sin[2 θ] + 4 pP^2 y^3 z ξ^(17/2) Sin[2 θ] - 4 qQ^2 y^3 z ξ^(17/2) Sin[2 θ] +
      8 pP x^4 y z ξ^(19/2) Sin[2 θ] - 8 qQ x^4 y z ξ^(19/2) Sin[2 θ] + 16 pP x^2 y^3 z ξ^(19/2) Sin[2 θ] -
      16 qQ x^2 y^3 z ξ^(19/2) Sin[2 θ] + 8 pP y^5 z ξ^(19/2) Sin[2 θ] - 8 qQ y^5 z ξ^(19/2) Sin[2 θ] +
      4 pP^2 y z^3 ξ^(11/2) Sin[4 θ] - 8 pP qQ y z^3 ξ^(11/2) Sin[4 θ] + 4 qQ^2 y z^3 ξ^(11/2) Sin[4 θ] -
      4 pP^2 y^3 z ξ^(17/2) Sin[4 θ] + 8 pP qQ y^3 z ξ^(17/2) Sin[4 θ] - 4 qQ^2 y^3 z ξ^(17/2) Sin[4 θ])) /
   (ξ^10 (Abs[(4 x z^2)/ξ + 2 x ξ (pP + 2 (x^2 + y^2) ξ)]^2 + Abs[(4 y z^2)/ξ + y ξ (pP + qQ + 4 (x^2 + y^2) ξ) +
          (pP - qQ) y ξ Cos[2 θ] + ((pP - qQ) z Sin[2 θ])/Sqrt[ξ]]^2 + Abs[1/ξ^4 (4 z^3 + z ξ^2 (pP + qQ +
```

```
      4 (x^2 + y^2) ξ) - (pP - qQ) z ξ^2 Cos[2 θ] + (pP - qQ) y ξ^(7/2) Sin[2 θ])]^2)^2)

κMFunc[x_, y_, z_] := (-ξ^8 (Abs[(4 x z^2)/ξ + 2 x ξ (pP + 2 (x^2 + y^2) ξ)]^2 +
      Abs[(4 y z^2)/ξ + y ξ (pP + qQ + 4 (x^2 + y^2) ξ) + (pP - qQ) y ξ Cos[2 θ] +
         ((pP - qQ) z Sin[2 θ])/Sqrt[ξ]]^2 + Abs[1/ξ^4 (4 z^3 + z ξ^2 (pP + qQ + 4 (x^2 + y^2) ξ) -
         (pP - qQ) z ξ^2 Cos[2 θ] + (pP - qQ) y ξ^(7/2) Sin[2 θ])]^2)
    (4 z^2 (3 + 2 ξ^3) + ξ^2 (pP + 3 pP ξ^3 + qQ (1 + ξ^3) + 4 (x^2 + y^2) ξ (1 + 4 ξ^3)) +
      (pP - qQ) ξ^2 (-1 + ξ^3) Cos[2 θ]) +
    8 x^2 ξ^6 (2 z^2 + ξ^2 (pP + 2 (x^2 + y^2) ξ)) × (8 z^4 + 2 pP z^2 ξ^2 + 2 qQ z^2 ξ^2 + 8 x^2 z^2 ξ^3 +
      8 y^2 z^2 ξ^3 + 4 z^4 ξ^3 + 4 pP z^2 ξ^5 + 16 x^2 z^2 ξ^6 + 16 y^2 z^2 ξ^6 + pP^2 ξ^7 +
      8 pP x^2 ξ^8 + 6 pP y^2 ξ^8 + 2 qQ y^2 ξ^8 + 12 x^4 ξ^9 + 24 x^2 y^2 ξ^9 + 12 y^4 ξ^9 +
      2 (pP - qQ) ξ^2 (-z^2 + y^2 ξ^6) Cos[2 θ] + 2 (pP - qQ) y z ξ^(7/2) (1 + ξ^3) Sin[2 θ]) +
    (4 z^3 + z ξ^2 (pP + qQ + 4 (x^2 + y^2) ξ) - (pP - qQ) z ξ^2 Cos[2 θ] + (pP - qQ) y ξ^(7/2) Sin[2 θ]) ×
     (16 x^2 z ξ^6 (2 z^2 + ξ^2 (pP + 2 (x^2 + y^2) ξ)) +
      ξ^6 (8 y z + (pP - qQ) Sqrt[ξ] Sin[2 θ]) × (4 y z^2 + y ξ^2 (pP + qQ + 4 (x^2 + y^2) ξ) +
         (pP - qQ) y ξ^2 Cos[2 θ] + (pP - qQ) z Sqrt[ξ] Sin[2 θ]) +
      (12 z^2 + ξ^2 (pP + qQ + 4 (x^2 + y^2) ξ) - (pP - qQ) ξ^2 Cos[2 θ]) × (4 z^3 + z ξ^2
         (pP + qQ + 4 (x^2 + y^2) ξ) - (pP - qQ) z ξ^2 Cos[2 θ] + (pP - qQ) y ξ^(7/2) Sin[2 θ])) +
    ξ^6 (4 y z^2 + y ξ^2 (pP + qQ + 4 (x^2 + y^2) ξ) + (pP - qQ) y ξ^2 Cos[2 θ] +
      (pP - qQ) z Sqrt[ξ] Sin[2 θ]) × (16 x^2 y ξ^6 (2 z^2 + ξ^2 (pP + 2 (x^2 + y^2) ξ)) +
      ξ^3 (4 z^2 + ξ^2 (pP + qQ + 4 (x^2 + 3 y^2) ξ) + (pP - qQ) ξ^2 Cos[2 θ]) × (4 y z^2 + y ξ^2
         (pP + qQ + 4 (x^2 + y^2) ξ) + (pP - qQ) y ξ^2 Cos[2 θ] + (pP - qQ) z Sqrt[ξ] Sin[2 θ]) +
      (8 y z + (pP - qQ) Sqrt[ξ] Sin[2 θ]) × (4 z^3 + z ξ^2 (pP + qQ + 4 (x^2 + y^2) ξ) -
         (pP - qQ) z ξ^2 Cos[2 θ] + (pP - qQ) y ξ^(7/2) Sin[2 θ]))) /
  (2 ξ^12 (Abs[(4 x z^2)/ξ + 2 x ξ (pP + 2 (x^2 + y^2) ξ)]^2 + Abs[(4 y z^2)/ξ + y ξ (pP + qQ + 4 (x^2 + y^2) ξ) +
         (pP - qQ) y ξ Cos[2 θ] + ((pP - qQ) z Sin[2 θ])/Sqrt[ξ]]^2 + Abs[1/ξ^4 (4 z^3 + z ξ^2 (pP + qQ +
            4 (x^2 + y^2) ξ) - (pP - qQ) z ξ^2 Cos[2 θ] + (pP - qQ) y ξ^(7/2) Sin[2 θ])]^2)^(3/2))
```

In[ ]:=
```
Clear[d, b, h, pP, qQ, rR, ξ, θ];

d = 8.0;  (* Main diameter of the biconcave disc *)
b = 1;  (* Thickness of the biconcave disc at the centre *)
h = 2.12;  (* Maximum thickness of the biconcave disc out near the rim...
 like the width of a car tyre *)
```

$$pP = -\frac{d^2}{2} + \frac{h^2}{2}\left(\frac{d^2}{b^2} - 1\right) - \frac{h^2}{2}\left(\frac{d^2}{b^2} - 1\right)\left(1 - \frac{b^2}{h^2}\right)^{\frac{1}{2}};$$

(* Coefficient of the x^2+ y^2 term *)

$$qQ = \frac{d^2}{b^2} pP + \frac{b^2}{4}\left(\frac{d^4}{b^4} - 1\right); \quad \text{(* Coefficient of the } z^2 \text{ term *)}$$

$$rR = -\frac{d^2}{4} pP - \frac{d^4}{16}; \quad \text{(* The constant term *)}$$

```
tensorRot = {{1, 0, 0}, {0, Cos[θ], -Sin[θ]}, {0, Sin[θ], Cos[θ]}};
tensorStretch = {{1/ √ξ , 0, 0}, {0, 1/ √ξ , 0}, {0, 0, ξ}};
θ = 0.0 ;
ξ = 1.0;

trf = InverseFunction[AffineTransform[tensorStretch.tensorRot]];

rbc0 = ImplicitRegion[(x^2 + y^2 + z^2)^2 + pP (x^2 + y^2) + qQ z^2 + rR < 0 /.
    Thread[{x, y, z} → trf[{x, y, z}]], {{x, -7, 7}, {y, -7, 7}, {z, -7, 7}}];


bmr0 = BoundaryDiscretizeRegion[
  rbc0, MaxCellMeasure → 0.3, AspectRatio → Automatic,
  MeshCellStyle → {{2, All} → Opacity[0.5, RGBColor[0.5, 0.8, 0]],
    {1, All} → Red, {0, All} → Directive[PointSize[Medium], Black]}]
 (* Note the mesh size set to 0.2 to give a computationally
  reasonable number of triangles! *)
```

Out[ ]=

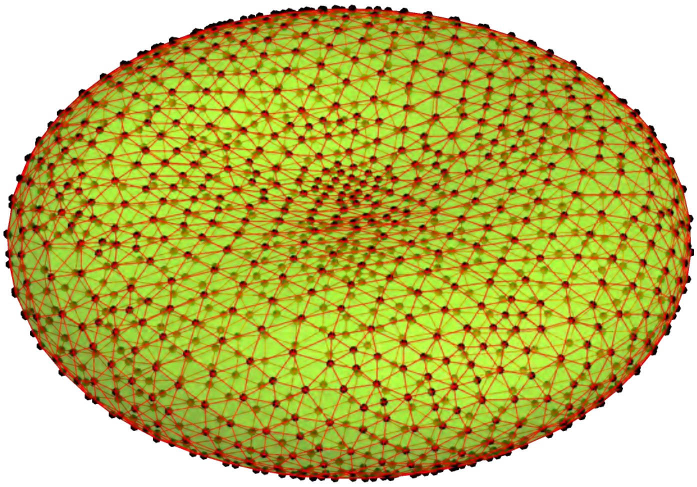

In[]:=
```
{RegionMeasure[bmr0], RegionMeasure[RegionBoundary[bmr0]]}
RBCArea = RegionMeasure[RegionBoundary[bmr0]]
meshCoords = MeshCoordinates[bmr0] ;
(* The mesh coordinates come from the boundary discretized graphics values *)
meshTriangles = MeshPrimitives[bmr0, 2] ;
(* The list of mesh triangles is derived from the
 BoundaryDiscretizeRegion[rbc0] of the ListContourPlot3D function *)
```

Out[]= {85.1991, 127.467}

Out[]= 127.467

In[]:=
```
l3 = Length[meshTriangles]
l4 = Length[meshCoords]
gphD1 = Graphics3D[{Red, meshTriangles}]
 (* The plot of the mesh triangles simply uses Graphics3D *)
```

Out[]= 2216

Out[]= 1110

Out[]=
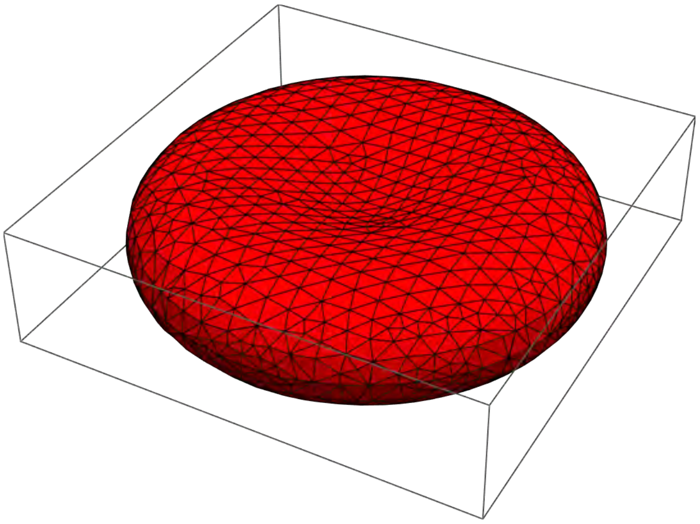

In[]:=
```
v1 = meshTriangles[[1]][[1]][[1]]
v2 = meshTriangles[[1]][[1]][[2]]
v3 = meshTriangles[[1]][[1]][[3]]
```

Out[]= {1.53805, 0.805468, 0.949967}

Out[]= {1.5188, 1.15195, 0.988641}

Out[]= {1.15817, 0.880882, 0.872647}

Colour code from smallest to largest values of curvature

In[∘]:=
```
colourList = {{1, 0, 0}, {1, 0.5, 0}, {1, 0.7, 0.2}, {1, 1, 0}, {0, 1, 0},
    {0, 0.5, 0}, {0, 0.5, 0.7}, {0.2, 0.4, 1}, {0.6, 0.3, 1}, {0.8, 0.2, 1}};
plotFunc := Graphics3D[{FaceForm[RGBColor[#1]], PolyhedronData[
      "JabulaniPolyhedron", "Polygons"]}, Boxed → False, ImageSize → 60] &
colourKeyPlot = plotFunc /@ colourList // Row
```

Out[∘]=

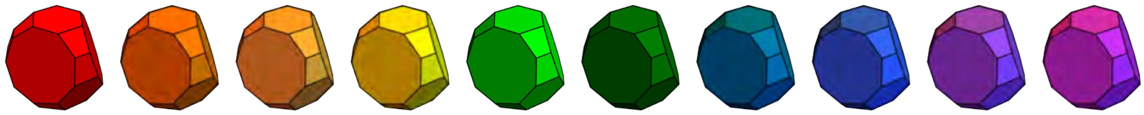

Obtain the average of the values of the curvatures at each of the three vertices...using triangle 1 as an example to test things out...incidentally get the centroids and areas too

In[∘]:=
```
j = 1;

v1 = meshTriangles⟦j⟧⟦1⟧⟦1⟧
(* meshTriangles⟦j⟧⟦1⟧ is the first element of the triangle list,
which is the three triple corrdinates  so
 meshTriangles⟦j⟧⟦1⟧⟦1⟧ is the first vertex (triple-corrdinate) *)
v2 = meshTriangles⟦j⟧⟦1⟧⟦2⟧ (* meshTriangles⟦j⟧⟦1⟧ is the first element
 of the triangle list, which is the three triple corrdinates so
 meshTriangles⟦j⟧⟦1⟧⟦2⟧ is the second vertex (triple-corrdinate) *)
v3 = meshTriangles⟦j⟧⟦1⟧⟦3⟧ (* meshTriangles⟦j⟧⟦1⟧ is the first element
 of the triangle list, which is the three triple corrdinates so
 meshTriangles⟦j⟧⟦1⟧⟦3⟧ is the third vertex (triple-corrdinate) *)
centroid = (v1 + v2 + v3) / 3; (* Formula for the centroid of
 a triangle whose three vertex coordinates are known *)
area = (1 / 2) Cross[(v2 - v1), (v3 - v1)] // Norm;
(* Formula for the area of a triangle:
  half the normal of the cross product of the vectors of two of the sides *)

c1 = κGFunc[centroid⟦1⟧, centroid⟦2⟧, centroid⟦3⟧];
(* Apply the Gaussian Curvature function with the three coordinates *)
c2 = κMFunc[centroid⟦1⟧, centroid⟦2⟧, centroid⟦3⟧];
(* Apply the Mean Curvature function with the three coordinates *)

(* Apply the Gaussian and Mean Curvature formulae to the three vertices *)
v1GC = κGFunc[meshTriangles⟦j, 1, 1, 1⟧,
   meshTriangles⟦j, 1, 1, 2⟧, meshTriangles⟦j, 1, 1, 3⟧];
v1MC = κMFunc[meshTriangles⟦j, 1, 1, 1⟧, meshTriangles⟦j, 1, 1, 2⟧,
   meshTriangles⟦j, 1, 1, 3⟧];
v1k1 = v1MC + √(v1MC^2 - v1GC) // Re;
v1k2 = v1MC - √(v1MC^2 - v1GC) // Re;
```

```
v2GC = κGFunc[meshTriangles⟦j, 1, 2, 1⟧,
   meshTriangles⟦j, 1, 2, 2⟧, meshTriangles⟦j, 1, 2, 3⟧];
v2MC = κMFunc[meshTriangles⟦j, 1, 2, 1⟧, meshTriangles⟦j, 1, 2, 2⟧,
   meshTriangles⟦j, 1, 2, 3⟧];
v2k1 = v2MC + Sqrt[v2MC^2 - v2GC] // Re;
v2k2 = v2MC - Sqrt[v2MC^2 - v2GC] // Re;

v3GC = κGFunc[meshTriangles⟦j, 1, 3, 1⟧,
   meshTriangles⟦j, 1, 3, 2⟧, meshTriangles⟦j, 1, 3, 3⟧];
v3MC = κMFunc[meshTriangles⟦j, 1, 3, 1⟧, meshTriangles⟦j, 1, 3, 2⟧,
   meshTriangles⟦j, 1, 3, 3⟧];
v3k1 = v3MC + Sqrt[v3MC^2 - v3GC] // Re;
v3k2 = v3MC - Sqrt[v3MC^2 - v3GC] // Re;

aveGC = (v1GC + v2GC + v3GC) / 3
aveMC = (v1MC + v2MC + v3MC) / 3
avek1 = (v1k1 + v2k1 + v3k1) / 3
avek2 = (v1k2 + v2k2 + v3k2) / 3
```

Out[•]= {1.53805, 0.805468, 0.949967}

Out[•]= {1.5188, 1.15195, 0.988641}

Out[•]= {1.15817, 0.880882, 0.872647}

Out[•]= -0.026561

Out[•]= -0.0221277

Out[•]= 0.147262

Out[•]= -0.191518

In[•]:=

```
Clear[j];

triangleProperties = {};
For[j = 1, j ≤ l3, j ++,
  v1 = meshTriangles⟦j⟧⟦1⟧⟦1⟧;
  (* meshTriangles⟦j⟧⟦1⟧ is the first element of the triangle list,
  which is the three triple corrdinates  so meshTriangles⟦j⟧⟦1⟧⟦1⟧
   is the first vertex (triple-corrdinate) *)
  v2 = meshTriangles⟦j⟧⟦1⟧⟦2⟧ ;
  (* meshTriangles⟦j⟧⟦1⟧ is the first element of the triangle list,
  which is the three triple corrdinates so meshTriangles⟦j⟧⟦1⟧⟦2⟧
   is the second vertex (triple-corrdinate) *)
  v3 = meshTriangles⟦j⟧⟦1⟧⟦3⟧; (* meshTriangles⟦j⟧⟦1⟧ is the first
```

```
     element of the triangle list, which is the three triple corrdinates
     so meshTriangles[[j]][[1]][[3]] is the third vertex (triple-corrdinate) *)
    centroid = (v1 + v2 + v3) / 3; (* Formula for the centroid of
     a triangle whose three vertex coordinates are known *)
    area = (1 / 2) Cross[(v2 - v1), (v3 - v1)] // Norm;
    (* Formula for the area of a triangle: half the normal of
       the cross product of the vectors of two of the sides *)

    c1 = κGFunc[centroid[[1]], centroid[[2]], centroid[[3]]];
    (* Apply the Gaussian Curvature function with the three coordinates *)
    c2 = κMFunc[centroid[[1]], centroid[[2]], centroid[[3]]];
    (* Apply the Mean Curvature function with the three coordinates *)

    (* Apply the Gaussian and Mean Curvature formulae to the three vertices *)
    v1GC = κGFunc[meshTriangles[[j, 1, 1, 1]],
      meshTriangles[[j, 1, 1, 2]], meshTriangles[[j, 1, 1, 3]]];
    v1MC = κMFunc[meshTriangles[[j, 1, 1, 1]], meshTriangles[[j, 1, 1, 2]],
      meshTriangles[[j, 1, 1, 3]]];
    v1k1 = v1MC + Sqrt[v1MC^2 - v1GC];
    v1k2 = v1MC - Sqrt[v1MC^2 - v1GC];

    v2GC = κGFunc[meshTriangles[[j, 1, 2, 1]],
      meshTriangles[[j, 1, 2, 2]], meshTriangles[[j, 1, 2, 3]]];
    v2MC = κMFunc[meshTriangles[[j, 1, 2, 1]], meshTriangles[[j, 1, 2, 2]],
      meshTriangles[[j, 1, 2, 3]]];
    v2k1 = v2MC + Sqrt[v2MC^2 - v2GC];
    v2k2 = v2MC - Sqrt[v2MC^2 - v2GC];

    v3GC = κGFunc[meshTriangles[[j, 1, 3, 1]],
      meshTriangles[[j, 1, 3, 2]], meshTriangles[[j, 1, 3, 3]]];
    v3MC = κMFunc[meshTriangles[[j, 1, 3, 1]], meshTriangles[[j, 1, 3, 2]],
      meshTriangles[[j, 1, 3, 3]]];
    v3k1 = v3MC + Sqrt[v3MC^2 - v3GC];
    v3k2 = v3MC - Sqrt[v3MC^2 - v3GC];

    aveGC = (v1GC + v2GC + v3GC) / 3;
    aveMC = (v1MC + v2MC + v3MC) / 3;
    avek1 = (v1k1 + v2k1 + v3k1) / 3;
    avek2 = (v1k2 + v2k2 + v3k2) / 3;
    triangle = {centroid, area, c1, c2, aveGC, aveMC, avek1, avek2};
    triangleProperties = AppendTo[triangleProperties, triangle];
   ];
```

In[ ]:=
```
triangleProperties[[1]] (* OK it works *)
triangleProperties[[1000]]
```

```
Out[•]= {{1.40501, 0.946099, 0.937085}, 0.0672457, -0.0282544,
  -0.0225513, -0.026561, -0.0221277, 0.147262, -0.191518}

Out[•]= {{-0.764657, -0.636456, 0.725825}, 0.0442398,
  0.00371171, 0.165201, 0.00740439, 0.167262, 0.320157, 0.0143675}
```

Now test what the weighted sum of the Gaussian Curvatures is to see if it comes to 2π...

```
In[•]:= totalAreaRBC = Total@Table[triangleProperties[[j]][[2]], {j, 1, l3}]
        (* This is correct *)
        areaWeightedGC = Total@
          Table[triangleProperties[[j]][[2]] × triangleProperties[[j]][[5]], {j, 1, l3}] / (4 π)

Out[•]= 127.467

Out[•]= 0.993282
```

This value of 0.999 is very close to the 1.0 expected for the Total Curvature...implying that it is 4π as predicted by the Gauss-Bonnet Theorem

```
In[•]:= areaIfWeightedGC = Total@
          Table[triangleProperties[[j]][[2]] * triangleProperties[[j]][[5]], {j, 1, l3}] / (4 π)

Out[•]= 0.993282
```
