# Supplementary material for: Surface model of the human red blood cell simulating changes in membrane curvature under strain
Source: Sci Rep. 2021 Jul 1;11:13712. doi: 10.1038/s41598-021-92699-7 (PMC8249411; doi:10.1038/s41598-021-92699-7)
Supplement: Supplementary file 7 — Supplementary Information 7. [file 41598_2021_92699_MOESM7_ESM.pdf]

# Notebook 6 Calculations for figures like Figures 3 and S6 and S7: Colour coded distribution of curvatures on the RBC surface

Obviate the routine derivation of the Gaussian and Mean Curvature expressions that are in the previous NoteBooks...

```
In[•]:= Clear[x, y, z, ξ, θ, pP, qQ, rR];
κGFunc[x_, y_, z_] :=
 (8 × (2 z^2 + ξ^2 (pP + 2 (x^2 + y^2) ξ)) × (16 z^8 + 12 pP z^6 ξ^2 + 12 qQ z^6 ξ^2 + 64 x^2 z^6 ξ^3 +
     64 y^2 z^6 ξ^3 + 3 pP^2 z^4 ξ^4 + 6 pP qQ z^4 ξ^4 + 3 qQ^2 z^4 ξ^4 + 40 pP x^2 z^4 ξ^5 + 32 qQ x^2 z^4 ξ^5 +
     36 pP y^2 z^4 ξ^5 + 36 qQ y^2 z^4 ξ^5 + pP^2 qQ z^2 ξ^6 + pP qQ^2 z^2 ξ^6 + 96 x^4 z^4 ξ^6 +
     192 x^2 y^2 z^4 ξ^6 + 96 y^4 z^4 ξ^6 + 10 pP^2 x^2 z^2 ξ^7 + 8 pP qQ x^2 z^2 ξ^7 + 6 qQ^2 x^2 z^2 ξ^7 +
     6 pP^2 y^2 z^2 ξ^7 + 12 pP qQ y^2 z^2 ξ^7 + 6 qQ^2 y^2 z^2 ξ^7 + 44 pP x^4 z^2 ξ^8 + 28 qQ x^4 z^2 ξ^8 +
     80 pP x^2 y^2 z^2 ξ^8 + 64 qQ x^2 y^2 z^2 ξ^8 + 36 pP y^4 z^2 ξ^8 + 36 qQ y^4 z^2 ξ^8 + 2 pP^2 qQ x^2 ξ^9 +
     pP^2 qQ y^2 ξ^9 + pP qQ^2 y^2 ξ^9 + 64 x^6 z^2 ξ^9 + 192 x^4 y^2 z^2 ξ^9 + 192 x^2 y^4 z^2 ξ^9 +
     64 y^6 z^2 ξ^9 + 4 pP^2 x^4 ξ^10 + 8 pP qQ x^4 ξ^10 + 10 pP^2 x^2 y^2 ξ^10 + 8 pP qQ x^2 y^2 ξ^10 +
     6 qQ^2 x^2 y^2 ξ^10 + 3 pP^2 y^4 ξ^10 + 6 pP qQ y^4 ξ^10 + 3 qQ^2 y^4 ξ^10 + 16 pP x^6 ξ^11 +
     8 qQ x^6 ξ^11 + 44 pP x^4 y^2 ξ^11 + 28 qQ x^4 y^2 ξ^11 + 40 pP x^2 y^4 ξ^11 + 32 qQ x^2 y^4 ξ^11 +
     12 pP y^6 ξ^11 + 12 qQ y^6 ξ^11 + 16 x^8 ξ^12 + 64 x^6 y^2 ξ^12 + 96 x^4 y^4 ξ^12 + 64 x^2 y^6 ξ^12 +
     16 y^8 ξ^12 + (pP - qQ) ξ^2 (-z^2 + y^2 ξ^3) (4 z^4 + 2 z^2 ξ^2 (pP + qQ + 4 (x^2 + y^2) ξ) +
        ξ^4 (pP (qQ + 2 (-x^2 + y^2) ξ) + 2 ξ (qQ (3 x^2 + y^2) + 2 (x^2 + y^2)^2 ξ))) Cos[2 θ] -
     (pP - qQ)^2 ξ^4 (z^4 - 6 y^2 z^2 ξ^3 + y^4 ξ^6) Cos[4 θ] + 8 pP y z^5 ξ^(7/2) Sin[2 θ] -
     8 qQ y z^5 ξ^(7/2) Sin[2 θ] + 4 pP^2 y z^3 ξ^(11/2) Sin[2 θ] - 4 qQ^2 y z^3 ξ^(11/2) Sin[2 θ] +
     16 pP x^2 y z^3 ξ^(13/2) Sin[2 θ] - 16 qQ x^2 y z^3 ξ^(13/2) Sin[2 θ] + 16 pP y^3 z^3 ξ^(13/2) Sin[2 θ] -
     16 qQ y^3 z^3 ξ^(13/2) Sin[2 θ] + 2 pP^2 qQ y z ξ^(15/2) Sin[2 θ] - 2 pP qQ^2 y z ξ^(15/2) Sin[2 θ] -
     4 pP^2 x^2 y z ξ^(17/2) Sin[2 θ] + 16 pP qQ x^2 y z ξ^(17/2) Sin[2 θ] -
     12 qQ^2 x^2 y z ξ^(17/2) Sin[2 θ] + 4 pP^2 y^3 z ξ^(17/2) Sin[2 θ] - 4 qQ^2 y^3 z ξ^(17/2) Sin[2 θ] +
     8 pP x^4 y z ξ^(19/2) Sin[2 θ] - 8 qQ x^4 y z ξ^(19/2) Sin[2 θ] + 16 pP x^2 y^3 z ξ^(19/2) Sin[2 θ] -
     16 qQ x^2 y^3 z ξ^(19/2) Sin[2 θ] + 8 pP y^5 z ξ^(19/2) Sin[2 θ] - 8 qQ y^5 z ξ^(19/2) Sin[2 θ] +
     4 pP^2 y z^3 ξ^(11/2) Sin[4 θ] - 8 pP qQ y z^3 ξ^(11/2) Sin[4 θ] + 4 qQ^2 y z^3 ξ^(11/2) Sin[4 θ] -
     4 pP^2 y^3 z ξ^(17/2) Sin[4 θ] + 8 pP qQ y^3 z ξ^(17/2) Sin[4 θ] - 4 qQ^2 y^3 z ξ^(17/2) Sin[4 θ])) /
  (ξ^10 (Abs[(4 x z^2)/ξ + 2 x ξ (pP + 2 (x^2 + y^2) ξ)]^2 + Abs[(4 y z^2)/ξ + y ξ (pP + qQ + 4 (x^2 + y^2) ξ) +
          (pP - qQ) y ξ Cos[2 θ] + ((pP - qQ) z Sin[2 θ])/Sqrt[ξ]]^2 + Abs[1/ξ^4 (4 z^3 + z ξ^2 (pP + qQ +
```

$$4\left(x^2+y^2\right)\xi\big)-(pP-qQ)\,z\,\xi^2\,\mathrm{Cos}[2\,\theta]+(pP-qQ)\,y\,\xi^{7/2}\,\mathrm{Sin}[2\,\theta]\big)\Big]^2\Big)^2\Big)$$

$$\varkappa\mathrm{MFunc}[x\_,\,y\_,\,z\_]:=\Bigg(-\xi^8\Bigg(\mathrm{Abs}\Big[\frac{4\,x\,z^2}{\xi}+2\,x\,\xi\left(pP+2\left(x^2+y^2\right)\xi\right)\Big]^2+$$
$$\mathrm{Abs}\Big[\frac{4\,y\,z^2}{\xi}+y\,\xi\left(pP+qQ+4\left(x^2+y^2\right)\xi\right)+(pP-qQ)\,y\,\xi\,\mathrm{Cos}[2\,\theta]+$$
$$\frac{(pP-qQ)\,z\,\mathrm{Sin}[2\,\theta]}{\sqrt{\xi}}\Big]^2+\mathrm{Abs}\Big[\frac{1}{\xi^4}\Big(4\,z^3+z\,\xi^2\left(pP+qQ+4\left(x^2+y^2\right)\xi\right)-$$
$$(pP-qQ)\,z\,\xi^2\,\mathrm{Cos}[2\,\theta]+(pP-qQ)\,y\,\xi^{7/2}\,\mathrm{Sin}[2\,\theta]\Big)\Big]^2\Bigg)$$
$$\Big(4\,z^2\left(3+2\,\xi^3\right)+\xi^2\left(pP+3\,pP\,\xi^3+qQ\left(1+\xi^3\right)+4\left(x^2+y^2\right)\xi\left(1+4\,\xi^3\right)\right)+$$
$$(pP-qQ)\,\xi^2\left(-1+\xi^3\right)\mathrm{Cos}[2\,\theta]\Big)+$$
$$8\,x^2\,\xi^6\left(2\,z^2+\xi^2\left(pP+2\left(x^2+y^2\right)\xi\right)\right)\times\Big(8\,z^4+2\,pP\,z^2\,\xi^2+2\,qQ\,z^2\,\xi^2+8\,x^2\,z^2\,\xi^3+$$
$$8\,y^2\,z^2\,\xi^3+4\,z^4\,\xi^3+4\,pP\,z^2\,\xi^5+16\,x^2\,z^2\,\xi^6+16\,y^2\,z^2\,\xi^6+pP^2\,\xi^7+$$
$$8\,pP\,x^2\,\xi^8+6\,pP\,y^2\,\xi^8+2\,qQ\,y^2\,\xi^8+12\,x^4\,\xi^9+24\,x^2\,y^2\,\xi^9+12\,y^4\,\xi^9+$$
$$2\,(pP-qQ)\,\xi^2\left(-z^2+y^2\,\xi^6\right)\mathrm{Cos}[2\,\theta]+2\,(pP-qQ)\,y\,z\,\xi^{7/2}\left(1+\xi^3\right)\mathrm{Sin}[2\,\theta]\Big)+$$
$$\Big(4\,z^3+z\,\xi^2\left(pP+qQ+4\left(x^2+y^2\right)\xi\right)-(pP-qQ)\,z\,\xi^2\,\mathrm{Cos}[2\,\theta]+(pP-qQ)\,y\,\xi^{7/2}\,\mathrm{Sin}[2\,\theta]\Big)\times$$
$$\Big(16\,x^2\,z\,\xi^6\left(2\,z^2+\xi^2\left(pP+2\left(x^2+y^2\right)\xi\right)\right)+$$
$$\xi^6\left(8\,y\,z+(pP-qQ)\sqrt{\xi}\,\mathrm{Sin}[2\,\theta]\right)\times\Big(4\,y\,z^2+y\,\xi^2\left(pP+qQ+4\left(x^2+y^2\right)\xi\right)+$$
$$(pP-qQ)\,y\,\xi^2\,\mathrm{Cos}[2\,\theta]+(pP-qQ)\,z\sqrt{\xi}\,\mathrm{Sin}[2\,\theta]\Big)+$$
$$\left(12\,z^2+\xi^2\left(pP+qQ+4\left(x^2+y^2\right)\xi\right)-(pP-qQ)\,\xi^2\,\mathrm{Cos}[2\,\theta]\right)\times\Big(4\,z^3+z\,\xi^2$$
$$\left(pP+qQ+4\left(x^2+y^2\right)\xi\right)-(pP-qQ)\,z\,\xi^2\,\mathrm{Cos}[2\,\theta]+(pP-qQ)\,y\,\xi^{7/2}\,\mathrm{Sin}[2\,\theta]\Big)\Big)+$$
$$\xi^6\Big(4\,y\,z^2+y\,\xi^2\left(pP+qQ+4\left(x^2+y^2\right)\xi\right)+(pP-qQ)\,y\,\xi^2\,\mathrm{Cos}[2\,\theta]+$$
$$(pP-qQ)\,z\sqrt{\xi}\,\mathrm{Sin}[2\,\theta]\Big)\times\Big(16\,x^2\,y\,\xi^6\left(2\,z^2+\xi^2\left(pP+2\left(x^2+y^2\right)\xi\right)\right)+$$
$$\xi^3\left(4\,z^2+\xi^2\left(pP+qQ+4\left(x^2+3\,y^2\right)\xi\right)+(pP-qQ)\,\xi^2\,\mathrm{Cos}[2\,\theta]\right)\times\Big(4\,y\,z^2+y\,\xi^2$$
$$\left(pP+qQ+4\left(x^2+y^2\right)\xi\right)+(pP-qQ)\,y\,\xi^2\,\mathrm{Cos}[2\,\theta]+(pP-qQ)\,z\sqrt{\xi}\,\mathrm{Sin}[2\,\theta]\Big)+$$
$$\left(8\,y\,z+(pP-qQ)\sqrt{\xi}\,\mathrm{Sin}[2\,\theta]\right)\times\Big(4\,z^3+z\,\xi^2\left(pP+qQ+4\left(x^2+y^2\right)\xi\right)-$$
$$(pP-qQ)\,z\,\xi^2\,\mathrm{Cos}[2\,\theta]+(pP-qQ)\,y\,\xi^{7/2}\,\mathrm{Sin}[2\,\theta]\Big)\Big)\Bigg)\Big/$$
$$\Bigg(2\,\xi^{12}\Bigg(\mathrm{Abs}\Big[\frac{4\,x\,z^2}{\xi}+2\,x\,\xi\left(pP+2\left(x^2+y^2\right)\xi\right)\Big]^2+\mathrm{Abs}\Big[\frac{4\,y\,z^2}{\xi}+y\,\xi\left(pP+qQ+4\left(x^2+y^2\right)\xi\right)+$$
$$(pP-qQ)\,y\,\xi\,\mathrm{Cos}[2\,\theta]+\frac{(pP-qQ)\,z\,\mathrm{Sin}[2\,\theta]}{\sqrt{\xi}}\Big]^2+\mathrm{Abs}\Big[\frac{1}{\xi^4}\Big(4\,z^3+z\,\xi^2\big(pP+qQ+$$
$$4\left(x^2+y^2\right)\xi\big)-(pP-qQ)\,z\,\xi^2\,\mathrm{Cos}[2\,\theta]+(pP-qQ)\,y\,\xi^{7/2}\,\mathrm{Sin}[2\,\theta]\Big)\Big]^2\Bigg)^{3/2}\Bigg)$$

In[]:= Clear[d, b, h, pP, qQ, rR, ξ, θ];

d = 8.0; (* Main diameter of the biconcave disc *)
b = 1; (* Thickness of the biconcave disc at the centre *)
h = 2.12; (* Maximum thickness of the biconcave disc out near the rim... like the width of a car tyre *)

$$pP = -\frac{d^2}{2} + \frac{h^2}{2}\left(\frac{d^2}{b^2} - 1\right) - \frac{h^2}{2}\left(\frac{d^2}{b^2} - 1\right)\left(1 - \frac{b^2}{h^2}\right)^{\frac{1}{2}};$$

(* Coefficient of the $x^2$+ $y^2$ term *)

$$qQ = \frac{d^2}{b^2} pP + \frac{b^2}{4}\left(\frac{d^4}{b^4} - 1\right); \quad \text{(* Coefficient of the } z^2 \text{ term *)}$$

$$rR = -\frac{d^2}{4} pP - \frac{d^4}{16}; \quad \text{(* The constant term *)}$$

tensorRot = {{1, 0, 0}, {0, Cos[θ], -Sin[θ]}, {0, Sin[θ], Cos[θ]}};
tensorStretch = $\left\{\left\{1/\sqrt{\xi}, 0, 0\right\}, \left\{0, 1/\sqrt{\xi}, 0\right\}, \{0, 0, \xi\}\right\}$;
θ = π / 4;
ξ = 1.0;

trf = InverseFunction[AffineTransform[tensorStretch.tensorRot]];

rbc0 = ImplicitRegion$\left[\left(x^2 + y^2 + z^2\right)^2 + pP\left(x^2 + y^2\right) + qQ\, z^2 + rR < 0\right.$ /.
Thread[{x, y, z} → trf[{x, y, z}]], {{x, -7, 7}, {y, -7, 7}, {z, -7, 7}}];

bmr0 = BoundaryDiscretizeRegion[rbc0, MaxCellMeasure → 0.2, AspectRatio → Automatic, MeshCellStyle → Red] (* Note the mesh size set to 0.2 to give a computationally reasonable number of triangles! *)

Out[ ]=
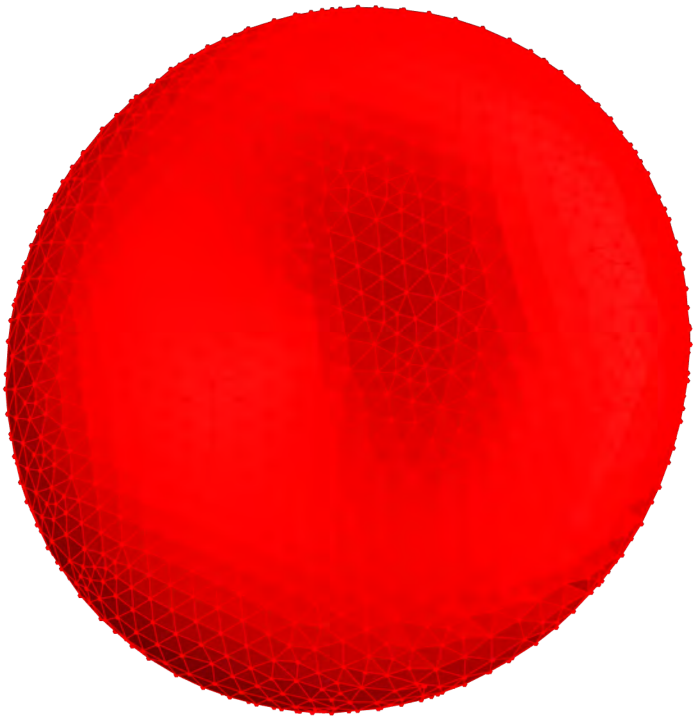

In[ ]:=
```
{RegionMeasure[bmr0], RegionMeasure[RegionBoundary[bmr0]]}
RBCArea = RegionMeasure[RegionBoundary[bmr0]]
meshCoords = MeshCoordinates[bmr0] ;
(* The mesh coordinates come from the boundary discretized graphics values *)
meshTriangles = MeshPrimitives[bmr0, 2] ;
(* The list of mesh triangles is derived from the
 BoundaryDiscretizeRegion[rbc0] of the ListContourPlot3D function *)
```

Out[ ]= {85.4897, 127.685}

Out[ ]= 127.685

In[•]:=
```
l3 = Length[meshTriangles]
l4 = Length[meshCoords]
gphD1 = Graphics3D[{Red, meshTriangles}]
 (* The plot of the mesh triangles simply uses Graphics3D *)
```

Out[•]= 3596

Out[•]= 1800

Out[•]=

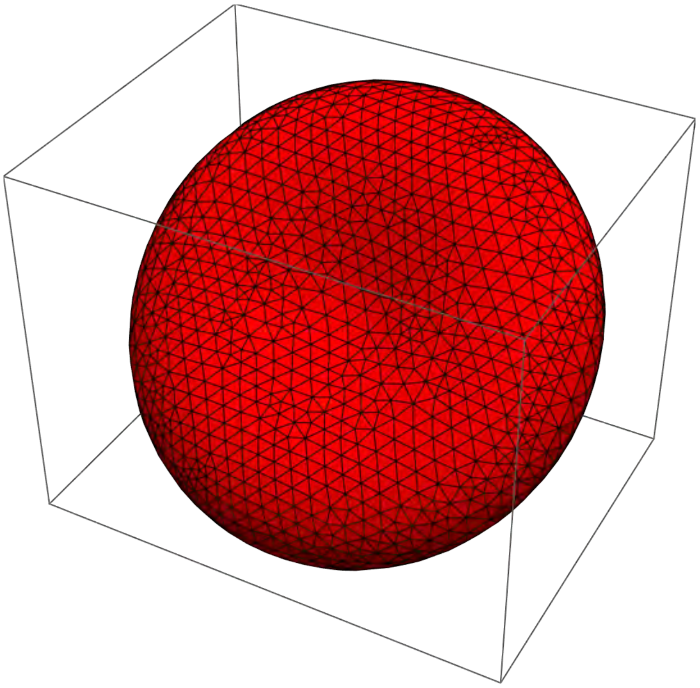

## Example of the mesh (vertex) coordinates for triangle #1

In[•]:=
```
v1 = meshTriangles⟦1⟧⟦1⟧⟦1⟧
v2 = meshTriangles⟦1⟧⟦1⟧⟦2⟧
v3 = meshTriangles⟦1⟧⟦1⟧⟦3⟧
```

Out[•]= {1.77175, 0.0260596, -1.4034}

Out[•]= {1.95489, 0.212637, -1.2314}

Out[•]= {2.12257, 0.0608511, -1.42547}

## Colour code  from smallest to largest values of curvature

In[•]:=
```
colourList = {{1, 0, 0}, {1, 0.5, 0}, {1, 0.7, 0.2}, {1, 1, 0}, {0, 1, 0},
   {0, 0.5, 0}, {0, 0.5, 0.7}, {0.2, 0.4, 1}, {0.6, 0.3, 1}, {0.8, 0.2, 1}};
plotFunc := Graphics3D[{FaceForm[RGBColor[#1]], PolyhedronData[
     "JabulaniPolyhedron", "Polygons"]}, Boxed → False, ImageSize → 60] &
colourKeyPlot = plotFunc /@ colourList // Row
```

Out[•]=

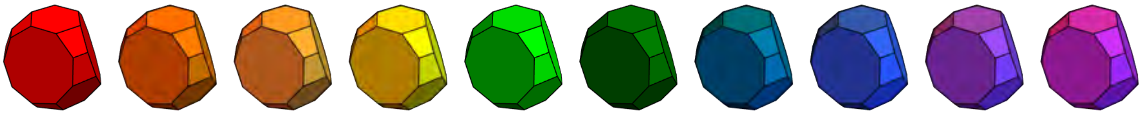

## Get the list of centroids for the l3 triangles and obtain the list of curvatures

from which to extract the minimum and maximum value...and extend this to obtaining the average of the values of the curvatures at each of the three vertices...using triangle 1 as an example to test things out

```
In[•]:= j = 1;

v1 = meshTriangles[[j]][[1]][[1]]
(* meshTriangles[[j]][[1]] is the first element of the triangle list,
which is the three triple corrdinates  so
 meshTriangles[[j]][[1]][[1]] is the first vertex (triple-corrdinate) *)
v2 = meshTriangles[[j]][[1]][[2]] (* meshTriangles[[j]][[1]] is the first element
 of the triangle list, which is the three triple corrdinates so
 meshTriangles[[j]][[1]][[2]] is the second vertex (triple-corrdinate) *)
v3 = meshTriangles[[j]][[1]][[3]] (* meshTriangles[[j]][[1]] is the first element
 of the triangle list, which is the three triple corrdinates so
 meshTriangles[[j]][[1]][[3]] is the third vertex (triple-corrdinate) *)
centroid = (v1 + v2 + v3) / 3; (* Formula for the centroid of
 a triangle whose three vertex coordinates are known *)
area = (1 / 2) Cross[(v2 - v1), (v3 - v1)] // Norm;
(* Formula for the area of a triangle:
  half the normal of the cross product of the vectors of two of the sides *)

c1 = κGFunc[centroid[[1]], centroid[[2]], centroid[[3]]];
(* Apply the Gaussian Curvature function with the three coordinates *)
c2 = κMFunc[centroid[[1]], centroid[[2]], centroid[[3]]];
(* Apply the Mean Curvature function with the three coordinates *)

(* Apply the Gaussian and Mean Curvature formulae to the three vertices *)
v1GC = κGFunc[meshTriangles[[j, 1, 1, 1]],
   meshTriangles[[j, 1, 1, 2]], meshTriangles[[j, 1, 1, 3]]];
v1MC = κMFunc[meshTriangles[[j, 1, 1, 1]], meshTriangles[[j, 1, 1, 2]],
   meshTriangles[[j, 1, 1, 3]]];
v1k1 = v1MC + √(v1MC^2 - v1GC) // Re;
v1k2 = v1MC - √(v1MC^2 - v1GC) // Re;

v2GC = κGFunc[meshTriangles[[j, 1, 2, 1]],
   meshTriangles[[j, 1, 2, 2]], meshTriangles[[j, 1, 2, 3]]];
v2MC = κMFunc[meshTriangles[[j, 1, 2, 1]], meshTriangles[[j, 1, 2, 2]],
   meshTriangles[[j, 1, 2, 3]]];
v2k1 = v2MC + √(v2MC^2 - v2GC) // Re;
v2k2 = v2MC - √(v2MC^2 - v2GC) // Re;

v3GC = κGFunc[meshTriangles[[j, 1, 3, 1]],
   meshTriangles[[j, 1, 3, 2]], meshTriangles[[j, 1, 3, 3]]];
v3MC = κMFunc[meshTriangles[[j, 1, 3, 1]], meshTriangles[[j, 1, 3, 2]],
```

```
   meshTriangles[[j, 1, 3, 3]]];
v3k1 = v3MC + Sqrt[v3MC^2 - v3GC] // Re;
v3k2 = v3MC - Sqrt[v3MC^2 - v3GC] // Re;

aveGC = (v1GC + v2GC + v3GC) / 3
aveMC = (v1MC + v2MC + v3MC) / 3
avek1 = (v1k1 + v2k1 + v3k1) / 3
avek2 = (v1k2 + v2k2 + v3k2) / 3
```

```
Out[•]= {1.77175, 0.0260596, -1.4034}
Out[•]= {1.95489, 0.212637, -1.2314}
Out[•]= {2.12257, 0.0608511, -1.42547}
Out[•]= -0.0184404
Out[•]= -0.114505
Out[•]= 0.065066
Out[•]= -0.294077
```

```
In[•]:=
Clear[j];

triangleProperties = {};
For[j = 1, j ≤ l3, j++,
  v1 = meshTriangles[[j]][[1]][[1]];
  (* meshTriangles[[j]][[1]] is the first element of the triangle list,
  which is the three triple corrdinates  so meshTriangles[[j]][[1]][[1]]
   is the first vertex (triple-corrdinate) *)
  v2 = meshTriangles[[j]][[1]][[2]] ;
  (* meshTriangles[[j]][[1]] is the first element of the triangle list,
  which is the three triple corrdinates so meshTriangles[[j]][[1]][[2]]
   is the second vertex (triple-corrdinate) *)
  v3 = meshTriangles[[j]][[1]][[3]]; (* meshTriangles[[j]][[1]] is the first
   element of the triangle list, which is the three triple corrdinates
   so meshTriangles[[j]][[1]][[3]] is the third vertex (triple-corrdinate) *)
  centroid = (v1 + v2 + v3) / 3;  (* Formula for the centroid of
   a triangle whose three vertex coordinates are known *)
  area = (1 / 2) Cross[(v2 - v1), (v3 - v1)] // Norm;
  (* Formula for the area of a triangle: half the normal of
     the cross product of the vectors of two of the sides *)

  c1 = κGFunc[centroid[[1]], centroid[[2]], centroid[[3]]];
  (* Apply the Gaussian Curvature function with the three coordinates *)
  c2 = κMFunc[centroid[[1]], centroid[[2]], centroid[[3]]];
  (* Apply the Mean Curvature function with the three coordinates *)
```

```
  (* Apply the Gaussian and Mean Curvature formulae to the three vertices *)
  v1GC = κGFunc[meshTriangles〚j, 1, 1, 1〛,
    meshTriangles〚j, 1, 1, 2〛, meshTriangles〚j, 1, 1, 3〛];
  v1MC = κMFunc[meshTriangles〚j, 1, 1, 1〛, meshTriangles〚j, 1, 1, 2〛,
    meshTriangles〚j, 1, 1, 3〛];
  v1k1 = v1MC + Sqrt[v1MC^2 - v1GC];
  v1k2 = v1MC - Sqrt[v1MC^2 - v1GC];

  v2GC = κGFunc[meshTriangles〚j, 1, 2, 1〛,
    meshTriangles〚j, 1, 2, 2〛, meshTriangles〚j, 1, 2, 3〛];
  v2MC = κMFunc[meshTriangles〚j, 1, 2, 1〛, meshTriangles〚j, 1, 2, 2〛,
    meshTriangles〚j, 1, 2, 3〛];
  v2k1 = v2MC + Sqrt[v2MC^2 - v2GC];
  v2k2 = v2MC - Sqrt[v2MC^2 - v2GC];

  v3GC = κGFunc[meshTriangles〚j, 1, 3, 1〛,
    meshTriangles〚j, 1, 3, 2〛, meshTriangles〚j, 1, 3, 3〛];
  v3MC = κMFunc[meshTriangles〚j, 1, 3, 1〛, meshTriangles〚j, 1, 3, 2〛,
    meshTriangles〚j, 1, 3, 3〛];
  v3k1 = v3MC + Sqrt[v3MC^2 - v3GC];
  v3k2 = v3MC - Sqrt[v3MC^2 - v3GC];

  aveGC = (v1GC + v2GC + v3GC) / 3;
  aveMC = (v1MC + v2MC + v3MC) / 3;
  avek1 = (v1k1 + v2k1 + v3k1) / 3;
  avek2 = (v1k2 + v2k2 + v3k2) / 3;
  triangle = {centroid, area, c1, c2, aveGC, aveMC, avek1, avek2};
  triangleProperties = AppendTo[triangleProperties, triangle];
 ];
```

In[∘]:=
```
triangleProperties〚1〛 (* OK it works *)
triangleProperties〚1000〛
```

Out[∘]= {{1.94974, 0.0998494, -1.35342}, 0.0439835, -0.0192437, -0.113899, -0.0184404, -0.114505, 0.065066, -0.294077}

Out[∘]= {{-1.9946, -2.4839, -1.16012}, 0.0193491, 0.0705008, -0.375494, 0.0711604, -0.375352, -0.110069, -0.640635}

## Find the maximum and minimum values of the four sorts of curvature so that the whole domain (span) can be divided into 10 sub-domains

In[•]:=
```
gaussCurvListMin = Min@Table[triangleProperties⟦i⟧ [[5]], {i, 1, l3}]
(* Index 5 denotes the vertex-averaged Gaussian Curvature *)
gaussCurvListMax = Max@Table[triangleProperties⟦i⟧ [[5]], {i, 1, l3}]
meanCurvListMin = Min@Table[triangleProperties⟦i⟧ [[6]], {i, 1, l3}]
(* Index 6 denotes the vertex-averaged Mean Curvature *)
meanCurvListMax = Max@Table[triangleProperties⟦i⟧ [[6]], {i, 1, l3}]
k1CurvListMin = Min@Table[triangleProperties⟦i⟧ [[7]] // Re, {i, 1, l3}]
(* Index 7 denotes the vertex-averaged k1 Principal Curvature-1 *)
k1CurvListMax = Max@Table[triangleProperties⟦i⟧ [[7]] // Re, {i, 1, l3}]
k2CurvListMin = Min@Table[triangleProperties⟦i⟧ [[8]] // Re, {i, 1, l3}]
(* Index 7 denotes the vertex-averaged k1 Principal Curvature-1 *)
k2CurvListMax = Max@Table[triangleProperties⟦i⟧ [[8]] // Re, {i, 1, l3}]
```

Out[•]= -0.0279632

Out[•]= 0.314024

Out[•]= -0.713572

Out[•]= 0.560575

Out[•]= -0.249347

Out[•]= 0.589304

Out[•]= -1.1778

Out[•]= 0.531847

## Based on the maximum and minimum values of the lists of curvatures, do a For loop and make a specified number of “bins”, say 10

In[•]:=
```
bin[1] = {};
bin[2] = {};
bin[3] = {};
bin[4] = {};
bin[5] = {};
bin[6] = {};
bin[7] = {};
bin[8] = {};
bin[9] = {};
bin[10] = {};

minG = gaussCurvListMin;
maxG = gaussCurvListMax;
delta = (maxG - minG) / 10;

(* This sorts the triangles according to their Gaussian Curvatures *)
For[j = 1, j ≤ 10, j++,
  For[i = 1, i ≤ l3, i++,
    a = triangleProperties⟦i⟧⟦2⟧;  (* Area *)
    d = triangleProperties⟦i⟧⟦5⟧;  (* Gaussian Curvature *)
    If[d ≥ (minG + (j - 1) delta) && d < (minG + j delta),
     AppendTo[bin[j], {i, d, a}]];
   ];
 ];
```

In[•]:=
```
bin[1]⟦1⟧⟦1⟧(* The triangle index *)
bin[1]⟦1⟧⟦2⟧(* The Gaussian Curvature *)
```

Out[•]= 1

Out[•]= -0.0184404

## Plot each of the sets of triangles in the lists in the respective bins...show only two here to save space

In[•]:=
```
Clear[gphG];
outGraphs = For[i = 1, i ≤ 10, i++,
   indexNos = Table[Flatten[Transpose[bin[i]]⟦1⟧, 1]⟦j⟧, {j, 1, Length@bin[i]}];
   binSubSet = meshTriangles⟦#⟧ & /@ indexNos;
   meanBin[i] = Total[Flatten[Transpose[bin[i]]⟦2⟧, 1]] / Length[bin[i]];
   gphG[i] = Graphics3D[{RGBColor[colourList⟦i⟧], binSubSet}];
  ];
```

In[ ]:= gphG[1]
gphG[6]

Out[ ]= 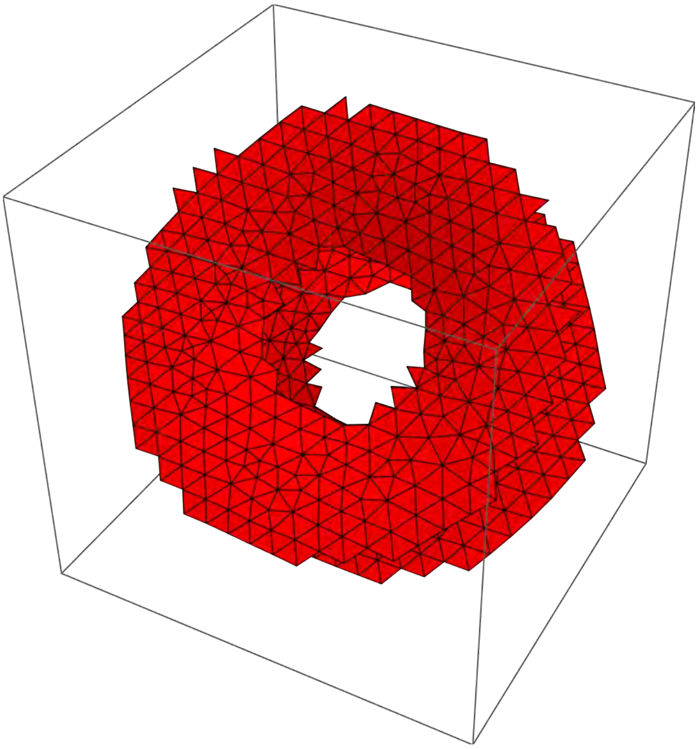

Out[ ]= 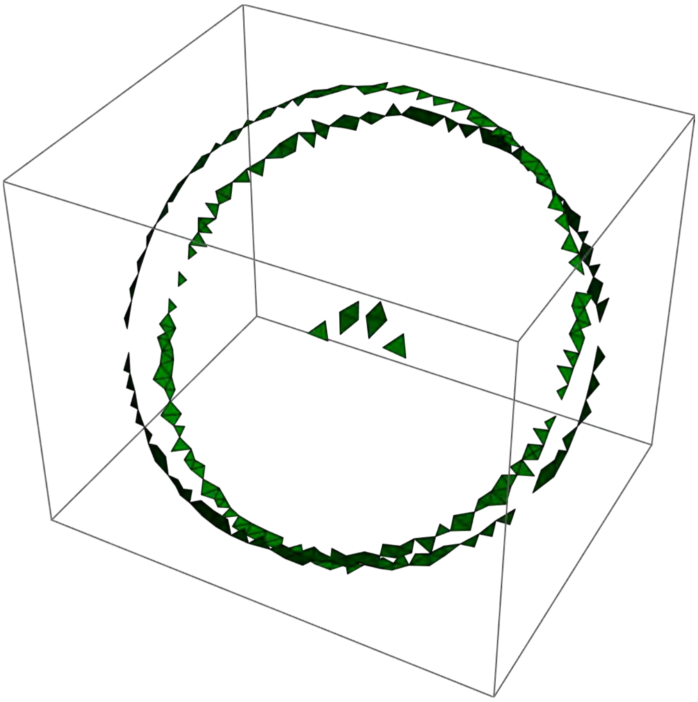

## Show the full superposition of all 10 bins of triangles

```
In[•]:= Show[{gphG[1], gphG[2], gphG[3], gphG[4],
          gphG[5], gphG[6], gphG[7], gphG[8], gphG[9], gphG[10]}]
```

Out[•]=

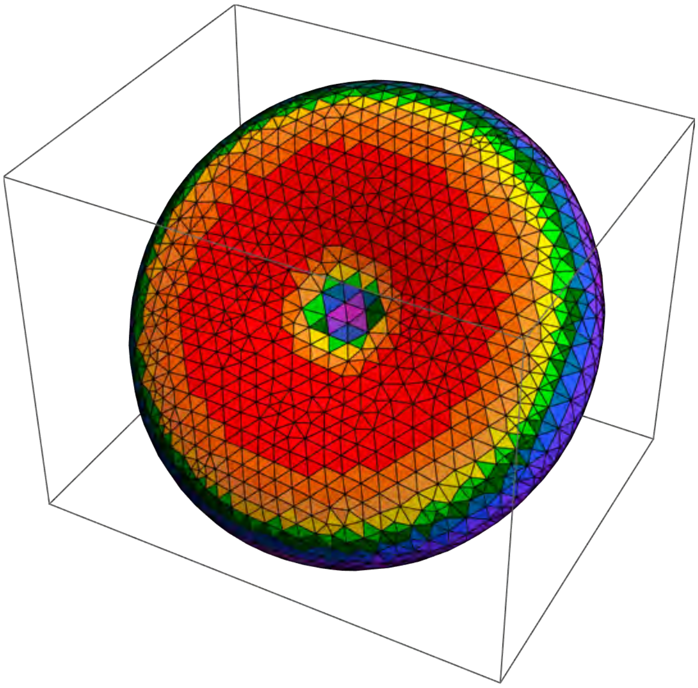

## We seek the weighted mean value of the curvature in each of the 10 bins for the Gaussian curvature...use triangleProperties = {centroid,area,c1,c2,aveGC,aveMC}... and bin {j,aveGc,area} only contains the index, the curvature and the area

```
In[•]:= bin[1]⟦3⟧⟦2⟧

Out[•]= -0.0186401

In[•]:= bin[2]; (* Have a look at the contents of one of the bins to
         ensure things are working, then switch off output with a ";" *)

In[•]:= histoUncorrGauss = Table[Length[bin[i]], {i, 1, 10}]
         (* Give the number of entries in each bin *)

Out[•]= {1031, 406, 314, 316, 289, 233, 208, 206, 290, 302}

In[•]:= relativeBinAreasGauss =
         Table[Total@Table[bin[i]⟦j⟧⟦3⟧, {i, 1, 10}, {j, 1, Length@bin[i]}]⟦i⟧,
           {i, 1, 10}] / RBCArea (* Give the area of the RBC that has the
            curvature in the curvature-value domain of the particular bin *)

Out[•]= {0.299478, 0.126476, 0.092749, 0.0739158, 0.066106,
         0.0573364, 0.058664, 0.0649923, 0.0891477, 0.0706899}
```

```
In[∘]:= binAreasGauss = Table[
          Total@Table[bin[i]〚j〛〚3〛, {i, 1, 10}, {j, 1, Length@bin[i]}]〚i〛, {i, 1, 10}]
        (* This is the raw value of the area of the RBC that has the
          curvature in the curvature-value domain of the particular bin *)
        histoUncorrGauss // Total

Out[∘]= {38.2389, 16.1491, 11.8427, 9.43795,
         8.44076, 7.32101, 7.49052, 8.29856, 11.3828, 9.02606}

Out[∘]= 3595

In[∘]:= wtAveBinCurvGauss =
         Table[Total[Table[bin[j]〚i〛〚2〛 × bin[j]〚i〛〚3〛, {i, 1, Length@bin[j]}]] /
           binAreasGauss〚j〛, {j, 1, 10}]
        (* The sum of the curvature multiplied by the area of the
            triangle divided by the total area of all the triangles...
          thus giving the area-weighted average of the curvature *)
        toPlotBinVsCurvGauss = Transpose[{wtAveBinCurvGauss, relativeBinAreasGauss}]

Out[∘]= {-0.0161238, 0.0225378, 0.0569785, 0.091353,
         0.125812, 0.159798, 0.194259, 0.228055, 0.264181, 0.287185}

In[∘]:= binAreasGauss.(wtAveBinCurvGauss)

Out[∘]= 12.4631

In[∘]:=

        gphG1A = ListPlot[toPlotBinVsCurvGauss, Joined → True,
           AxesOrigin → {0, 0}, PlotStyle → {Red, Thick}, PlotRange → All];
        gphG1B = ListPlot[toPlotBinVsCurvGauss, Joined → False, AxesOrigin → {0, 0},
           PlotStyle → {PointSize[0.03], Red}, PlotRange → All];
        gphD2 = Show[{gphG1A, gphG1B}]
```

Out[∘]=

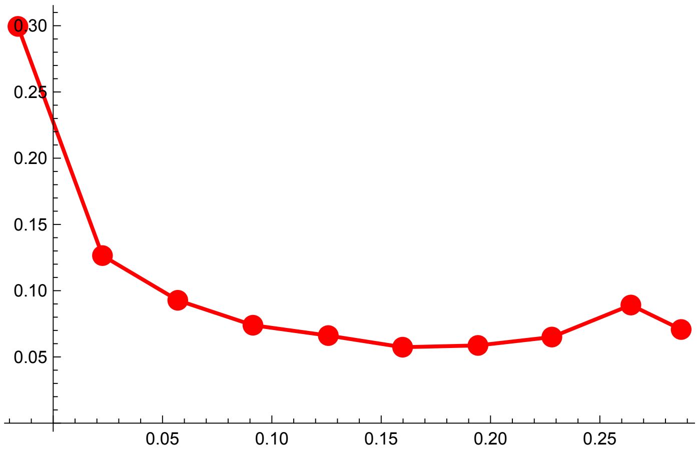


The other three curvatures are colour-mapped onto the RBC and graphed in exactly the same was as described above for the Gaussian Curvature.
