# Supplementary material for: Surface model of the human red blood cell simulating changes in membrane curvature under strain
Source: Sci Rep. 2021 Jul 1;11:13712. doi: 10.1038/s41598-021-92699-7 (PMC8249411; doi:10.1038/s41598-021-92699-7)

Notebook 7 Calculations for Figure 9

Differences in mesh-triangle areas, and curvatures, between stretched and relaxed RBCs

First define the Gaussian and Mean Curvatures

In[•]:=
```
Clear[x, y, z, ξ, θ, pP, qQ, rR];
κGFunc[x_, y_, z_] :=
 (8 × (2 z^2 + ξ^2 (pP + 2 (x^2 + y^2) ξ)) × (16 z^8 + 12 pP z^6 ξ^2 + 12 qQ z^6 ξ^2 + 64 x^2 z^6 ξ^3 +
      64 y^2 z^6 ξ^3 + 3 pP^2 z^4 ξ^4 + 6 pP qQ z^4 ξ^4 + 3 qQ^2 z^4 ξ^4 + 40 pP x^2 z^4 ξ^5 + 32 qQ x^2 z^4 ξ^5 +
      36 pP y^2 z^4 ξ^5 + 36 qQ y^2 z^4 ξ^5 + pP^2 qQ z^2 ξ^6 + pP qQ^2 z^2 ξ^6 + 96 x^4 z^4 ξ^6 +
      192 x^2 y^2 z^4 ξ^6 + 96 y^4 z^4 ξ^6 + 10 pP^2 x^2 z^2 ξ^7 + 8 pP qQ x^2 z^2 ξ^7 + 6 qQ^2 x^2 z^2 ξ^7 +
      6 pP^2 y^2 z^2 ξ^7 + 12 pP qQ y^2 z^2 ξ^7 + 6 qQ^2 y^2 z^2 ξ^7 + 44 pP x^4 z^2 ξ^8 + 28 qQ x^4 z^2 ξ^8 +
      80 pP x^2 y^2 z^2 ξ^8 + 64 qQ x^2 y^2 z^2 ξ^8 + 36 pP y^4 z^2 ξ^8 + 36 qQ y^4 z^2 ξ^8 + 2 pP^2 qQ x^2 ξ^9 +
      pP^2 qQ y^2 ξ^9 + pP qQ^2 y^2 ξ^9 + 64 x^6 z^2 ξ^9 + 192 x^4 y^2 z^2 ξ^9 + 192 x^2 y^4 z^2 ξ^9 +
      64 y^6 z^2 ξ^9 + 4 pP^2 x^4 ξ^10 + 8 pP qQ x^4 ξ^10 + 10 pP^2 x^2 y^2 ξ^10 + 8 pP qQ x^2 y^2 ξ^10 +
      6 qQ^2 x^2 y^2 ξ^10 + 3 pP^2 y^4 ξ^10 + 6 pP qQ y^4 ξ^10 + 3 qQ^2 y^4 ξ^10 + 16 pP x^6 ξ^11 +
      8 qQ x^6 ξ^11 + 44 pP x^4 y^2 ξ^11 + 28 qQ x^4 y^2 ξ^11 + 40 pP x^2 y^4 ξ^11 + 32 qQ x^2 y^4 ξ^11 +
      12 pP y^6 ξ^11 + 12 qQ y^6 ξ^11 + 16 x^8 ξ^12 + 64 x^6 y^2 ξ^12 + 96 x^4 y^4 ξ^12 + 64 x^2 y^6 ξ^12 +
      16 y^8 ξ^12 + (pP - qQ) ξ^2 (-z^2 + y^2 ξ^3) (4 z^4 + 2 z^2 ξ^2 (pP + qQ + 4 (x^2 + y^2) ξ) +
         ξ^4 (pP (qQ + 2 (-x^2 + y^2) ξ) + 2 ξ (qQ (3 x^2 + y^2) + 2 (x^2 + y^2)^2 ξ))) Cos[2 θ] -
      (pP - qQ)^2 ξ^4 (z^4 - 6 y^2 z^2 ξ^3 + y^4 ξ^6) Cos[4 θ] + 8 pP y z^5 ξ^(7/2) Sin[2 θ] -
      8 qQ y z^5 ξ^(7/2) Sin[2 θ] + 4 pP^2 y z^3 ξ^(11/2) Sin[2 θ] - 4 qQ^2 y z^3 ξ^(11/2) Sin[2 θ] +
      16 pP x^2 y z^3 ξ^(13/2) Sin[2 θ] - 16 qQ x^2 y z^3 ξ^(13/2) Sin[2 θ] + 16 pP y^3 z^3 ξ^(13/2) Sin[2 θ] -
      16 qQ y^3 z^3 ξ^(13/2) Sin[2 θ] + 2 pP^2 qQ y z ξ^(15/2) Sin[2 θ] - 2 pP qQ^2 y z ξ^(15/2) Sin[2 θ] -
      4 pP^2 x^2 y z ξ^(17/2) Sin[2 θ] + 16 pP qQ x^2 y z ξ^(17/2) Sin[2 θ] -
      12 qQ^2 x^2 y z ξ^(17/2) Sin[2 θ] + 4 pP^2 y^3 z ξ^(17/2) Sin[2 θ] - 4 qQ^2 y^3 z ξ^(17/2) Sin[2 θ] +
      8 pP x^4 y z ξ^(19/2) Sin[2 θ] - 8 qQ x^4 y z ξ^(19/2) Sin[2 θ] + 16 pP x^2 y^3 z ξ^(19/2) Sin[2 θ] -
      16 qQ x^2 y^3 z ξ^(19/2) Sin[2 θ] + 8 pP y^5 z ξ^(19/2) Sin[2 θ] - 8 qQ y^5 z ξ^(19/2) Sin[2 θ] +
      4 pP^2 y z^3 ξ^(11/2) Sin[4 θ] - 8 pP qQ y z^3 ξ^(11/2) Sin[4 θ] + 4 qQ^2 y z^3 ξ^(11/2) Sin[4 θ] -
      4 pP^2 y^3 z ξ^(17/2) Sin[4 θ] + 8 pP qQ y^3 z ξ^(17/2) Sin[4 θ] - 4 qQ^2 y^3 z ξ^(17/2) Sin[4 θ])) /
  (ξ^10 (Abs[(4 x z^2)/ξ + 2 x ξ (pP + 2 (x^2 + y^2) ξ)]^2 + Abs[(4 y z^2)/ξ + y ξ (pP + qQ + 4 (x^2 + y^2) ξ) +
         (pP - qQ) y ξ Cos[2 θ] + ((pP - qQ) z Sin[2 θ])/Sqrt[ξ]]^2 + Abs[1/ξ^4 (4 z^3 + z ξ^2 (pP + qQ +
              4 (x^2 + y^2) ξ) - (pP - qQ) z ξ^2 Cos[2 θ] + (pP - qQ) y ξ^(7/2) Sin[2 θ])]^2)^2)
```

```
κMFunc[x_, y_, z_] := (-ξ^8 (Abs[4 x z^2/ξ + 2 x ξ (pP + 2 (x^2 + y^2) ξ)]^2 +
      Abs[4 y z^2/ξ + y ξ (pP + qQ + 4 (x^2 + y^2) ξ) + (pP - qQ) y ξ Cos[2 θ] +
         (pP - qQ) z Sin[2 θ]/Sqrt[ξ]]^2 + Abs[1/ξ^4 (4 z^3 + z ξ^2 (pP + qQ + 4 (x^2 + y^2) ξ) -
           (pP - qQ) z ξ^2 Cos[2 θ] + (pP - qQ) y ξ^(7/2) Sin[2 θ])]^2)
    (4 z^2 (3 + 2 ξ^3) + ξ^2 (pP + 3 pP ξ^3 + qQ (1 + ξ^3) + 4 (x^2 + y^2) ξ (1 + 4 ξ^3)) +
      (pP - qQ) ξ^2 (-1 + ξ^3) Cos[2 θ]) +
   8 x^2 ξ^6 (2 z^2 + ξ^2 (pP + 2 (x^2 + y^2) ξ)) × (8 z^4 + 2 pP z^2 ξ^2 + 2 qQ z^2 ξ^2 + 8 x^2 z^2 ξ^3 +
      8 y^2 z^2 ξ^3 + 4 z^4 ξ^3 + 4 pP z^2 ξ^5 + 16 x^2 z^2 ξ^6 + 16 y^2 z^2 ξ^6 + pP^2 ξ^7 +
      8 pP x^2 ξ^8 + 6 pP y^2 ξ^8 + 2 qQ y^2 ξ^8 + 12 x^4 ξ^9 + 24 x^2 y^2 ξ^9 + 12 y^4 ξ^9 +
      2 (pP - qQ) ξ^2 (-z^2 + y^2 ξ^6) Cos[2 θ] + 2 (pP - qQ) y z ξ^(7/2) (1 + ξ^3) Sin[2 θ]) +
   (4 z^3 + z ξ^2 (pP + qQ + 4 (x^2 + y^2) ξ) - (pP - qQ) z ξ^2 Cos[2 θ] + (pP - qQ) y ξ^(7/2) Sin[2 θ]) ×
    (16 x^2 z ξ^6 (2 z^2 + ξ^2 (pP + 2 (x^2 + y^2) ξ)) +
      ξ^6 (8 y z + (pP - qQ) Sqrt[ξ] Sin[2 θ]) × (4 y z^2 + y ξ^2 (pP + qQ + 4 (x^2 + y^2) ξ) +
         (pP - qQ) y ξ^2 Cos[2 θ] + (pP - qQ) z Sqrt[ξ] Sin[2 θ]) +
      (12 z^2 + ξ^2 (pP + qQ + 4 (x^2 + y^2) ξ) - (pP - qQ) ξ^2 Cos[2 θ]) × (4 z^3 + z ξ^2
          (pP + qQ + 4 (x^2 + y^2) ξ) - (pP - qQ) z ξ^2 Cos[2 θ] + (pP - qQ) y ξ^(7/2) Sin[2 θ])) +
   ξ^6 (4 y z^2 + y ξ^2 (pP + qQ + 4 (x^2 + y^2) ξ) + (pP - qQ) y ξ^2 Cos[2 θ] +
      (pP - qQ) z Sqrt[ξ] Sin[2 θ]) × (16 x^2 y ξ^6 (2 z^2 + ξ^2 (pP + 2 (x^2 + y^2) ξ)) +
      ξ^3 (4 z^2 + ξ^2 (pP + qQ + 4 (x^2 + 3 y^2) ξ) + (pP - qQ) ξ^2 Cos[2 θ]) × (4 y z^2 + y ξ^2
          (pP + qQ + 4 (x^2 + y^2) ξ) + (pP - qQ) y ξ^2 Cos[2 θ] + (pP - qQ) z Sqrt[ξ] Sin[2 θ]) +
      (8 y z + (pP - qQ) Sqrt[ξ] Sin[2 θ]) × (4 z^3 + z ξ^2 (pP + qQ + 4 (x^2 + y^2) ξ) -
         (pP - qQ) z ξ^2 Cos[2 θ] + (pP - qQ) y ξ^(7/2) Sin[2 θ]))) /
  (2 ξ^12 (Abs[4 x z^2/ξ + 2 x ξ (pP + 2 (x^2 + y^2) ξ)]^2 + Abs[4 y z^2/ξ + y ξ (pP + qQ + 4 (x^2 + y^2) ξ) +
          (pP - qQ) y ξ Cos[2 θ] + (pP - qQ) z Sin[2 θ]/Sqrt[ξ]]^2 + Abs[1/ξ^4 (4 z^3 + z ξ^2 (pP + qQ +
               4 (x^2 + y^2) ξ) - (pP - qQ) z ξ^2 Cos[2 θ] + (pP - qQ) y ξ^(7/2) Sin[2 θ])]^2)^(3/2))
```

Define the strain and rotation matrices and their product, and perform some tests. We use tensorStretch ‘dotted’ with tensorRot to transform the coordinates of the vertices of the mesh triangles using tenStr

```
In[•]:= Clear[xxx, yyy, zzz, ξ, θ, tenStr];
        tensorRot = {{1, 0, 0}, {0, Cos[θ], -Sin[θ]}, {0, Sin[θ], Cos[θ]}};
        tensorStretch = {{1/ √ξ , 0, 0}, {0, 1/ √ξ , 0}, {0, 0, ξ}};

        tenStr[ξ_, θ_] := {{1/ √ξ , 0, 0}, {0, 1/ √ξ , 0}, {0, 0, ξ}}.
          {{1, 0, 0}, {0, Cos[θ], -Sin[θ]}, {0, Sin[θ], Cos[θ]}}

        vectorTest = {xxx, yyy, zzz}
        tenStr[ξ, θ].vectorTest

Out[•]= {xxx, yyy, zzz}
```

Out[•]= $\left\{\frac{xxx}{\sqrt{\xi}}, \frac{yyy\ Cos[\theta]}{\sqrt{\xi}} - \frac{zzz\ Sin[\theta]}{\sqrt{\xi}}, zzz\ \xi\ Cos[\theta] + yyy\ \xi\ Sin[\theta]\right\}$

Plot the distributions of maximum and minimum edge lengths and triangle areas etc for an RBC, with less than 121k edges so the processing goes more rapidly

In[◦]:=
```
Clear[d, b, h, pP, qQ, rR, ξ, θ];

d = 8.0;  (* Main diameter of the biconcave disc *)
b = 1;  (* Thickness of the biconcave disc at the centre *)
h = 2.12;  (* Maximum thickness of the biconcave disc out near the rim...
  like the width of a car tyre *)
```

$$pP = -\frac{d^2}{2} + \frac{h^2}{2}\left(\frac{d^2}{b^2} - 1\right) - \frac{h^2}{2}\left(\frac{d^2}{b^2} - 1\right)\left(1 - \frac{b^2}{h^2}\right)^{\frac{1}{2}};$$

(* Coefficient of the x^2+ y^2 term *)

$$qQ = \frac{d^2}{b^2} pP + \frac{b^2}{4}\left(\frac{d^4}{b^4} - 1\right);$$ (* Coefficient of the z^2 term *)

$$rR = -\frac{d^2}{4} pP - \frac{d^4}{16};$$ (* The constant term *)

```
tensorRot := {{1, 0, 0}, {0, Cos[θ], -Sin[θ]}, {0, Sin[θ], Cos[θ]}};
tensorStretch := {{1/ √ξ, 0, 0}, {0, 1/ √ξ, 0}, {0, 0, ξ}};

θ = π / 4 ;
ξ = 1;

trf = InverseFunction[AffineTransform[tensorStretch.tensorRot]];

rbc0 = ImplicitRegion[(x^2 + y^2 + z^2)^2 + pP (x^2 + y^2) + qQ z^2 + rR < 0 /.
    Thread[{x, y, z} → trf[{x, y, z}]], {{x, -7, 7}, {y, -7, 7}, {z, -7, 7}}];

bmr0 = BoundaryDiscretizeRegion[rbc0, MaxCellMeasure → 0.08,
  AspectRatio → Automatic, MeshCellStyle → {{2, All} → Opacity[0.5, Green],
    {1, All} → Black, {0, All} → Directive[PointSize[Small], Black]}]
 (* Note the mesh size set to 0.08 to give a computationally
  reasonable number of triangles! *)
```

Out[ ]=

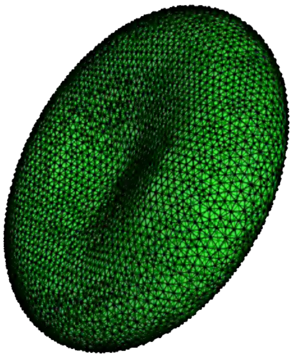

In[ ]:=
```
{RegionMeasure[bmr0], RegionMeasure[RegionBoundary[bmr0]]}
RBCArea = RegionMeasure[RegionBoundary[bmr0]]
meshCoords = MeshCoordinates[bmr0];
(* The mesh coordinates come from the boundary discretized graphics values *)
lengthNumber = 3 Length@meshCoords
```

Out[ ]= {85.7994, 127.894}

Out[ ]= 127.894

Out[ ]= 14 382

In[ ]:=
```
meshTriangles = MeshPrimitives[bmr0, 2] ;
(* The list of mesh triangles is derived from the
 BoundaryDiscretizeRegion[rbc0] of the ImplicitRegion function *)
```

Explore the generation of transformed mesh coordinates and the plotting of the transformed RBC...it works well as shown below

In[ ]:=

```
θ = 0; (* Note that θ = 0 is what is required to
  get the π/4 rotation to work in the next bit of code *)
ξ = 1.75;
gph101 = Graphics3D[
   Triangle /@ Table[meshTriangles⟦j⟧⟦1⟧, {j, 1, Length@meshTriangles}]];
gph102 = Graphics3D[Triangle /@ Table[meshTriangles⟦j⟧⟦1⟧.
      (tensorStretch.tensorRot), {j, 1, Length@meshTriangles}]];
Show[{gph101, gph102}]
```

Out[ ]=

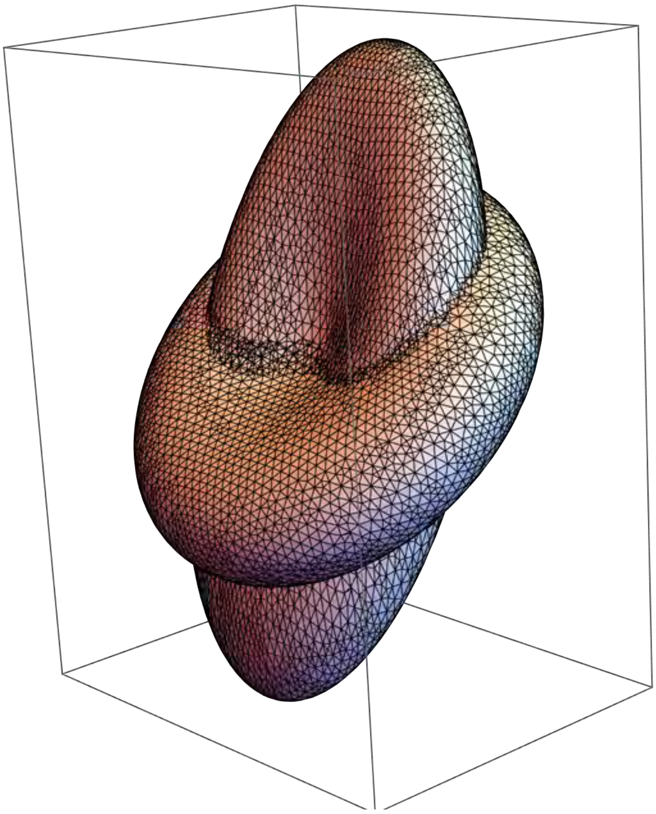

Make the two lists of vertices of all the triangles in the mesh

In[ ]:=

```
meshTrianglesRelaxed = Table[meshTriangles⟦j⟧⟦1⟧, {j, 1, Length@meshTriangles}];
meshTrianglesDistorted = Table[meshTriangles⟦j⟧⟦1⟧.(tensorStretch.tensorRot),
   {j, 1, Length@meshTriangles}];
```

Define the lengths of the files that contain the triangles and the vertex coordinates

In[•]:=
```
l3 = Length[meshTrianglesDistorted]
l4 = Length[meshCoords]
```

Out[•]= 9584

Out[•]= 4794

Obtain the list of centroids for the l3 triangles, curvatures from which to extract the minimum and maximum value, and extend this list to the average of the values of the curvatures at each of the three vertices, the triangle areas, side lengths and extract the minimum and maximum values of the sides of each triangle...using triangle 1 as an example to test the process...worked

In[•]:=
```
meshTrianglesRelaxed⟦1⟧⟦3⟧
meshTrianglesDistorted⟦1⟧⟦3⟧
```

Out[•]= {-3.64824, 1.24253, 1.03082}

Out[•]= {-2.75781, 0.939268, 1.80393}

Cycle through all the l3 triangles and make a list of the triangle properties for later parsing to show various features of the triangularization process

In[•]:=
```
ξ = 1;
θ = π / 2;

trianglePropsRelaxed = {};
(* Meaning "triangle properties" for the relaxed RBC *)
For[j = 1, j ≤ l3, j++,
  v1 = meshTrianglesRelaxed⟦j⟧⟦1⟧;
  (* meshTrianglesRelaxed⟦j⟧ is the jth triangle list,
  which is the three triple corrdinates so meshTriangles⟦j⟧⟦1⟧
   is the first vertex (triple-coordinate) *)
  v2 = meshTrianglesRelaxed⟦j⟧⟦2⟧ ;
  (* ...meshTriangles⟦j⟧⟦2⟧ is the second vertex (triple-coordinate) *)
  v3 = meshTrianglesRelaxed⟦j⟧⟦3⟧;
  (* ...meshTriangles⟦j⟧⟦3⟧ is the third vertex (triple-coordinate) *)

  centroid = (v1 + v2 + v3) / 3;  (* Formula for the centroid
```

```
 of a triangle whose three vertex coordinates are known *)
area = (1 / 2) Cross[(v2 - v1), (v3 - v1)] // Norm;
(* Formula for the area of a triangle: half the normal of
    the cross product of the vectors of two of the sides *)
sideLength1 = Norm[v2 - v1];
sideLength2 = Norm[v3 - v2];
sideLength3 = Norm[v1 - v3];

minSideLengths = Min[sideLength1, sideLength2, sideLength3];
maxSideLengths = Max[sideLength1, sideLength2, sideLength3];

c1 = κGFunc[centroid⟦1⟧, centroid⟦2⟧, centroid⟦3⟧];  (* Apply the Gaussian
 Curvature function with the three coordinates of the centroid *)
c2 = κMFunc[centroid⟦1⟧, centroid⟦2⟧, centroid⟦3⟧]; (* Apply the Mean
 Curvature function with the three coordinates of the centroid *)

(* Apply the Gaussian and Mean Curvature formulae to the three vertices *)
v1GC = κGFunc[meshTriangles⟦j, 1, 1, 1⟧,
   meshTriangles⟦j, 1, 1, 2⟧, meshTriangles⟦j, 1, 1, 3⟧];
v1MC = κMFunc[meshTriangles⟦j, 1, 1, 1⟧, meshTriangles⟦j, 1, 1, 2⟧,
   meshTriangles⟦j, 1, 1, 3⟧];
v1k1 = v1MC + √(v1MC^2 - v1GC) ;
v1k2 = v1MC - √(v1MC^2 - v1GC) ;

v2GC = κGFunc[meshTriangles⟦j, 1, 2, 1⟧,
   meshTriangles⟦j, 1, 2, 2⟧, meshTriangles⟦j, 1, 2, 3⟧];
v2MC = κMFunc[meshTriangles⟦j, 1, 2, 1⟧, meshTriangles⟦j, 1, 2, 2⟧,
   meshTriangles⟦j, 1, 2, 3⟧];
v2k1 = v2MC + √(v2MC^2 - v2GC) ;
v2k2 = v2MC - √(v2MC^2 - v2GC) ;

v3GC = κGFunc[meshTriangles⟦j, 1, 3, 1⟧,
   meshTriangles⟦j, 1, 3, 2⟧, meshTriangles⟦j, 1, 3, 3⟧];
v3MC = κMFunc[meshTriangles⟦j, 1, 3, 1⟧, meshTriangles⟦j, 1, 3, 2⟧,
   meshTriangles⟦j, 1, 3, 3⟧];
v3k1 = v3MC + √(v3MC^2 - v3GC) ;
v3k2 = v3MC - √(v3MC^2 - v3GC) ;

aveGC = (v1GC + v2GC + v3GC) / 3;
aveMC = (v1MC + v2MC + v3MC) / 3;
avek1 = (v1k1 + v2k1 + v3k1) / 3;
avek2 = (v1k2 + v2k2 + v3k2) / 3;
triangle = {j, {v1, v2, v3}, centroid, area,
   minSideLengths, maxSideLengths, c1, c2, aveGC, aveMC, avek1, avek2};
trianglePropsRelaxed = AppendTo[trianglePropsRelaxed, triangle];
```

```
];

In[•]:= trianglePropsRelaxed[[1]] (* OK it works *)
trianglePropsRelaxed[[1000]]

Out[•]= {1, {{-3.68201, 1.13081, 1.07636},
    {-3.64141, 1.18985, 1.14949}, {-3.64824, 1.24253, 1.03082}},
  {-3.65722, 1.18773, 1.08555}, 0.00597972, 0.102378, 0.130016,
  0.0792815, -0.343119, 0.0794711, -0.343367, -0.147136, -0.539599}

Out[•]= {1000, {{-0.686816, -0.00481004, -1.03283},
    {-0.72825, -0.0744877, -1.16664}, {-0.830388, 0.0227752, -1.05235}},
  {-0.748485, -0.0188408, -1.08394}, 0.0110502, 0.147497, 0.18153,
  -2.45281, -1.23966, -2.38767, -1.20687, 0.752359, -3.1661}
```

Find the maximum and minimum values of the areas, maximum and minimum side lengths, and the four types of curvature, so each domain-span can be divided into sub-domains when required

```
In[•]:= areaMinR = Min@Table[trianglePropsRelaxed⟦i⟧ [[4]], {i, 1, l3}]
areaMaxR = Max@Table[trianglePropsRelaxed⟦i⟧ [[4]], {i, 1, l3}]
sideMinMinR = Min@Table[trianglePropsRelaxed⟦i⟧ [[5]], {i, 1, l3}]
sideMinMaxR = Min@Table[trianglePropsRelaxed⟦i⟧ [[5]], {i, 1, l3}]
sideMaxMinR = Min@Table[trianglePropsRelaxed⟦i⟧ [[6]], {i, 1, l3}]
sideMaxMaxR = Min@Table[trianglePropsRelaxed⟦i⟧ [[6]], {i, 1, l3}]

gaussCurvMinR = Min@Table[trianglePropsRelaxed⟦i⟧ [[9]], {i, 1, l3}]
(* Index 5 denotes the vertex-averaged Gaussian Curvature *)
gaussCurvMaxR = Max@Table[trianglePropsRelaxed⟦i⟧ [[9]], {i, 1, l3}]
meanCurvMinR = Min@Table[trianglePropsRelaxed⟦i⟧ [[10]], {i, 1, l3}]
(* Index 6 denotes the vertex-averaged Mean Curvature *)
meanCurvMaxR = Max@Table[trianglePropsRelaxed⟦i⟧ [[10]], {i, 1, l3}]
k1CurvMinR = Min@Table[trianglePropsRelaxed⟦i⟧ [[11]] // Re, {i, 1, l3}]
(* Index 7 denotes the vertex-averaged k1 Principal Curvature-1 *)
k1CurvMaxR = Max@Table[trianglePropsRelaxed⟦i⟧ [[11]] // Re, {i, 1, l3}]
k2CurvMinR = Min@Table[trianglePropsRelaxed⟦i⟧ [[12]] // Re, {i, 1, l3}]
(* Index 7 denotes the vertex-averaged k1 Principal Curvature-1 *)
k2CurvMaxR = Max@Table[trianglePropsRelaxed⟦i⟧ [[12]] // Re, {i, 1, l3}]

Out[•]= 0.000932643
Out[•]= 0.0351792
Out[•]= 0.0209717
Out[•]= 0.0209717
Out[•]= 0.0984294
Out[•]= 0.0984294
Out[•]= -4.9521
Out[•]= 4.63295
Out[•]= -9.15541
Out[•]= 0.595747
Out[•]= -0.314767
Out[•]= 1.28464
Out[•]= -18.2869
Out[•]= 0.524217
```

Graph a histogram of the triangle *areas* of the relaxed RBC

In[ ]:=
```
areasR = Flatten[Table[trianglePropsRelaxed⟦i, 4⟧, {i, 1, l3}]];
histArea = Histogram[areasR, 20, ChartStyle → RGBColor[1, 0, 0]]
```

Out[ ]=

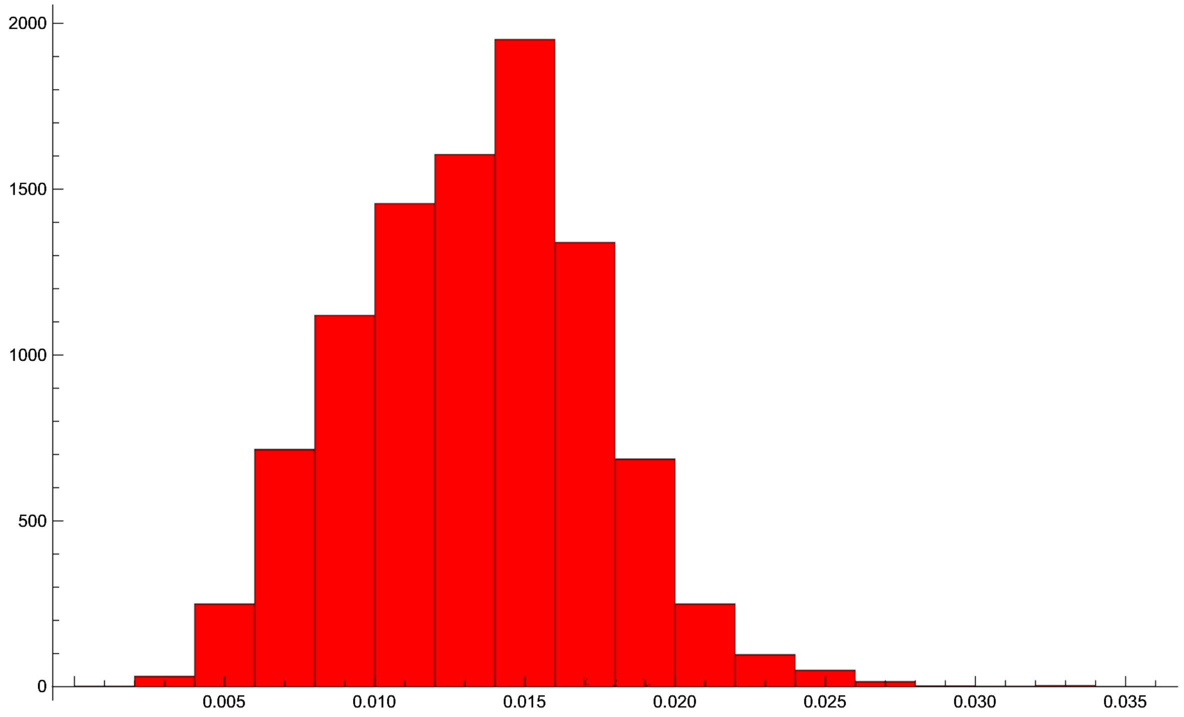

In[ ]:=
```
l3
RBCArea
RBCArea / l3 (* This area is the average area of a triangle
  in μm² or multipy by 10⁶ to get the number expressed in nm² *)
```

Out[ ]= 9584

Out[ ]= 127.894

Out[ ]= 0.0133445

Now process the distorted RBC...

In[ ]:=
```

ξ = 1.75;
θ = π / 2;

trianglePropsDistorted = {};
(* Meaning "triangle properties" for the relaxed RBC *)
For[j = 1, j ≤ l3, j++,
  v1 = meshTrianglesDistorted⟦j⟧⟦1⟧;
  (* meshTrianglesRelaxed⟦j⟧ is the jth triangle list,
  which is the three triple corrdinates so meshTriangles⟦j⟧⟦1⟧
   is the first vertex (triple-coordinate) *)
  v2 = meshTrianglesDistorted⟦j⟧⟦2⟧ ;
```

```
(* ...meshTriangles⟦j⟧⟦2⟧ is the second vertex (triple-coordinate) *)
v3 = meshTrianglesDistorted⟦j⟧⟦3⟧;
(* ...meshTriangles⟦j⟧⟦3⟧ is the third vertex (triple-coordinate) *)

centroid = (v1 + v2 + v3) / 3; (* Formula for the centroid
 of a triangle whose three vertex coordinates are known *)
area = (1 / 2) Cross[(v2 - v1), (v3 - v1)] // Norm;
(* Formula for the area of a triangle: half the normal of
   the cross product of the vectors of two of the sides *)
sideLength1 = Norm[v2 - v1];
sideLength2 = Norm[v3 - v2];
sideLength3 = Norm[v1 - v3];

minSideLengths = Min[sideLength1, sideLength2, sideLength3];
maxSideLengths = Max[sideLength1, sideLength2, sideLength3];

c1 = κGFunc[centroid⟦1⟧, centroid⟦2⟧, centroid⟦3⟧]; (* Apply the Gaussian
 Curvature function with the three coordinates of the centroid *)
c2 = κMFunc[centroid⟦1⟧, centroid⟦2⟧, centroid⟦3⟧]; (* Apply the Mean
 Curvature function with the three coordinates of the centroid *)

(* Apply the Gaussian and Mean Curvature formulae to the three vertices *)
v1GC = κGFunc[meshTriangles⟦j, 1, 1, 1⟧,
  meshTriangles⟦j, 1, 1, 2⟧, meshTriangles⟦j, 1, 1, 3⟧];
v1MC = κMFunc[meshTriangles⟦j, 1, 1, 1⟧, meshTriangles⟦j, 1, 1, 2⟧,
  meshTriangles⟦j, 1, 1, 3⟧];
v1k1 = v1MC + √(v1MC^2 - v1GC);
v1k2 = v1MC - √(v1MC^2 - v1GC);

v2GC = κGFunc[meshTriangles⟦j, 1, 2, 1⟧,
  meshTriangles⟦j, 1, 2, 2⟧, meshTriangles⟦j, 1, 2, 3⟧];
v2MC = κMFunc[meshTriangles⟦j, 1, 2, 1⟧, meshTriangles⟦j, 1, 2, 2⟧,
  meshTriangles⟦j, 1, 2, 3⟧];
v2k1 = v2MC + √(v2MC^2 - v2GC);
v2k2 = v2MC - √(v2MC^2 - v2GC);

v3GC = κGFunc[meshTriangles⟦j, 1, 3, 1⟧,
  meshTriangles⟦j, 1, 3, 2⟧, meshTriangles⟦j, 1, 3, 3⟧];
v3MC = κMFunc[meshTriangles⟦j, 1, 3, 1⟧, meshTriangles⟦j, 1, 3, 2⟧,
  meshTriangles⟦j, 1, 3, 3⟧];
v3k1 = v3MC + √(v3MC^2 - v3GC);
v3k2 = v3MC - √(v3MC^2 - v3GC);

aveGC = (v1GC + v2GC + v3GC) / 3;
aveMC = (v1MC + v2MC + v3MC) / 3;
avek1 = (v1k1 + v2k1 + v3k1) / 3;
```

```
  avek2 = (v1k2 + v2k2 + v3k2) / 3;
  triangle = {j, {v1, v2, v3}, centroid, area,
    minSideLengths, maxSideLengths, c1, c2, aveGC, aveMC, avek1, avek2};
  trianglePropsDistorted = AppendTo[trianglePropsDistorted, triangle];
 ];
```

Find the maximum and minimum values of the areas, maximum and minimum side lengths, and the four types of curvature, so each domain-span can be divided into 10 sub-domains

```
In[ ]:= areaMinD = Min@Table[trianglePropsDistorted⟦i⟧ [[4]], {i, 1, l3}]
areaMaxD = Max@Table[trianglePropsDistorted⟦i⟧ [[4]], {i, 1, l3}]
sideMinMinD = Min@Table[trianglePropsDistorted⟦i⟧ [[5]], {i, 1, l3}]
sideMinMaxD = Min@Table[trianglePropsDistorted⟦i⟧ [[5]], {i, 1, l3}]
sideMaxMinD = Min@Table[trianglePropsDistorted⟦i⟧ [[6]], {i, 1, l3}]
sideMaxMaxD = Min@Table[trianglePropsDistorted⟦i⟧ [[6]], {i, 1, l3}]

gaussCurvMinD = Min@Table[trianglePropsDistorted⟦i⟧ [[9]], {i, 1, l3}]
(* Index 5 denotes the vertex-averaged Gaussian Curvature *)
gaussCurvMaxD = Max@Table[trianglePropsDistorted⟦i⟧ [[9]], {i, 1, l3}]
meanCurvMinD = Min@Table[trianglePropsDistorted⟦i⟧ [[10]], {i, 1, l3}]
(* Index 6 denotes the vertex-averaged Mean Curvature *)
meanCurvMaxD = Max@Table[trianglePropsDistorted⟦i⟧ [[10]], {i, 1, l3}]
k1CurvMinD = Min@Table[trianglePropsDistorted⟦i⟧ [[11]] // Re, {i, 1, l3}]
(* Index 7 denotes the vertex-averaged k1 Principal Curvature-1 *)
k1CurvMaxD = Max@Table[trianglePropsDistorted⟦i⟧ [[11]] // Re, {i, 1, l3}]
k2CurvMinD = Min@Table[trianglePropsDistorted⟦i⟧ [[12]] // Re, {i, 1, l3}]
(* Index 7 denotes the vertex-averaged k1 Principal Curvature-1 *)
k2CurvMaxD = Max@Table[trianglePropsDistorted⟦i⟧ [[12]] // Re, {i, 1, l3}]

Out[ ]= 0.00119814

Out[ ]= 0.0429483

Out[ ]= 0.0309862

Out[ ]= 0.0309862

Out[ ]= 0.0834432

Out[ ]= 0.0834432

Out[ ]= -59.5666

Out[ ]= 3.06711

Out[ ]= -17.9091

Out[ ]= 0.760312

Out[ ]= -0.0827853
```

Out[ ]= 4.05391

Out[ ]= -35.8389

Out[ ]= 0.154814

Graph a histogram of the triangle *areas* of the distorted RBC

```
In[ ]:= areasD = Flatten[Table[trianglePropsDistorted⟦i, 4⟧, {i, 1, l3}]];
histArea = Histogram[areasD, 20, ChartStyle → RGBColor[0, 1, 0]]
```

Out[ ]=

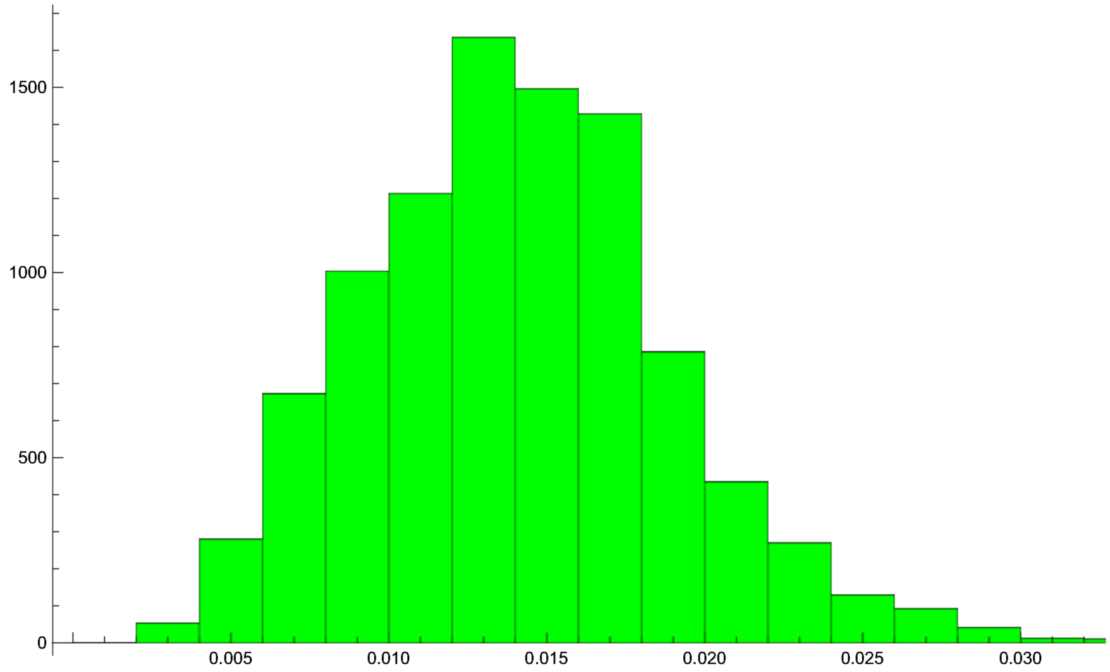

Area swept out by the arms of the Piezo1 triskelion of diameter 20 nm...in nm^2

```
In[ ]:= π 10^2 // N
(RBCArea 10^6 / l3) / (π 10^2)
```

Out[ ]= 314.159

Out[ ]= 42.4768

In[•]:=
```
trianglePropsDistorted⟦1⟧
Graphics3D[{RGBColor[1, 1, 0], Triangle[trianglePropsDistorted⟦1⟧⟦2⟧]}]
```

Out[•]=
```
{1, {{-2.78334, 0.85481, 1.88363},
  {-2.75265, 0.899444, 2.0116}, {-2.75781, 0.939268, 1.80393}},
 {-2.7646, 0.89784, 1.89972}, 0.00776378, 0.118903, 0.211517,
 0.0269951, -0.367046, 0.0191046, -0.264781, -0.0389262, -0.490637}
```

Out[•]=

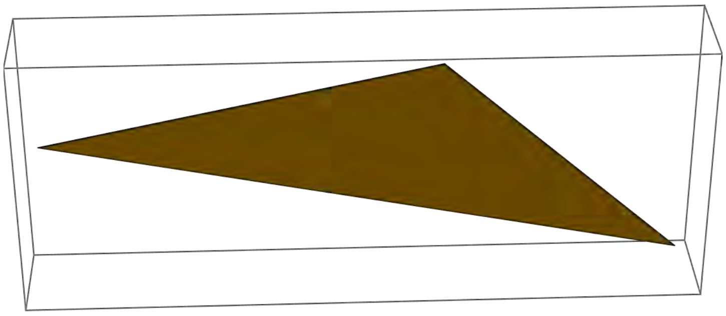

Now divide the list of triangle areas into 2 bins between areaMinD and areaMaxD, storing the indices for use in plotting the corresponding triangles

In[•]:=
```
binR[1] = {};
binR[2] = {};

For[j = 1, j ≤ l3, j++,   (* Tested before with only 5 triangles in the loop *)

  area = trianglePropsRelaxed⟦j, 4⟧;
  minP = areaMinR;
  maxP = areaMaxR;
  tT = area;
  delta = (maxP - minP) / 2.6;   (* Adjust delta to give 50:50 bin counts *)
  If[tT ≥ minP && tT < (minP + delta), binR[1] = AppendTo[binR[1], {j, tT}]];
  If[tT > (minP + delta) && tT < maxP, binR[2] = AppendTo[binR[2], {j, tT}]] ;
 ];
```

List the bin number and the number of elements in each bin

In[•]:=
```
Table[{i, Length@binR[i]}, {i, 1, 2}]
```

Out[•]=
```
{{1, 5288}, {2, 4295}}
```

Now graph the two bins to show the two groups of areas...first re-test the

graphing of the triangles from their vertices in trianglePropsRelaxed

In[ ]:=
```
relaxedRBC1 = Graphics3D@Table[Triangle[trianglePropsRelaxed⟦j⟧⟦2⟧], {j, 1, l3}]
```

Out[ ]=

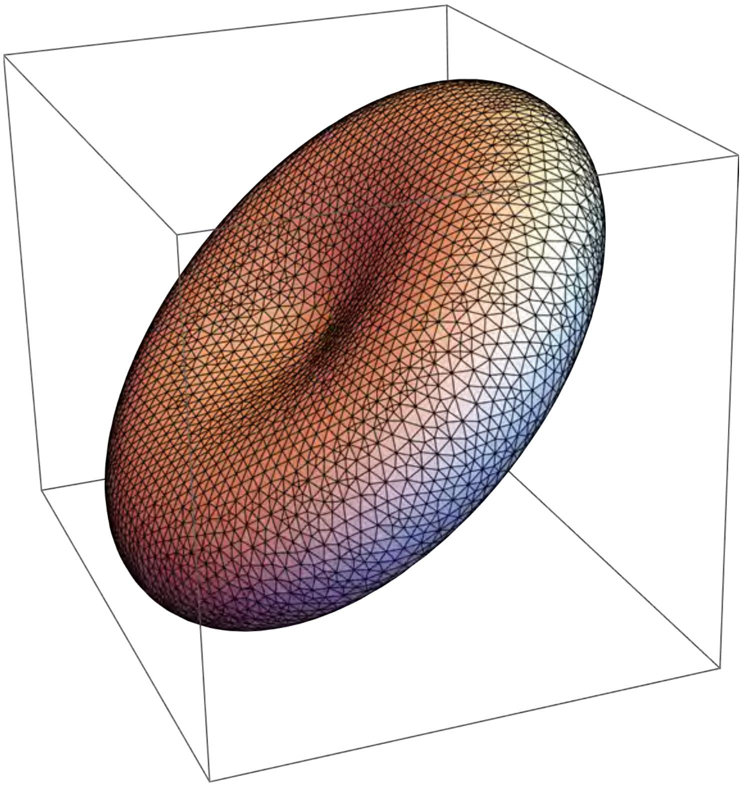

Graph the lowest area triangles first

In[ ]:=
```
indexNos = Table[binR[1]⟦j⟧⟦1⟧, {j, 1, Length@binR[1]}];
binSubSet = Triangle[trianglePropsRelaxed⟦#⟧⟦2⟧] & /@ indexNos;
meanBin[1] = Total[Transpose[binR[1]]⟦2⟧] / Length@binR[1];
gphRelaxed1 = Graphics3D[{Opacity[0.5], RGBColor[0, 1, 1], binSubSet}]
(* We see the outline of the stretched RBC *)

indexNos = Table[binR[2]⟦j⟧⟦1⟧, {j, 1, Length@binR[2]}];
binSubSet = Triangle[trianglePropsRelaxed⟦#⟧⟦2⟧] & /@ indexNos;
meanBin[2] = Total[Transpose[binR[2]]⟦2⟧] / Length@binR[2];
gphRelaxed2 = Graphics3D[{RGBColor[1, 0, 0], binSubSet}]
(* We see the outline of the stretched RBC *)

Show[{gphRelaxed1, gphRelaxed2}]
```

Out[ ]=

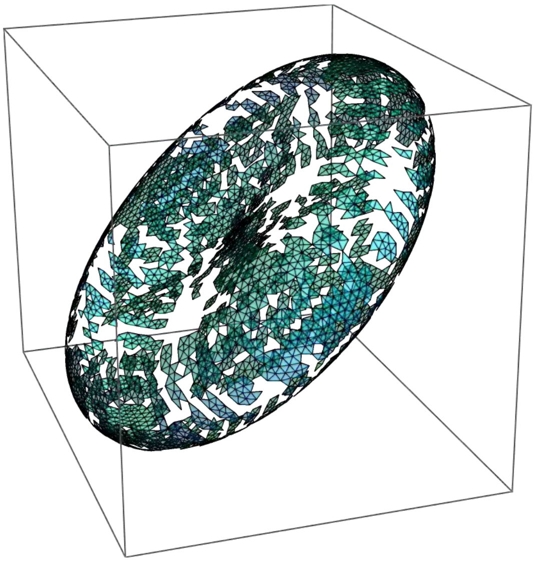

Out[ ]=

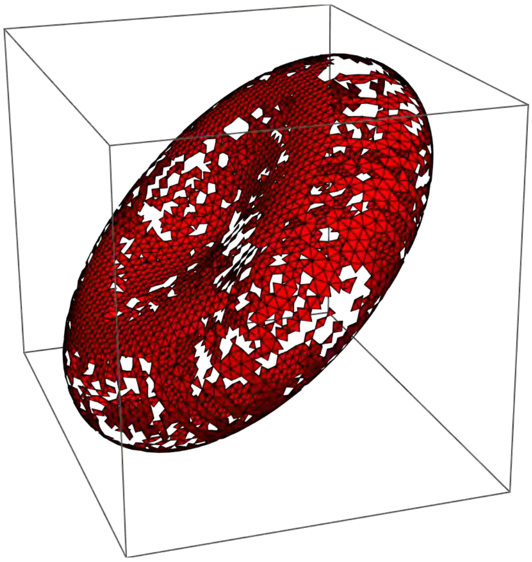

Out[ ]=

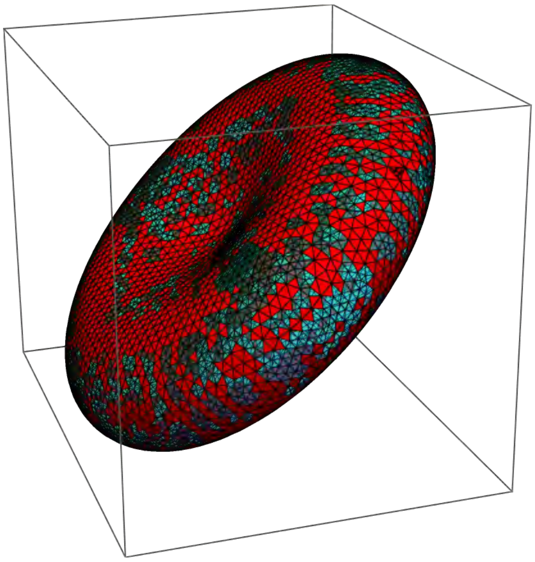

For the Distorted RBC divide the list of triangle areas into 2 bins between areaMin and areaMax, storing the indices for use in plotting the

corresponding triangles

In[ ]:=
```
binD[1] = {};
binD[2] = {};

For[j = 1, j ≤ l3, j++,

  area = trianglePropsDistorted⟦j, 4⟧;
  minP = areaMinD;
  maxP = areaMaxD;
  tT = area;
  delta = (maxP - minP) / 2.655;  (* Adjust delta to give 50:50 bin counts *)
  If[tT ≥ minP && tT < (minP + delta), binD[1] = AppendTo[binD[1], {j, tT}] ];
  If[tT > (minP + delta) && tT < maxP, binD[2] = AppendTo[binD[2], {j, tT}]] ;
 ];
```

List the bin number and the number of elements in each bin for the Distorted RBC

In[ ]:=
```
Table[{i, Length@binD[i]}, {i, 1, 2}]
```

Out[ ]=
```
{{1, 7055}, {2, 2528}}
```

Now graph the two bins to show the two groups of areas...first retest the graphing of the triangles from their vertices in trianglePropsRelaxed for the Distorted RBC

In[ ]:=
```
distortedRBC1 =
 Graphics3D[Table[Triangle[trianglePropsDistorted[[j]][[2]]], {j, 1, l3}],
  AspectRatio → Automatic]
```

Out[ ]=

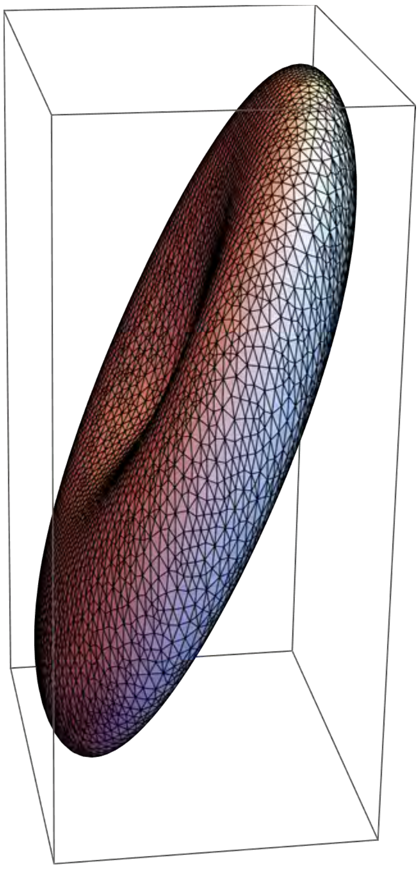

Graph the lowest area triangles first

In[•]:=
```
indexNos = Table[binD[1]⟦j⟧⟦1⟧, {j, 1, Length@binD[1]}];
binSubSet = Triangle[trianglePropsDistorted⟦#⟧⟦2⟧] & /@ indexNos;
meanBin[1] = Total[Transpose[binD[1]]⟦2⟧] / Length@binD[1];
gphDistorted1 = Graphics3D[{Opacity[0.5], RGBColor[1, 0, 1], binSubSet}]
(* We see the outline of the stretched RBC *)

indexNos = Table[binD[2]⟦j⟧⟦1⟧, {j, 1, Length@binD[2]}];
binSubSet = Triangle[trianglePropsDistorted⟦#⟧⟦2⟧] & /@ indexNos;
meanBin[2] = Total[Transpose[binD[2]]⟦2⟧] / Length@binD[2];
gphDistorted2 = Graphics3D[{RGBColor[1, 0, 0], binSubSet}]
(* We see the outline of the stretched RBC *)

Show[{gphDistorted1, gphDistorted2}]
Show[{gphRelaxed1, gphDistorted1}]
```

Out[•]=

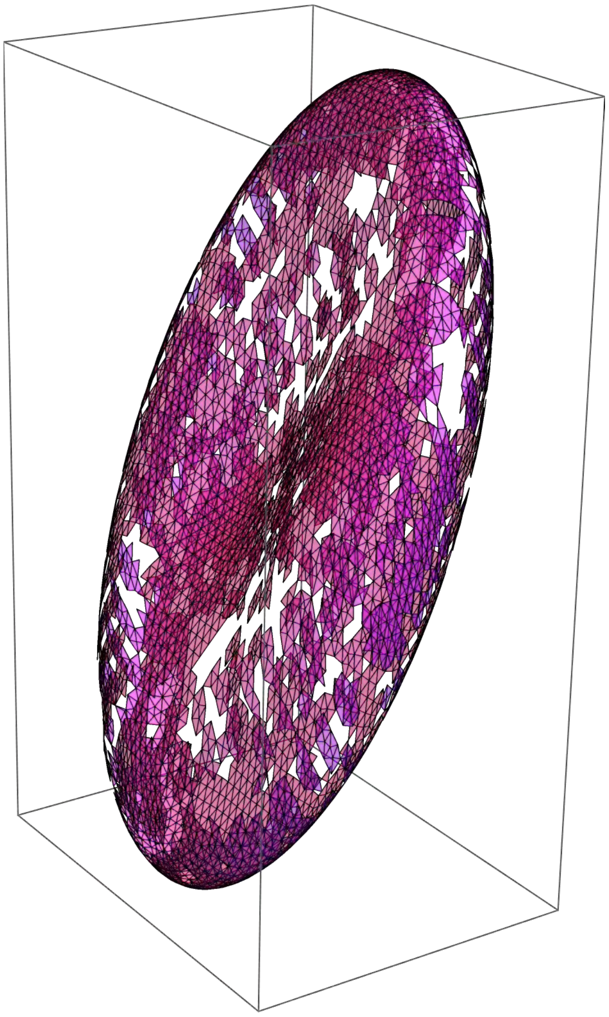

Out[ ]=

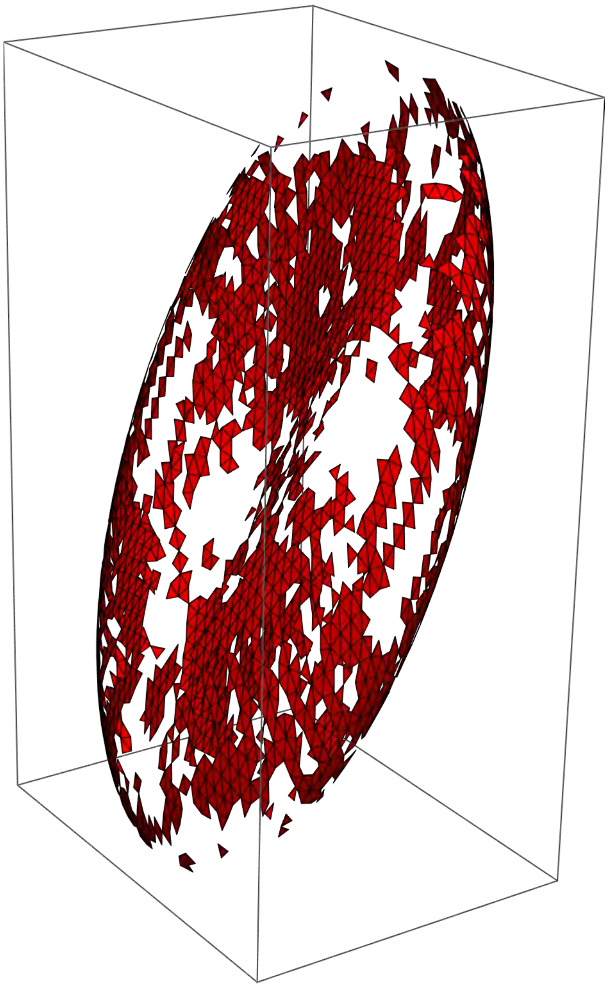

Out[ ]=

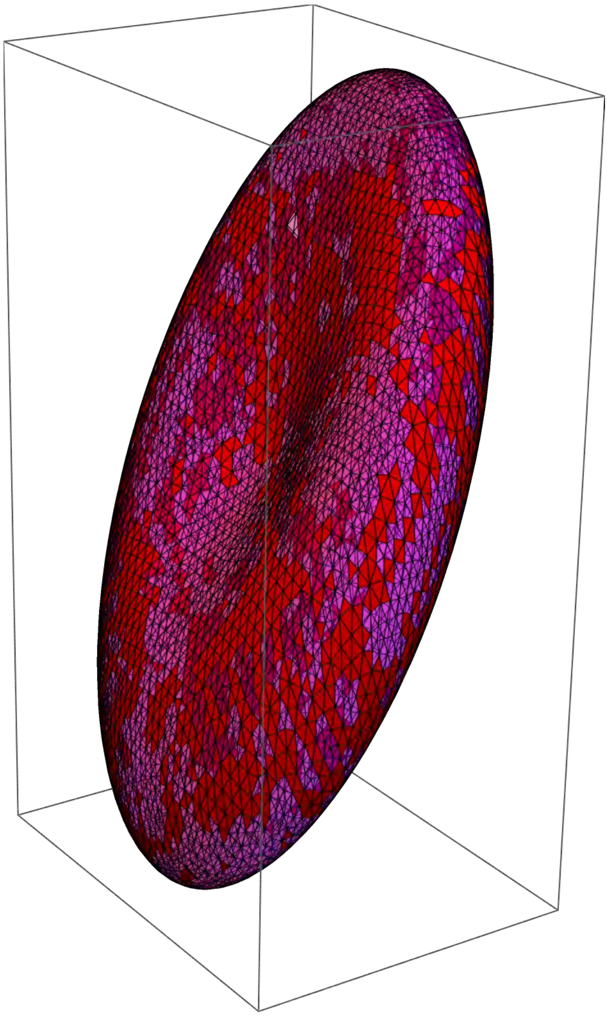

Out[ ]=

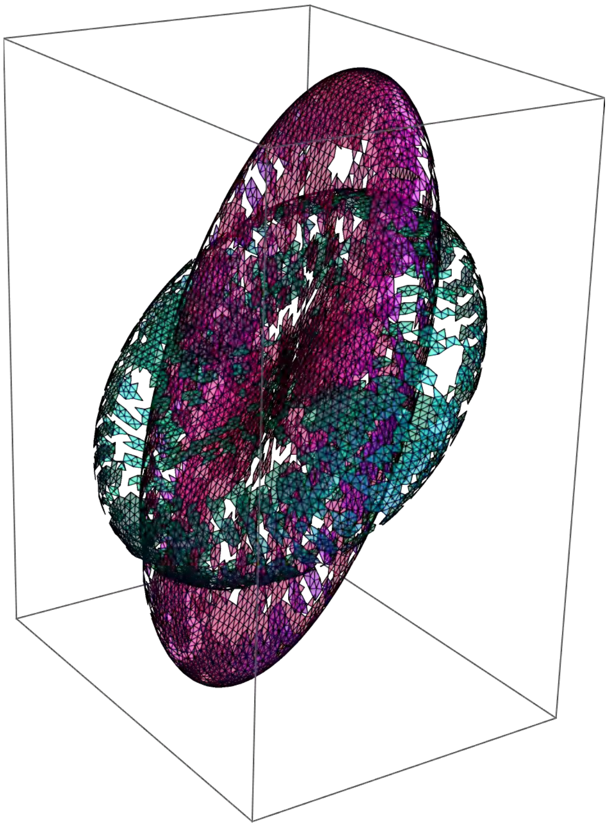

Perform mapping of DIFFERENCES in AREA before and after distortion. First, sort those triangles that have an increase in area from those with a decrease.

In[ ]:=

```
diff =
  Table[trianglePropsRelaxed[[j]][[4]] - trianglePropsDistorted[[j]][[4]], {j, 1, l3}];
minDiff = Min[diff];
maxDiff = Max[diff];
```

Sort the positive and negative changes in AREA

In[ ]:=
```
binAreaDiff[1] = {};
binAreaDiff[2] = {};

For[j = 1, j ≤ l3, j++,

  change = diff⟦j⟧;
  tT = change;
  If[tT ≤ 0.0, binAreaDiff[1] = AppendTo[binAreaDiff[1], {j, tT}] ];
  If[tT > 0, binAreaDiff[2] = AppendTo[binAreaDiff[2], {j, tT}]] ;
 ];
```

Graph the lowest difference triangles first

In[ ]:=
```
indexNos = Table[binAreaDiff[1]⟦j⟧⟦1⟧, {j, 1, Length@binAreaDiff[1]}];
binSubSet = Triangle[trianglePropsDistorted⟦#⟧⟦2⟧] & /@ indexNos;
meanBin[1] = Total[Transpose[binAreaDiff[1]]⟦2⟧] / Length@binAreaDiff[1];
gphDistortedDiff1 = Graphics3D[{Opacity[0.5], RGBColor[1, 0, 1], binSubSet}]
(* We see the outline of the stretched RBC *)

indexNos = Table[binAreaDiff[2]⟦j⟧⟦1⟧, {j, 1, Length@binAreaDiff[2]}];
binSubSet = Triangle[trianglePropsDistorted⟦#⟧⟦2⟧] & /@ indexNos;
meanBin[2] = Total[Transpose[binAreaDiff[2]]⟦2⟧] / Length@binAreaDiff[2];
gphDistortedDiff2 = Graphics3D[{RGBColor[1, 0, 0], binSubSet}]
(* We see the outline of the stretched RBC *)

Show[{gphDistortedDiff1, gphDistortedDiff2}]
```

Out[ ]=

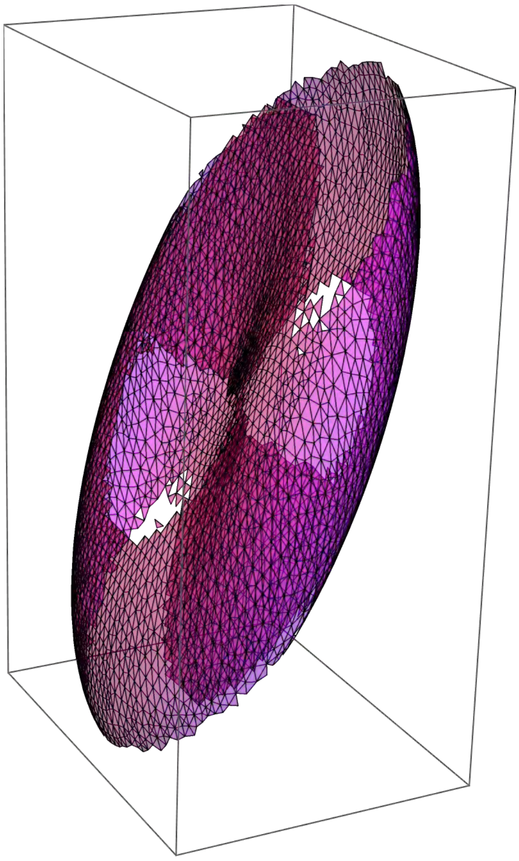

Out[ ]=

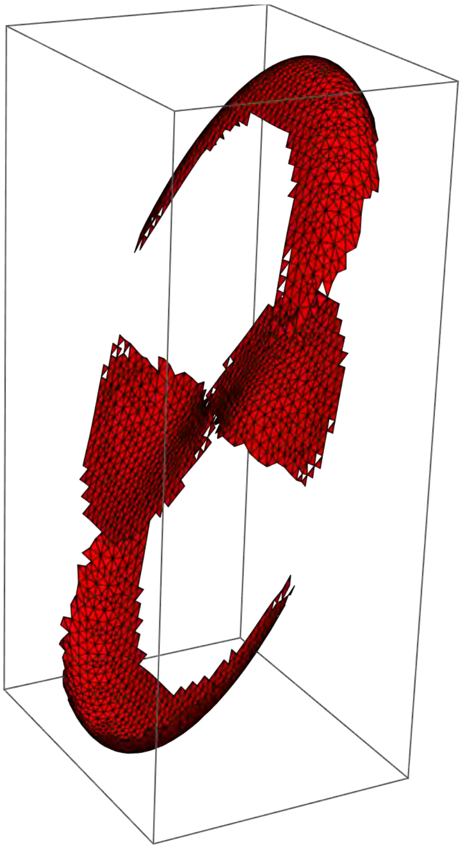

Out[ ]=

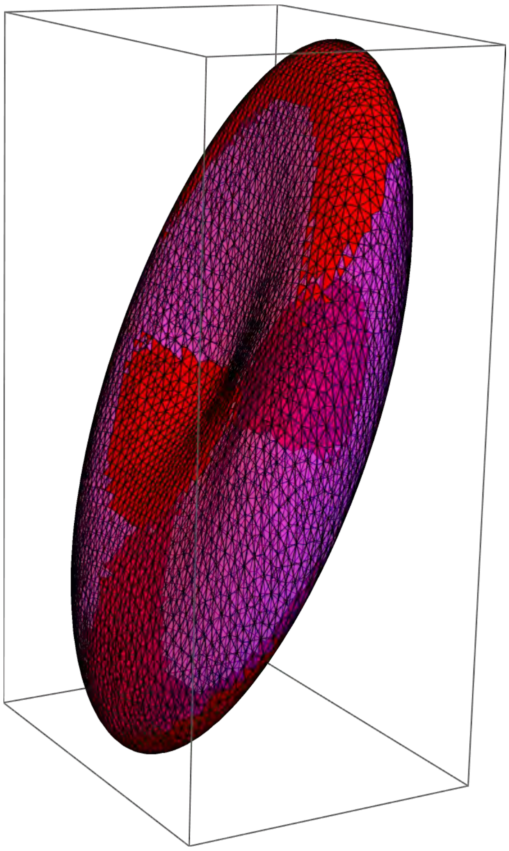

```
In[ ]:= (* triangle={j,{v1,v2,v3},centroid,area,
          minSideLengths, maxSideLengths,c1,c2,aveGC,aveMC,avek1,avek2} *)
```

Perform mapping of DIFFERENCES in K_G before and after distortion. First, make the list of differences of K_G

Sort the positive and negative changes in K_G

```
In[ ]:= diff =
          Table[trianglePropsRelaxed⟦j⟧⟦9⟧ - trianglePropsDistorted⟦j⟧⟦9⟧, {j, 1, l3}];

        bin[1] = {};
        bin[2] = {};

        For[j = 1, j ≤ l3, j++,
          change = diff⟦j⟧;
          tT = change;
          If[tT ≤ 0.0, bin[1] = AppendTo[bin[1], {j, tT}] ];
          If[tT > 0, bin[2] = AppendTo[bin[2], {j, tT}]] ;
         ];
```

Graph the lowest difference triangles first

```
In[•]:= indexNos = Table[bin[1]〚j〛〚1〛, {j, 1, Length@bin[1]}];
binSubSet = Triangle[trianglePropsDistorted〚#〛〚2〛] & /@ indexNos;
meanBin[1] = Total[Transpose[bin[1]]〚2〛] / Length@bin[1];
gphDistortedDiffKG1 = Graphics3D[{Opacity[0.5], RGBColor[1, 0, 1], binSubSet}]
(* We see the outline of the stretched RBC *)

indexNos = Table[bin[2]〚j〛〚1〛, {j, 1, Length@bin[2]}];
binSubSet = Triangle[trianglePropsDistorted〚#〛〚2〛] & /@ indexNos;
meanBin[2] = Total[Transpose[bin[2]]〚2〛] / Length@bin[2];
gphDistortedDiffKG2 = Graphics3D[{RGBColor[1, 0, 0], binSubSet}]
(* We see the outline of the stretched RBC *)

Show[{gphDistortedDiffKG1, gphDistortedDiffKG2}]


(* Conclusions: The first image shows the regions of the RBC where the
  Gaussian curvature is still negative after distortion of the RBC *)

(* Conclusions: The second image shows the regions of the RBC where the
  Gaussian curvature is still positive after distortion of the RBC *)
```

Out[•]=

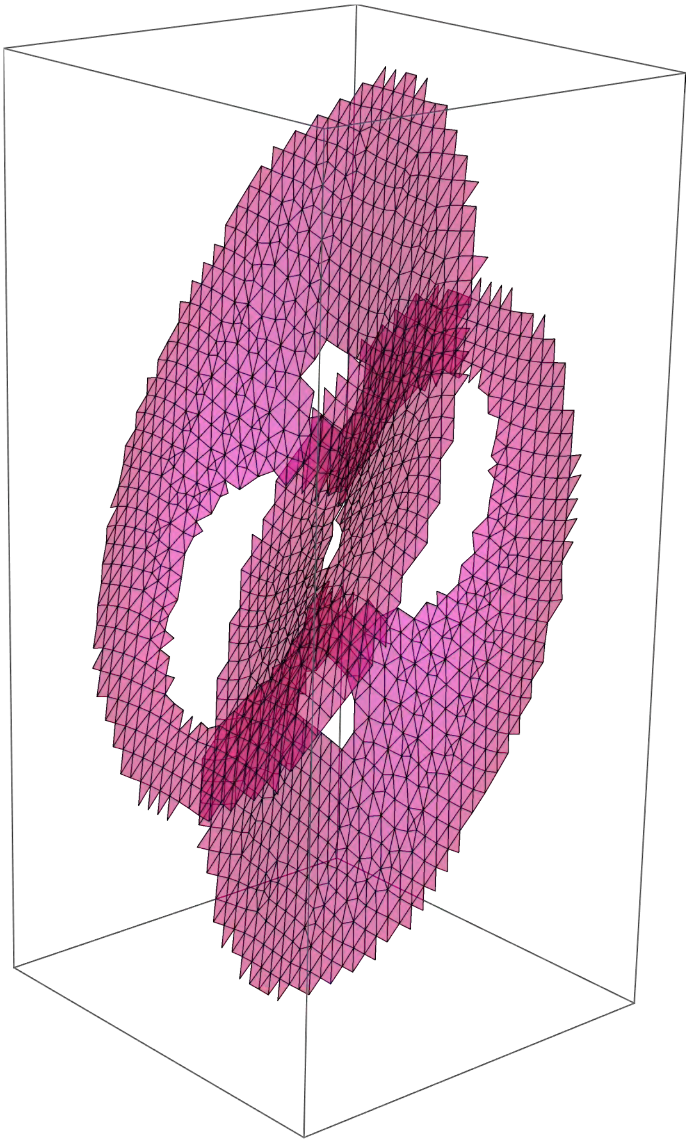

Out[•]=

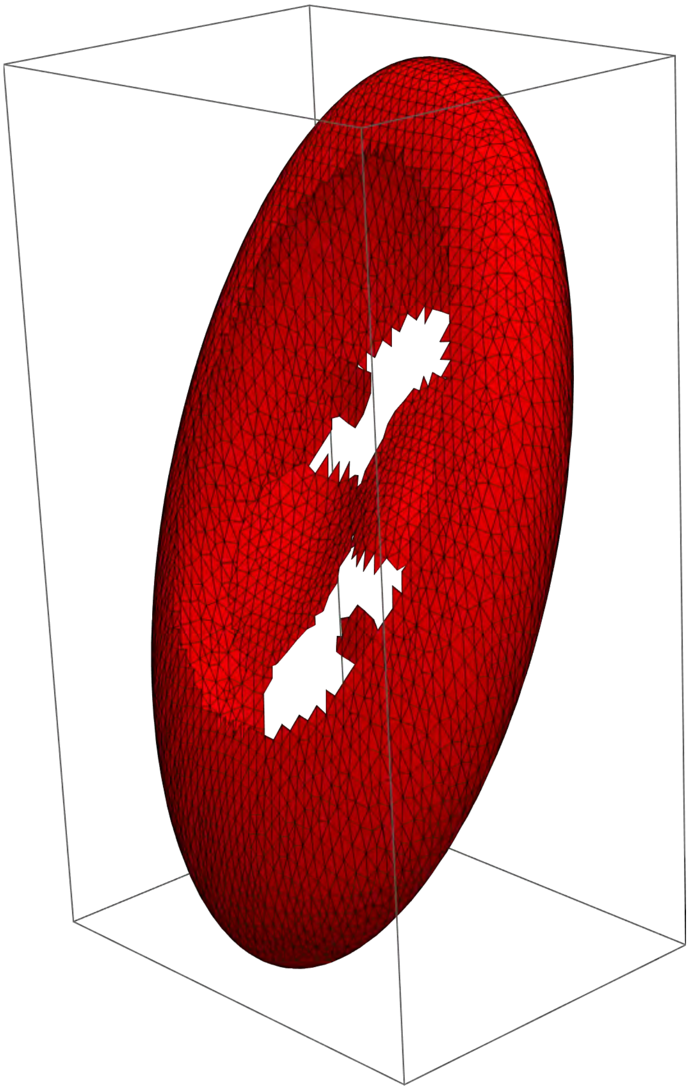

Out[ ]=

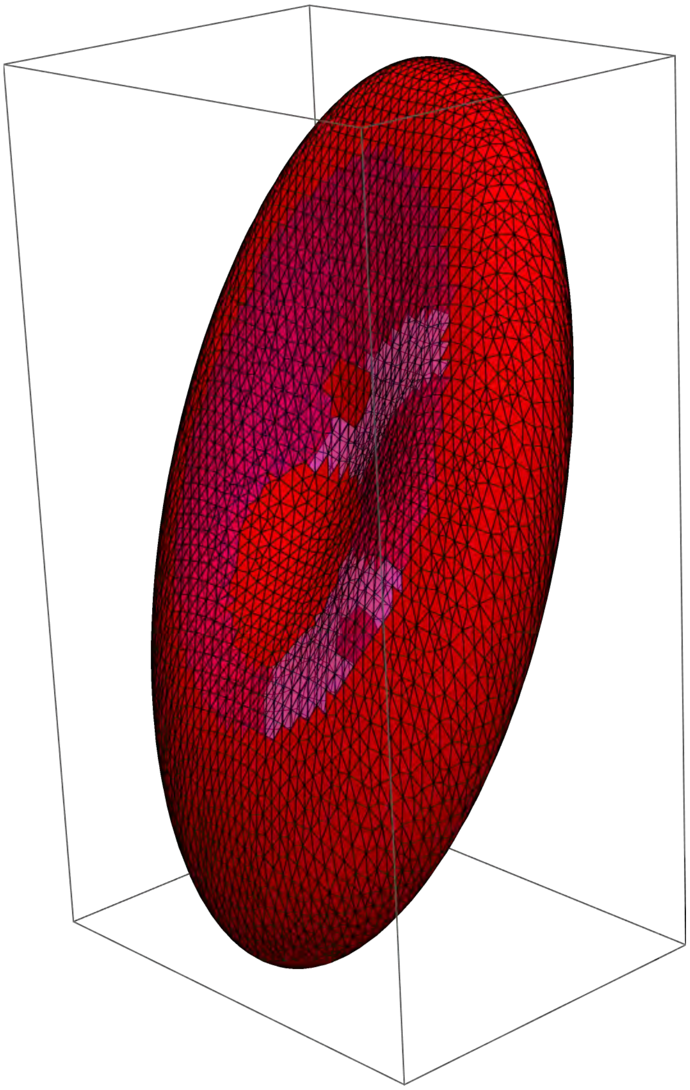

Perform mapping of *ABSOLUTE VALUE* DIFFERENCES in K_G before and after distortion. First, make the list of differences of K_G ...thus if the negative values are made MORE negative and if the positive values are made MORE positive...

In[ ]:=
```
diff = Table[Abs@trianglePropsRelaxed[[j]][[9]] -
    Abs@trianglePropsDistorted[[j]][[9]], {j, 1, l3}];

bin[1] = {};
bin[2] = {};

For[j = 1, j ≤ l3, j++,
  change = diff[[j]];
  tT = change;
  If[tT ≤ 0.0, bin[1] = AppendTo[bin[1], {j, tT}] ];
  If[tT > 0, bin[2] = AppendTo[bin[2], {j, tT}]] ;
 ];
```

Graph the lowest difference triangles first

```
In[]:= indexNos = Table[bin[1][[j]][[1]], {j, 1, Length@bin[1]}];
binSubSet = Triangle[trianglePropsDistorted[[#]][[2]]] & /@ indexNos;
meanBin[1] = Total[Transpose[bin[1]][[2]]] / Length@bin[1];
gphDistortedDiffKG1Abs =
 Graphics3D[{Opacity[0.5], RGBColor[0.3, 1, 0.3], binSubSet}]
(* We see the outline of the stretched RBC *)

indexNos = Table[bin[2][[j]][[1]], {j, 1, Length@bin[2]}];
binSubSet = Triangle[trianglePropsDistorted[[#]][[2]]] & /@ indexNos;
meanBin[2] = Total[Transpose[bin[2]][[2]]] / Length@bin[2];
gphDistortedDiffKG2Abs = Graphics3D[{RGBColor[1, 0.3, 0], binSubSet}]
(* We see the outline of the stretched RBC *)

Show[{gphDistortedDiffKG1Abs, gphDistortedDiffKG2Abs}]

(* Conclusions:
 The first image shows the regions of the RBC where the Gaussian curvature if
    negative in the relaxed RBC is MORE negative in the distorted RBC...
  or if positive is LESS positive after distortion of the RBC *)

(* Conclusions:
 The second image shows the regions of the RBC where the Gaussian
    curvature if negative is LESS negative after distortion...
  of if positive is MORE positive after distortion of the RBC *)
```

Out[ ]=

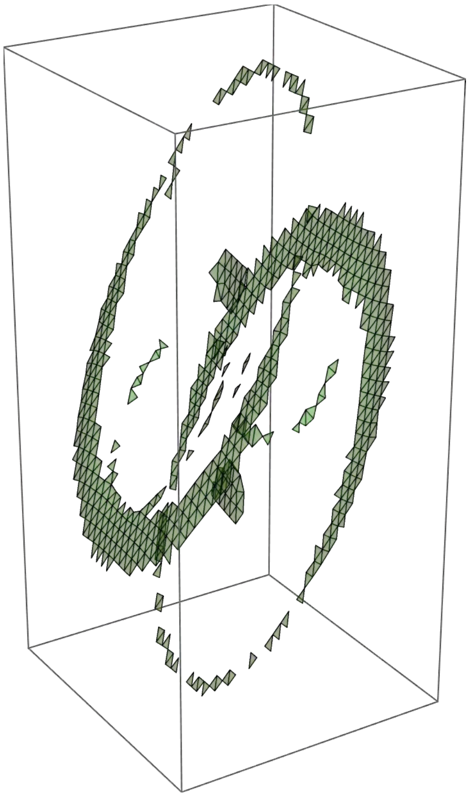

Out[ ]=

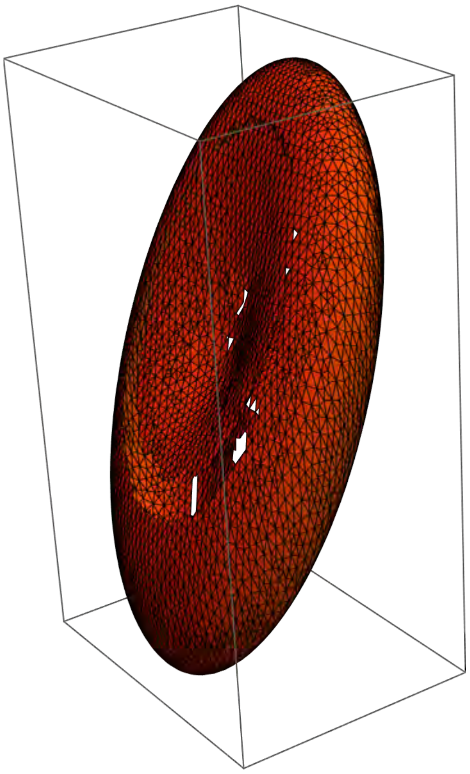

Out[ ]=

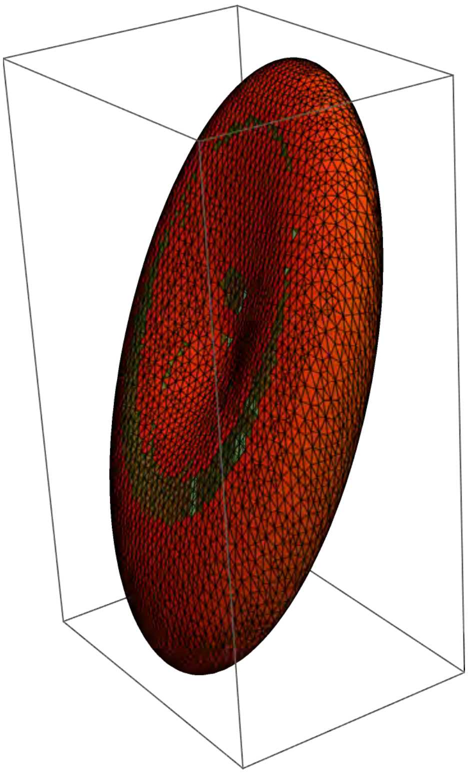

In[ ]:=
```
Show[{gphDistortedDiffKG1, gphDistortedDiffKG1Abs}]
```

Out[ ]=

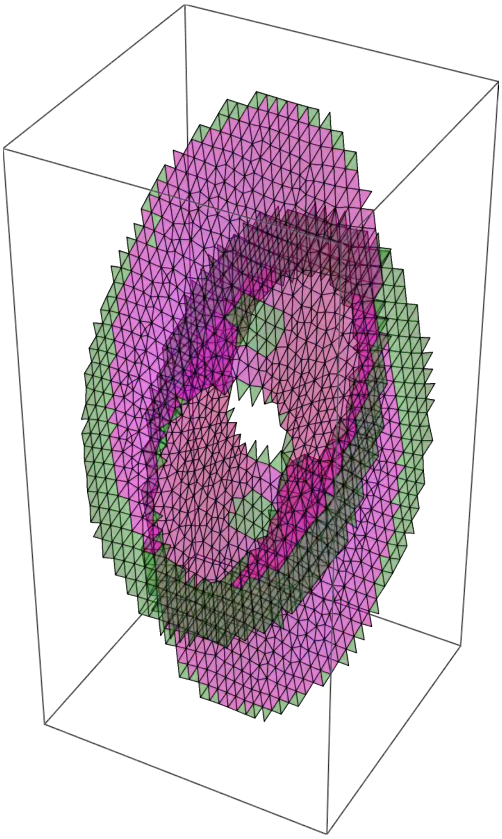

Perform mapping of DIFFERENCES in K_M before and after distortion. First, make the list of differences of K_M

In[∘]:=

```
diff =
  Table[trianglePropsRelaxed[[j]][[10]] - trianglePropsDistorted[[j]][[10]], {j, 1, l3}];
minDiff = Min[diff];
maxDiff = Max[diff];
```

Sort the positive and negative changes in K G

In[∘]:=

```
bin[1] = {};
bin[2] = {};

For[j = 1, j ≤ l3, j ++,
  change = diff[[j]];
  tT = change;
  If[tT ≤ 0.0, bin[1] = AppendTo[bin[1], {j, tT}] ];
  If[tT > 0, bin[2] = AppendTo[bin[2], {j, tT}]] ;
 ];
```

Graph the lowest difference triangles first

In[∘]:=

```
indexNos = Table[bin[1][[j]][[1]], {j, 1, Length@bin[1]}];
binSubSet = Triangle[trianglePropsDistorted[[#]][[2]]] & /@ indexNos;
meanBin[1] = Total[Transpose[bin[1]][[2]]] / Length@bin[1];
gphDistortedDiffKM1 = Graphics3D[{Opacity[0.5], RGBColor[1, 0, 1], binSubSet}]
(* We see the outline of the stretched RBC *)

indexNos = Table[bin[2][[j]][[1]], {j, 1, Length@bin[2]}];
binSubSet = Triangle[trianglePropsDistorted[[#]][[2]]] & /@ indexNos;
meanBin[2] = Total[Transpose[bin[2]][[2]]] / Length@bin[2];
gphDistortedDiffKM2 = Graphics3D[{RGBColor[1, 0, 0], binSubSet}]
(* We see the outline of the stretched RBC *)

Show[{gphDistortedDiffKM1, gphDistortedDiffKM2}]
```

Out[ ]=

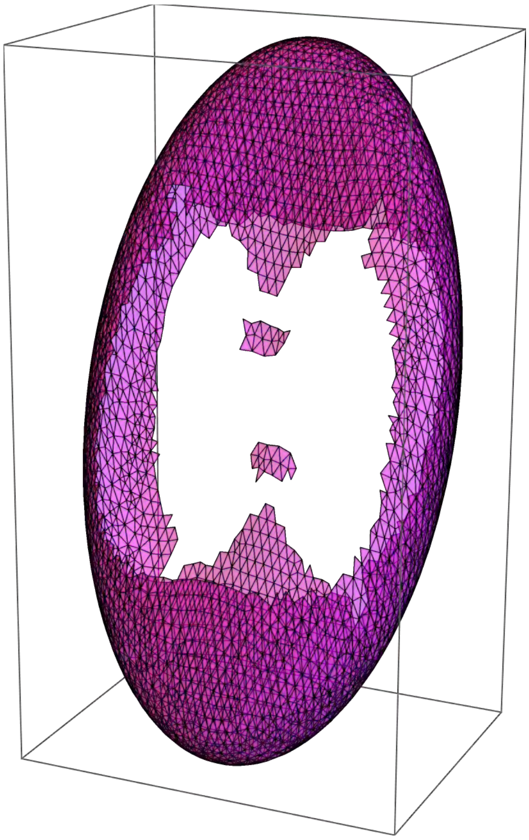

Out[ ]=

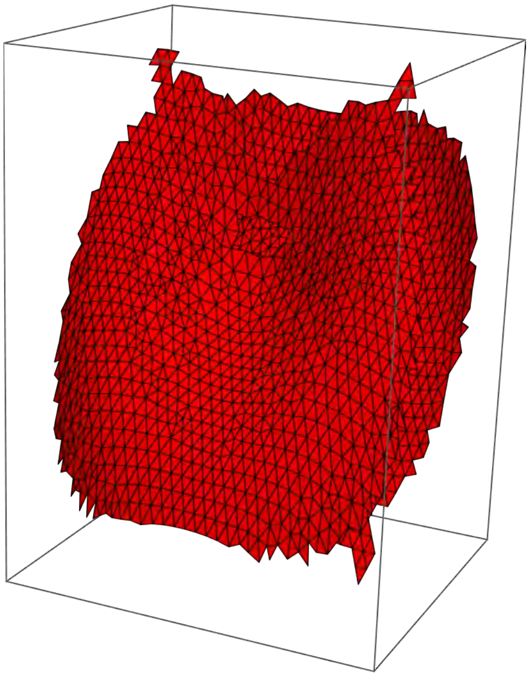

Out[ ]=

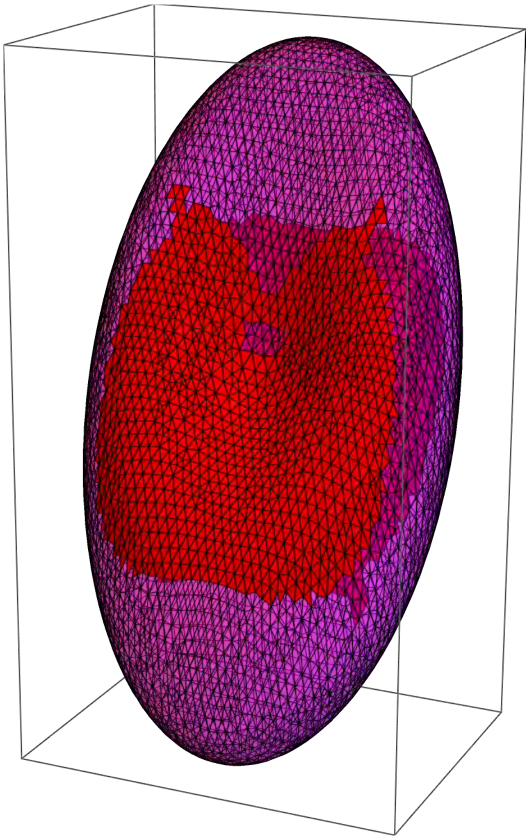

Perform mapping of DIFFERENCES in k_1 before and after distortion. First, make the list of differences of k_1

In[ ]:=
```
(*triangle={j,{v1,v2,v3},centroid,area,
  minSideLengths, maxSideLengths,c1,c2,aveGC,aveMC,avek1,avek2}*)
```

In[ ]:=
```
diff =
  Table[trianglePropsRelaxed⟦j⟧⟦11⟧ - trianglePropsDistorted⟦j⟧⟦11⟧, {j, 1, l3}];
minDiff = Min[diff];
maxDiff = Max[diff];
```

Sort the positive and negative changes in k_1

In[ ]:=
```
bin[1] = {};
bin[2] = {};

For[j = 1, j ≤ l3, j++,
  change = diff⟦j⟧;
  tT = change;
  If[tT ≤ 0.0, bin[1] = AppendTo[bin[1], {j, tT}] ];
  If[tT > 0, bin[2] = AppendTo[bin[2], {j, tT}]] ;
 ];
```

Graph the lowest difference triangles first

In[•]:=
```
indexNos = Table[bin[1]⟦j⟧⟦1⟧, {j, 1, Length@bin[1]}];
binSubSet = Triangle[trianglePropsDistorted⟦#⟧⟦2⟧] & /@ indexNos;
meanBin[1] = Total[Transpose[bin[1]]⟦2⟧] / Length@bin[1];
gphDistortedDiffk11 =
 Graphics3D[{Opacity[0.5], RGBColor[0.5, 0.5, 1], binSubSet}]
(* We see the outline of the stretched RBC *)

indexNos = Table[bin[2]⟦j⟧⟦1⟧, {j, 1, Length@bin[2]}];
binSubSet = Triangle[trianglePropsDistorted⟦#⟧⟦2⟧] & /@ indexNos;
meanBin[2] = Total[Transpose[bin[2]]⟦2⟧] / Length@bin[2];
gphDistortedDiffk12 = Graphics3D[{RGBColor[0.5, 0, 0.5], binSubSet}]
(* We see the outline of the stretched RBC *)

Show[{gphDistortedDiffk11, gphDistortedDiffk12}]
```

Out[•]=

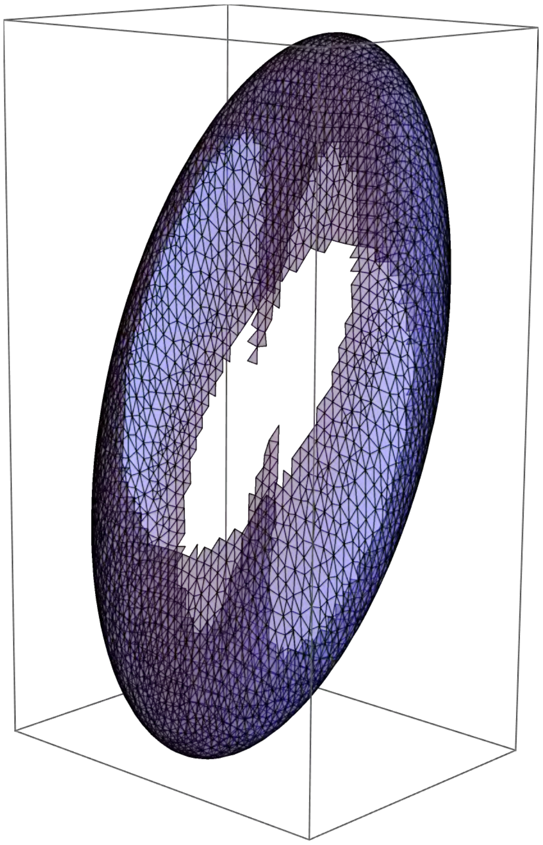

Out[ ]=

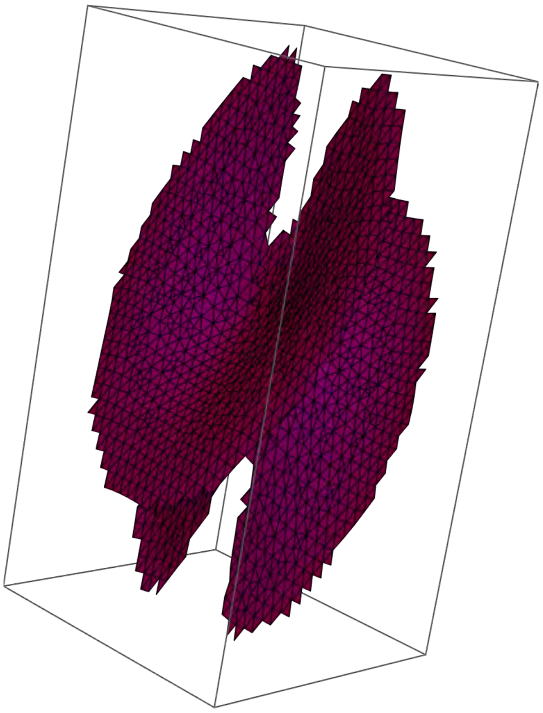

Out[ ]=

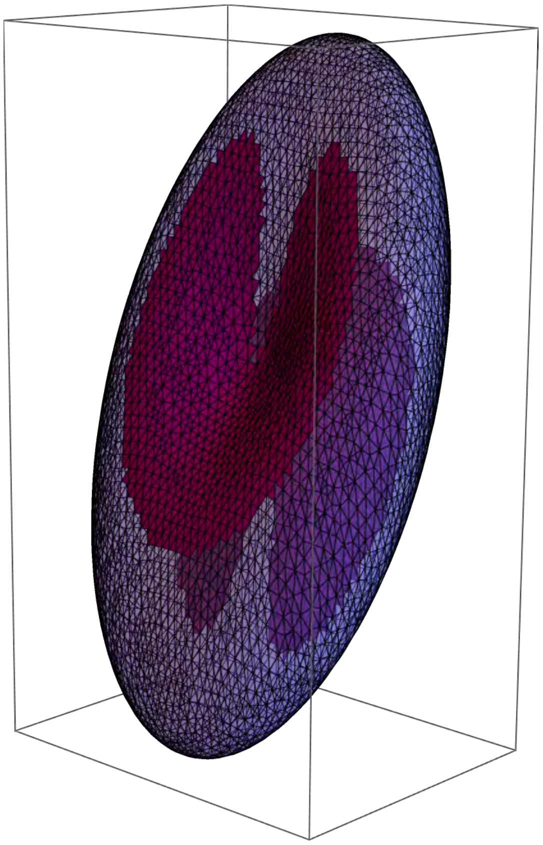

Perform mapping of DIFFERENCES in k $_2$ before and after distortion. First, make the list of differences of k $_2$

```
In[•]:= (*triangle={j,{v1,v2,v3},centroid,area,
          minSideLengths, maxSideLengths,c1,c2,aveGC,aveMC,avek1,avek2}*)
```

```
In[•]:=
        diff =
          Table[trianglePropsRelaxed⟦j⟧⟦12⟧ - trianglePropsDistorted⟦j⟧⟦12⟧, {j, 1, l3}];
        minDiff = Min[diff];
        maxDiff = Max[diff];
```

Sort the positive and negative changes in k $_2$

```
In[•]:= bin[1] = {};
        bin[2] = {};

        For[j = 1, j ≤ l3, j ++,
          change = diff⟦j⟧;
          tT = change;
          If[tT ≤ 0.0, bin[1] = AppendTo[bin[1], {j, tT}] ];
          If[tT > 0, bin[2] = AppendTo[bin[2], {j, tT}]] ;
         ];
```

Graph the lowest difference triangles first

```
In[•]:= indexNos = Table[bin[1]⟦j⟧⟦1⟧, {j, 1, Length@bin[1]}];
        binSubSet = Triangle[trianglePropsDistorted⟦#⟧⟦2⟧] & /@ indexNos;
        meanBin[1] = Total[Transpose[bin[1]]⟦2⟧] / Length@bin[1];
        gphDistortedDiffk21 =
         Graphics3D[{Opacity[0.5], RGBColor[0.5, 0.5, 0], binSubSet}]
        (* We see the outline of the stretched RBC *)

        indexNos = Table[bin[2]⟦j⟧⟦1⟧, {j, 1, Length@bin[2]}];
        binSubSet = Triangle[trianglePropsDistorted⟦#⟧⟦2⟧] & /@ indexNos;
        meanBin[2] = Total[Transpose[bin[2]]⟦2⟧] / Length@bin[2];
        gphDistortedDiffk22 = Graphics3D[{RGBColor[0.5, 1, 0.5], binSubSet}]
        (* We see the outline of the stretched RBC *)

        Show[{gphDistortedDiffk21, gphDistortedDiffk22}]
```

Out[ ]=

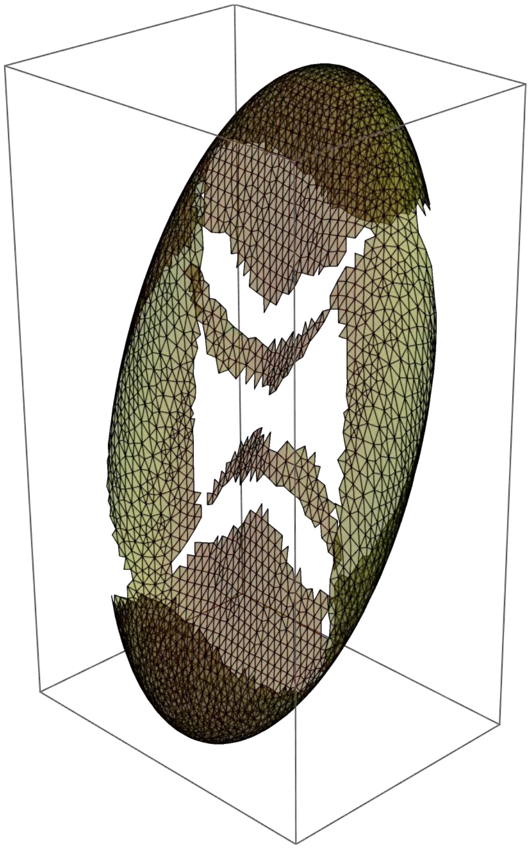

Out[ ]=

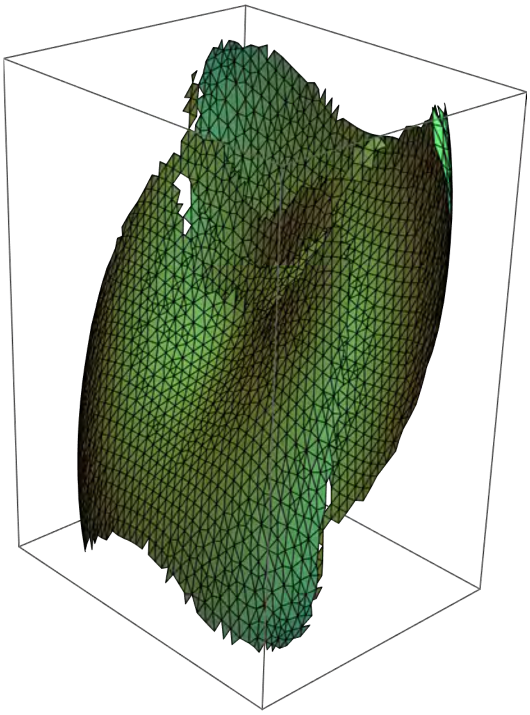

Out[ ]=

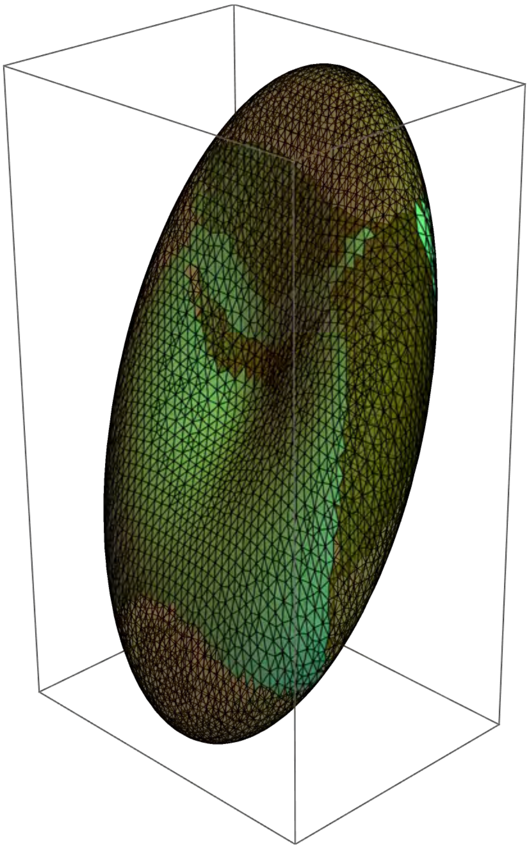

Supplement: Supplementary file 8 — Supplementary Information 8. [file 41598_2021_92699_MOESM8_ESM.pdf]
